# The state of plant photosystem II reaction centres affects the rate of non-photochemical quenching

**DOI:** 10.1038/s41477-026-02357-x

**Published:** 2026-08-14

**Authors:** Lennart A. I. Ramakers, Yuxi Niu, Jeremy Harbinson, Arjen Bader, Herbert van Amerongen

**Affiliations:** 1 Laboratory of Biophysics, Wageningen University, Wageningen, the Netherlands; 2 MicroSpectroscopy Research Facility, Wageningen University, Wageningen, the Netherlands

**Keywords:** Non-photochemical quenching, Abiotic

## Abstract

Plants employ non-photochemical quenching (NPQ) to protect their photosynthetic apparatus from photodamage. The response latency of NPQ following changes in light intensity is thought to significantly decrease photosynthetic efficiency. The amount of NPQ is commonly quantified from chlorophyll-fluorescence techniques using the Stern–Volmer equation, which requires fully closed reaction centres (RCs) of photosystem II, yielding NPQ in the absence of photochemical quenching (${\rm{NPQ}}^{\rm{Closed}}$). However, in nature, NPQ and photochemical quenching are normally present simultaneously. Therefore, to obtain a full understanding of this process, NPQ should also be explored when the RCs are open. Here we developed two methodologies to obtain NPQ in the presence of photochemistry (${\rm{NPQ}}^{\rm{Open}}$) using both fluorescence lifetime and fluorescence yield measurements. A detailed comparison in *Arabidopsis thaliana* plants reveals that the value of ${\mathrm{NPQ}}^{\mathrm{Open}}$ is ~35% lower than that of ${\rm{NPQ}}^{\rm{Closed}}$. This difference is consistently observed across all measurements and is seen both upon closing (${\rm{NPQ}}^{\rm{Open}}\to {\rm{NPQ}}^{\rm{Closed}}$) and upon reopening (${\mathrm{NPQ}}^{\mathrm{Closed}}\to {\mathrm{NPQ}}^{\mathrm{Open}}$) of the RCs. We show that this difference can be explained by the presence of RC-induced ‘instantaneous’ switching of the NPQ quenching rate. This means that, in plants, NPQ is much more economical than is widely believed, it is large when its presence is needed, and it decreases instantaneously when the need disappears.

## Abstract (web summary)

This study compares non-photochemical quenching (NPQ) rates in the presence and absence of photosystem II photochemistry. In *Arabidopsis thaliana*, the rate is ~35% lower in the presence of photochemistry, implying that NPQ is more economical than was previously believed.

## Main

Photosynthesis consists of a series of intricately intertwined processes that allow plants and other photosynthetic organisms to convert solar energy into chemical energy^1,2^. In plants, a key part of this process (often referred to as the ‘light’ reactions) involves a series of protein supercomplexes that allow photons to be absorbed and their energy to be utilized by photosystem I (PSI) and photosystem II (PSII) inducing linear electron transport from PSII to PSI, while simultaneously building up a proton gradient across the thylakoid membrane^2,3^. This proton gradient drives the formation of ATP, while the linear electron transport flux is utilized to generate NADPH. Together, both molecules are used to drive reactions within the Calvin–Benson–Bassham cycle (often referred to as the ‘dark’ reactions). One of these reactions, the carboxylation of ribulose-1,5-bisphosphate, fixes atmospheric CO_2_ to form phosphoglyceric acid, which is then reduced to form triose phosphate, the first carbohydrate produced during photosynthesis. This is then converted into other sugars and starch^1–3^. During the ‘light’ reactions, molecular O_2_ is produced at PSII as a waste product^3^. In parallel, the accumulation of protons in the lumenal space also leads to the activation of a number of photoprotective processes, commonly referred to as non-photochemical quenching (NPQ)^4–8^. Increases in light intensity lead to a higher rate of light absorption in the antenna complexes associated with the photosystems as well as further activation of the processes underpinning NPQ via a further decrease in lumenal pH. Once the intensity increases to the point that the rate of absorption is greater than the rate at which all of these excitations can be photochemically used by the photosystems, the response of photosynthesis to irradiances becomes increasingly dominated by NPQ. In PSII, NPQ consists of a range of mechanisms that lead to quenching of the excess excitations^4,7–22^. Over decades of research, several distinct processes underpinning NPQ have been identified^4,7–22^. The most important of these processes are triggered by the accumulation of protons in the lumenal space, and while there still remain unanswered questions about the molecular mechanisms of these processes, it is clear that their activation leads to the formation of NPQ quenching sites within PSII. These sites lead to a portion of the excitations being quenched, reducing the probability that these excitations encounter a closed PSII reaction centre (RC) (^PSII^RC) and thus diminishing the chance that such an encounter leads to the formation of highly reactive oxygen species. NPQ is mediated via several different molecular processes, and the fastest of these are triggered by the acidification of the lumenal space, which leads to PsbS protonation and the accumulation of zeaxanthin, acting together to provide photoprotection. While fast, these processes induce and relax on timescales of up to several minutes and therefore exhibit significant response latency to several naturally occurring sources of fluctuation in light intensity (such as sun and wind flecks). As such, NPQ is seen as a potentially wasteful process, which protects the plant at the cost of photosynthetic efficiency^23^. It was originally estimated that an increase of photosynthetic efficiency together with an increase in biomass of 30% could be reached by speeding up induction and relaxation of NPQ^24^. This led to several experimental studies that attempted to alleviate the NPQ response latency and, thus, improve the overall photosynthetic yield^25–30^. Some studies did show that this approach could improve the biomass yield, yielding up to a ~30% increase^26,30^, but other studies did not observe any improvement^25,27,28^. In these studies, NPQ is often considered to be an indiscriminate form of photoprotection, which, once induced, quenches excitations in PSII with a fixed rate constant^4,7–22^. However, recently, other studies have indicated that this might not be accurate^31,32^, and a study on *Spinacea oleracea*, employing ultrafast fluorescence spectrometry, suggested that the quenching rate associated with NPQ was lower in the presence of open ^PSII^RCs than closed ^PSII^RCs^31^. This further supports the idea that NPQ is more economical in nature than originally envisioned; however, it would also reduce the increase of yield in biomass previously predicted^24^.

NPQ is commonly explored using fluorescence spectroscopy, which typically requires the measurement of either the fluorescence lifetimes^22,33,34^ or yields^35–37^ in photosynthetic organisms before, during or after illumination. To obtain NPQ values, these lifetimes or yields are normally measured for fully closed ^PSII^RCs. These measurements can then be used to calculate NPQ using either the temporal (using $${\tau }_{{F}_{{\rm{m}}}}$$ and $${\tau }_{{F}_{{\rm{m}}}^{{\prime} }}$$: the fluorescence lifetimes without and with NPQ, respectively) or yield (using $${F}_{{\rm{m}}}$$ and $${F}_{{\rm{m}}}^{{\prime} }$$: the fluorescence yields without and with NPQ, respectively) version of the Stern–Volmer equation ($${\rm{NPQ}}=\left({\tau }_{{F}_{\rm{m}}}-{\tau }_{{F}_{\rm{m}}^{{\prime} }}\right)/{\tau }_{{F}_{\rm{m}}^{{\prime} }}=\left({F}_{\rm{m}}-{F}_{\rm{m}}^{{\prime} }\right)/{F}_{\rm{m}}^{{\prime} }$$)^35,37,38^. As this most common method for calculating the NPQ value relies on all the ^PSII^RCs being closed, this value is always obtained in the absence of the photochemical quenching (hereafter this value will be referred to as $${\rm{NPQ}}^{\rm{Closed}}$$, which yields the associated quenching rate $${k}_{\rm{NPQ}}^{\rm{Closed}}$$). However, under natural conditions, NPQ is almost always active in the presence of open ^PSII^RCs, indicating that, to fully understand NPQ, it is important to also measure its value in the presence of photochemical quenching. For fully open ^PSII^RCs, it is possible to derive an $${F}_{\rm{o}}$$ and $${F}_{{\rm{o}}}^{{\prime} }$$ version of the Stern–Volmer equation ($${\rm{NPQ}}^{\rm{Open}}=\left[\left({F}_{\rm{o}}-{F}_{\rm{o}}^{{\prime} }\right)/{F}_{\rm{o}}^{{\prime} }\right]\left({F}_{\rm{m}}/{F}_{\rm{o}}\right)$$$$=\left[\left({\tau }_{{F}_{\rm{o}}}-{\tau }_{{F}_{\rm{o}}^{{\prime} }}\right)/{\tau }_{{F}_{\rm{o}}^{{\prime} }}\right]\left({\tau }_{{F}_{\rm{m}}}/{\tau }_{{F}_{\rm{o}}}\right)$$) allowing the NPQ value to be calculated in the presence of photochemical quenching (outlined in Supplementary Note 3.2; hereafter, this value will be referred to as $${\rm{NPQ}}^{\rm{Open}}$$, which yields the associated quenching rate $${{k}}^{\mathrm{Open}}_{\mathrm{NPQ}}$$). Finally, it is possible to calculate a value of NPQ in the presence of partially open ^PSII^RCs. Here, $${F}^{{\prime} }$$ (or $${\tau }_{{F}^{{\prime} }}$$), the instantaneous fluorescence yield (or lifetime), is used to calculate the overall combined quenching ($${\rm{CQ}}$$) arising from the photochemical ($${\mathrm{PQ}}^{* }$$) and non-photochemical ($${\mathrm{NPQ}}^{{\prime} }$$) pathways ($$\mathrm{CQ}=\left({F}_{{\rm{m}}}-{F}^{^{\prime} }\right)/{F}^{{\prime} }={\mathrm{NPQ}}^{{\prime} }+{\mathrm{PQ}}^{* }$$; outlined in Supplementary Note 3.3). This combined quenching is calculated in the presence of the almost fully or partially open ^PSII^RCs. Interestingly, $${\mathrm{PQ}}^{* }$$ is directly proportional to the fraction of open ^PSII^RCs ($$\mathrm{qP}$$, a proxy for the number of open ^PSII^RCs (Supplementary Note 3.1); alternatively, $${\rm{qL}}$$ could also be used as a proxy for the number of open ^PSII^RCs), allowing an NPQ value to be calculated in the presence of open ^PSII^RCs (hereafter referred to as $${\rm{NPQ}}^{{\prime} }$$, which yields the associated quenching rate $${{k}}^{{\prime} }_{{\rm{NPQ}} }$$). As $${F}^{{\prime} }$$ (or $${\tau }_{{F}^{{\prime} }}$$) represents average measurements, which includes both open (which exhibit $${\rm{NPQ}}^{\rm{Open}}$$) and closed (which exhibit $${\mathrm{NPQ}}^{\mathrm{Closed}}$$) ^PSII^RCs, it is thought that $${\rm{NPQ}}^{{\prime} }$$ is a weighted average between these two values. Specifically, the weighting in this average represents the portion of open ^PSII^RCs during the measurement of $${F}^{{\prime} }$$ (or $${\tau }_{{F}^{{\prime} }}$$), which can be approximated by $${\rm{qP}}$$ (refs. ^35,39^). Because $${\rm{PQ}}^{* }$$ is directly proportional to the fraction of open ^PSII^RCs, the ratio between this value and $$\mathrm{PQ}$$ (its dark-adapted counterpart, $$\mathrm{PQ}=\left(F_{\rm{m}}-F_{\rm{o}}\right)/F_{\rm{o}}$$) can be used to obtain $${\rm{qP}}$$, meaning that the methodology can also be used to obtain $${\rm{NPQ}}^{\rm{Open}}$$ (outlined in Supplementary Note 3.8).

Here, we determine and compare values of ${\rm{NPQ}}^{\rm{Closed}}$ and ${\mathrm{NPQ}}^{\mathrm{Open}}$ for wild-type (wt) *Arabidopsis thaliana*, obtained via ultrafast fluorescence and light response curve measurements. Both the fluorescence lifetime and yield show a distinct discrepancy between the values of ${\rm{NPQ}}^{\rm{Closed}}$ and ${\mathrm{NPQ}}^{\mathrm{Open}}$, with ${\mathrm{NPQ}}^{\mathrm{Open}}$ < ${\rm{NPQ}}^{\rm{Closed}}$ ($\Rightarrow {{{k}}}_{\mathrm{NPQ}}^{\mathrm{Open}}$ < ${k}_{\mathrm{NPQ}}^{\mathrm{Closed}}$). Combined, the lifetime and yield measurements reveal that this distinct discrepancy is seen immediately upon reopening of ^PSII^RCs at the end of the illumination as well as upon the closure of the ^PSII^RCs (for example, by a saturating pulse). This latter method eliminates fast NPQ relaxation as a possible source of the discrepancy and shows that the change in the value of NPQ occurs on a subsecond timescale. Other possible sources for the observed discrepancy between ${\rm{NPQ}}^{\rm{Closed}}$ (or ${k}_{\mathrm{NPQ}}^{\mathrm{Closed}}$) and ${\rm{NPQ}}^{\rm{Open}}$ (or ${{k}}^{\mathrm{Open}}_{\mathrm{NPQ}}$) were explored using numerical model simulations based on the Butler model, but only the ^PSII^RC-induced NPQ quenching rate switching is fully consistent with the experimental results. This rate switching is further accompanied by the appearance of a far-red emission band in the ${F}_{{\rm{m}}}^{{\prime} }$ PSII spectrum, as observed previously^31^.

## Results

### Ultrafast fluorescence NPQ induction period

**Fig. 1:**
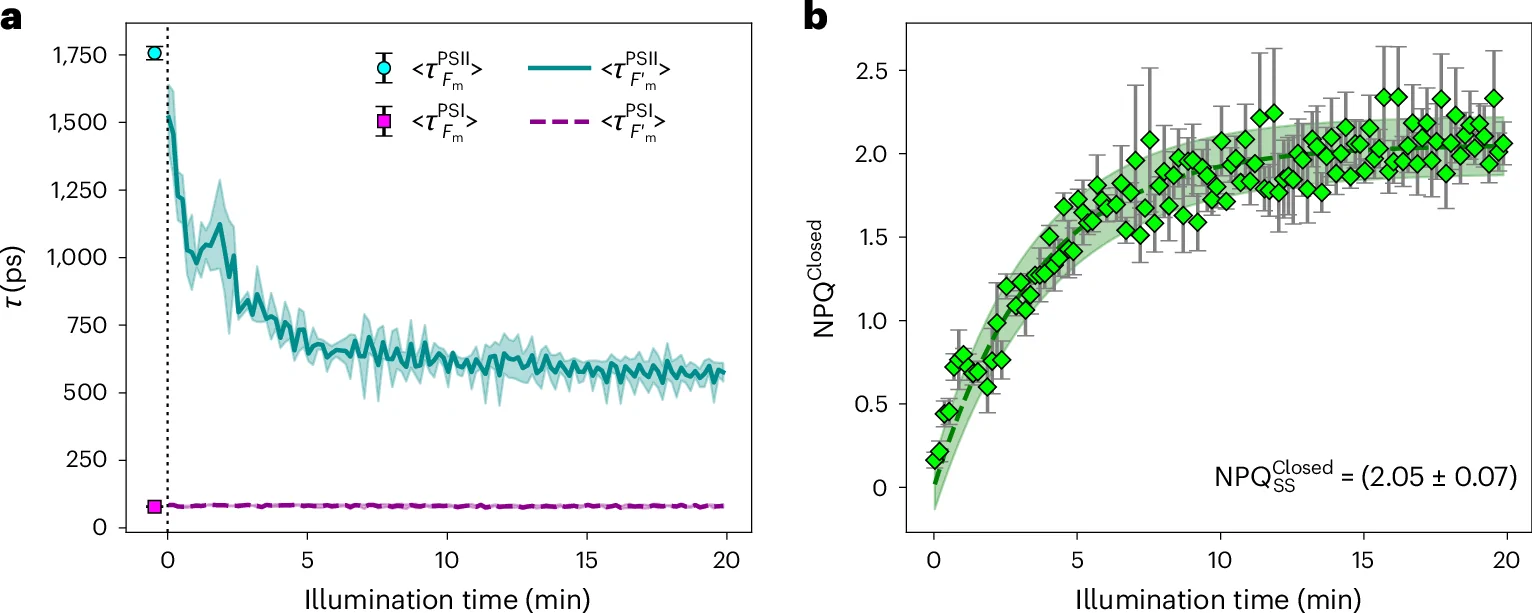
Changes in average PSII and PSI fluorescence lifetime during NPQ induction. **a**, The average ${F}_{\rm{m}}^{{\prime} }$ lifetimes of PSII (solid line) and PSI (dashed line) plotted as a function of illumination time (n=3; outlined in Supplementary Note 4), with the average ${F}_{\rm{m}}$ lifetimes of PSII (cyan circle) and PSI (magenta square) in fully dark-adapted leaves (n=3). **b**, ${\rm{NPQ}}^{\rm{Closed}}$ induction curve, calculated using the average PSII lifetimes (n=3, where the error bars show the associated standard error), with an insert showing the steady-state value (${\rm{NPQ}}_{\rm{SS}}^{\rm{Closed}}$). All wt *A. thaliana* plants were fully dark-adapted overnight before measurement and illuminated at an actinic light intensity of 14.1 mW (spectrum shown in Supplementary Fig. 1b).

In the ultrafast measurements, chlorophyll fluorescence spectra were measured for leaves with fully open and closed ^PSII^RCs in the absence (${F}_{\rm{o}}$ and ${F}_{{\rm{m}}}$, respectively) and presence (${F}_{{\rm{o}}}^{{\prime} }$ and ${F}_{{\rm{m}}}^{{\prime} }$, respectively) of NPQ. In all of these measurements, NPQ was induced using a blue actinic light source (14.1 mW; spectrum shown in Supplementary Fig. 1b). To explore how these spectra change during this illumination period, a series of low signal-to-noise ratio (SNR) ultrafast fluorescence spectra were measured during this induction period for leaves with fully closed ^PSII^RCs. These spectra were deconvoluted (using an ${F}_{{\rm{m}}}$-guided version of independent component analysis (ICA), outlined in Supplementary Note 4)^40^ into the underlying PSI and PSII components. Following this deconvolution, the obtained average PSI and PSII lifetimes were plotted as a function of illumination time and directly compared with the average lifetimes obtained for fully dark-adapted leaves. The results of this analysis are summarized in Fig. 1.

For PSI and PSII, the average ${F}_{\rm{m}}$ lifetimes in fully dark-adapted leaves are (79 ± 3) ps and (1756 ± 25) ps, respectively (magenta square and cyan circle, respectively; Fig. 1a). These average lifetimes were fully consistent with those obtained for the reference ${F}_{\rm{m}}$ measurements performed on 3-(3,4-dichlorophenyl)-1,1-dimethylurea (DCMU)-infiltrated leaves ($\left\langle {\tau }_{\mathrm{DCMU}}^{\mathrm{PSI}}\right\rangle$=(76±1) and $\left\langle {\tau }_{\mathrm{DCMU}}^{\mathrm{PSII}}\right\rangle =(1,753\pm 9)\,\mathrm{ps}$, respectively; Supplementary Fig. 3a). Upon illumination, the average lifetimes observed for PSI and PSII show significantly different behaviours. For PSI, the value of its average lifetime is unaffected by the illumination and remains at ~79 ps during the entire illumination period (Fig. 1a, dashed magenta line). By contrast, the average lifetime of PSII is strongly affected by illumination, decreasing from 1,756 ps to a steady-state value of ~560 ps during the illumination period (Fig. 1a, solid cyan line). These differences in the changes in the average lifetimes of PSII and PSI upon illumination are consistent with the fact that only PSII exhibits NPQ. Finally, by applying the Stern–Volmer equation, these average PSII lifetimes can be used to calculate the ${\rm{NPQ}}^{\rm{Closed}}$ induction curve (Fig. 1b, green diamonds). This ${\rm{NPQ}}^{\rm{Closed}}$ induction curve follows a simple monotonically increasing profile, increasing to a steady-state value of (2.05 ± 0.07) during the induction period. This induction curve illustrates that in these experiments the actinic light source leads to a significant degree of NPQ and that the 20 min of induction is sufficient for the NPQ to reach a steady-state value.

### Ultrafast fluorescence with fully closed ^PSII^RCs (${\boldsymbol{F}}_{\mathbf{m}}$ and ${\boldsymbol{F}}_{\mathbf{m}}^{\prime}$)

**Fig. 2:**
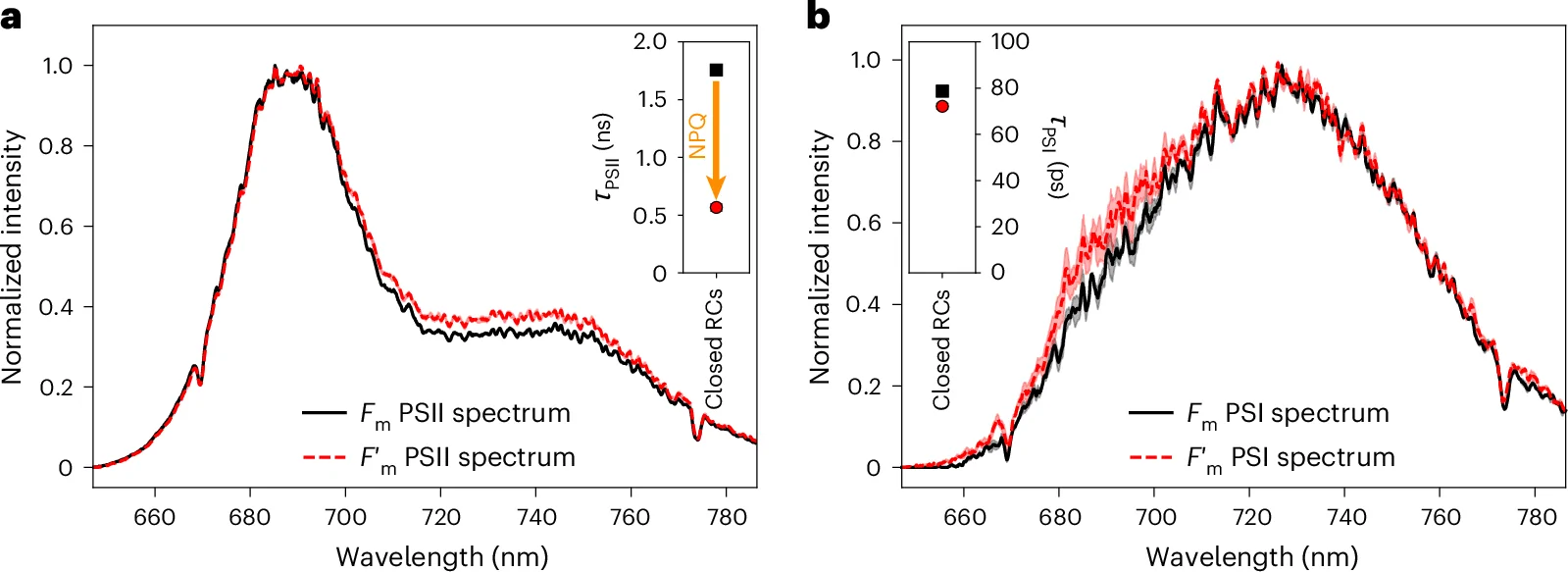
NPQ-induced changes in ultrafast spectra and lifetimes without photochemistry. **a**,**b**, Comparison between the ${F}_{\rm{m}}$ (solid line) and ${F}_{{\rm{m}}}^{{\prime} }$ (dashed line) average normalized spectra (n=3) and average lifetimes (insert; ${{n}}=3$, where the error bars show the associated standard error) of PSII (**a**) and PSI (**b**) in wt *A. thaliana*. Shaded areas show the associated spectral errors.

The NPQ induction measurements performed on wt *A. thaliana* leaves with fully closed ^PSII^RCs (spectra shown in Supplementary Fig. 5) were deconvoluted using spectral ICA^40^, and average normalized spectra and average lifetimes were calculated for both PSII and PSI in the ${F}_{\rm{m}}$ (fully closed ^PSII^RCs without NPQ) and ${F}_{{\rm{m}}}^{{\prime} }$ (fully closed ^PSII^RCs with NPQ) states. The results of this analysis are summarized in Fig. 2.

Both the spectra obtained for PSII and PSI are broadly similar to those seen in previous ultrafast fluorescence measurements^31^. In both the ${F}_{{\rm{m}}}$ and ${F}_{{\rm{m}}}^{{\prime} }$ states the PSII spectrum is noted to have a peak in its fluorescence located at ~685 nm and a distinct fluorescence shoulder centred at ~725 nm. While these spectra are very similar, a direct comparison of their normalized line shapes reveals that there is a small relative increase in the intensity of the shoulder in the ${F}_{{\rm{m}}}^{{\prime} }$ state. This change in the PSII spectrum is comparable to the results of a previous ultrafast fluorescence study^31^. The average PSII lifetimes (Fig. 2a, insert) for the ${F}_{\rm{m}}$ (black square) and ${F}_{{\rm{m}}}^{{\prime} }$ (red circle) states were found to be (1,756 ± 25) ps and (567 ± 31) ps, respectively. This shorter PSII lifetime in the ${F}_{{\rm{m}}}^{{\prime} }$ state reflects the presence of NPQ in this state. Using the Stern–Volmer equation, it is found that this decrease in the PSII lifetime corresponds with an ${\rm{NPQ}}^{\rm{Closed}}$ value of (2.1 ± 0.1). Both of these results are consistent with the results of the series of low-SNR spectra measured during the NPQ induction period (Fig. 1). The PSI spectral line shapes in the ${F}_{\rm{m}}$ and ${F}_{{\rm{m}}}^{{\prime} }$ states are almost identical (within error; Fig. 2b). Specifically, in both of these states, the PSI spectrum has an emission maximum located at ~730 nm, and it is distinctly asymmetric, exhibiting a shoulder towards shorter wavelengths (Fig. 2b). The PSI lifetimes (Fig. 2b, insert) for the ${F}_{\rm{m}}$ (black square) and ${F}_{{\rm{m}}}^{{\prime} }$ (red circle) states were found to be (79 ± 3) ps and (72 ± 1) ps, respectively. These lifetimes are within 7 ps of each other, and this difference is comparable in size to the 4.5 ps temporal resolution of the measurement. Although it is thought that the small difference between the ${F}_{\rm{m}}$ and ${F}_{{\rm{m}}}^{{\prime} }$ PSI lifetimes is due to the temporal resolution of the measurement, it cannot be excluded that there is a little bit of residual mixing between the PSI and PSII components in the ${F}_{\rm{m}}$ deconvolution (Supplementary Note 4). Again, this is broadly consistent with the results of the series of low-SNR spectra obtained during the initial induction period (Fig. 1a).

### Ultrafast fluorescence with fully open ^PSII^RCs (${\boldsymbol{F}}_{\mathbf{o}}$ and ${\boldsymbol{F}}_{\mathbf{o}}^{\prime}$)

**Fig. 3:**
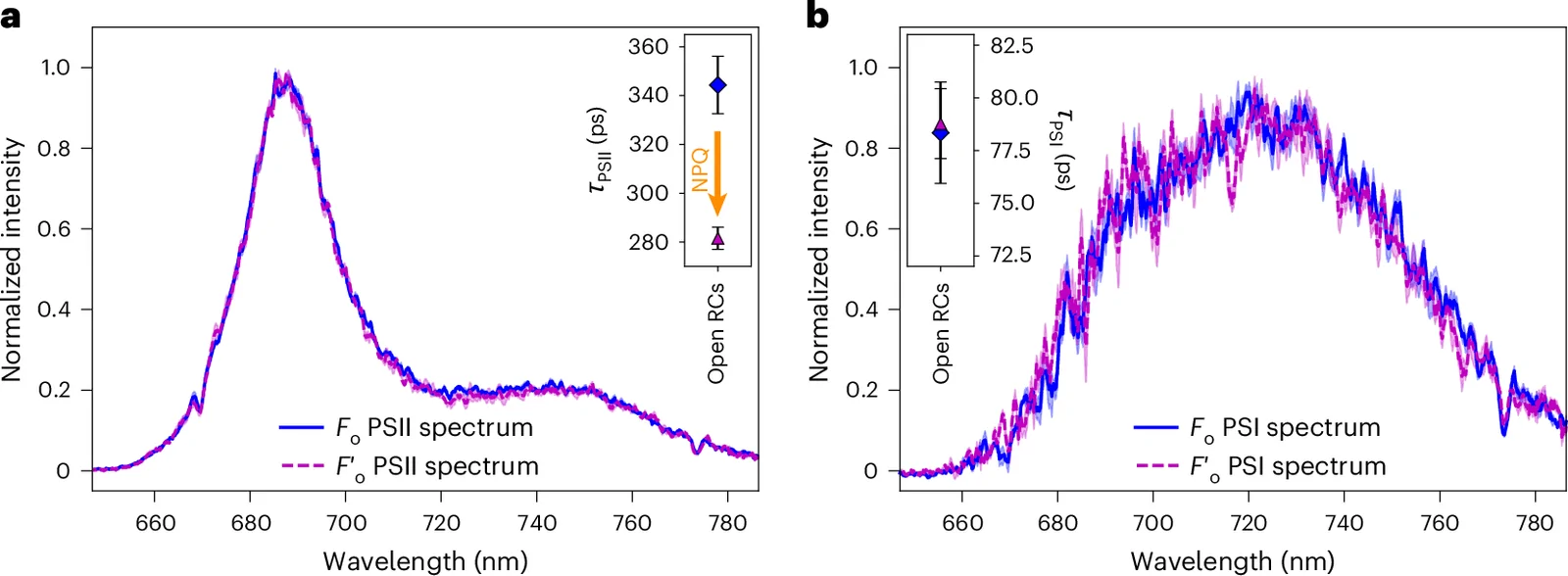
NPQ-induced changes in ultrafast spectra and lifetimes with photochemistry. **a**,**b**, Comparison between the ${F}_{\rm{o}}$ (solid line) and ${F}_{{\rm{o}}}^{{\prime} }$ (dashed line) average normalized spectra (n=3) and average lifetimes (insert; n=3, where the error bars show the associated standard error) of PSII (**a**) and PSI (**b**) in wt *A. thaliana*. Shaded areas show the associated spectral errors.

The average spectra and lifetimes of PSII and PSI in the ${F}_{\rm{o}}$ (fully open ^PSII^RCs without NPQ) and ${F}_{{\rm{o}}}^{{\prime} }$ (fully open ^PSII^RCs with NPQ) states were deconvoluted from the ultrafast measurements (shown in Supplementary Fig. 6) using spectral ICA. The results of this analysis are summarized in Fig. 3.

The normalized spectral line shapes of both PSII (Fig. 3a) and PSI (Fig. 3b) are almost identical in the ${F}_{\rm{o}}$ (fully open ^PSII^RCs without NPQ) and ${F}_{{\rm{o}}}^{{\prime} }$ (fully open ^PSII^RCs with NPQ) states. This indicates that, in the presence of fully open ^PSII^RCs, there are no spectral changes upon NPQ induction, and this is consistent with a previous ultrafast fluorescence study on spinach^31^.

While the PSII and PSI spectral line shapes are not altered by NPQ induction, comparing the average ${F}_{\rm{o}}$ and ${F}_{\rm{m}}$ spectra reveals that closing the ^PSII^RCs seems to lead to an increase in the intensity of the ~725-nm fluorescence shoulder in the PSII spectrum (cf. Figs. 2a and 3a). The average PSII lifetimes (Fig. 3a, insert) for the ${F}_{\rm{o}}$ (blue diamond) and ${F}_{{\rm{o}}}^{{\prime} }$ (magenta triangle) states were found to be (344 ± 12) ps and (281 ± 5) ps, respectively. Applying the ${F}_{{\rm{o}}}^{{\prime} }$ version of the Stern–Volmer equation yields a ${\rm{NPQ}}^{\rm{Open}}$ value of (1.1 ± 0.1). Considering that NPQ induction here is identical to the ${F}_{\rm{m}}$ measurements, this reveals a significant discrepancy between ${\mathrm{NPQ}}^{\mathrm{Open}}$ (${k}_{\mathrm{NPQ}}^{\mathrm{Open}}$) and ${\mathrm{NPQ}}^{\mathrm{Closed}}$ (${k}_{\mathrm{NPQ}}^{\mathrm{Closed}}$; ${\mathrm{NPQ}}^{\mathrm{Closed}}$ = (2.1 ± 0.1); see above and Supplementary Note 2). The average PSI lifetimes (Fig. 3b, insert) for the ${F}_{\rm{o}}$ (blue diamond) and ${F}_{{\rm{o}}}^{{\prime} }$ (magenta triangle) states were found to be (78 ± 2) ps and (79 ± 2) ps, respectively. Again, the similarity of these PSI lifetimes is consistent with the results obtained from the series of low-SNR spectra obtained during the initial induction period (Fig. 1a).

### Steady-state fluorescence

**Fig. 4:**
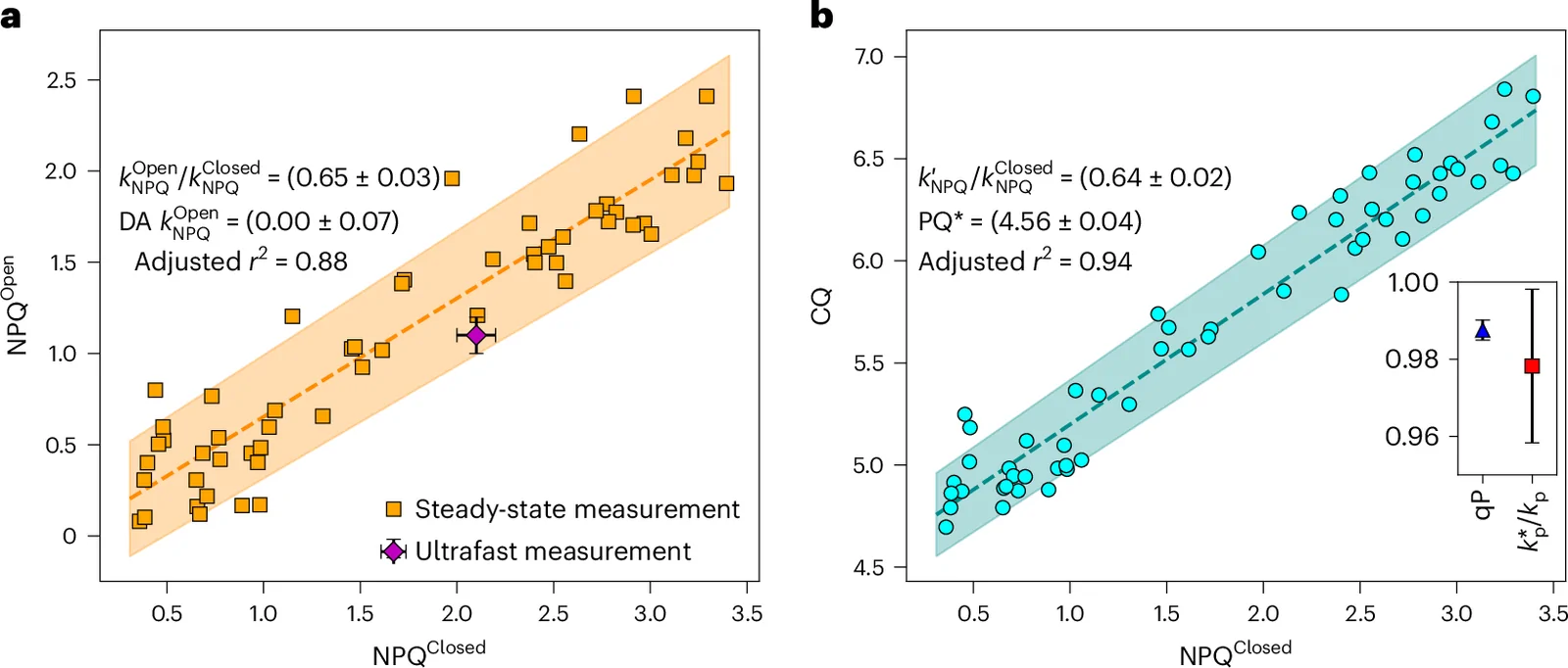
Non-photochemical quenching in the absence and presence of photochemistry in wt *A. thaliana*. **a**,**b**, Fitted correlation plots of ${\rm{NPQ}}^{\rm{Open}}$ versus ${\mathrm{NPQ}}^{\mathrm{Closed}}$ (including the results of the ultrafast fluorescence measurements (magenta diamond), where the error bars show the associated standard error) (**a**) and $\rm{CQ}$ versus ${\mathrm{NPQ}}^{\mathrm{Closed}}$ (**b**), with an insert comparing the average values of qP and $k_{\rm{p}}^*/k_{\rm{p}}$ (where $\mathrm{CQ}=\left({F}_{{\rm{m}}}-{F}^{^{\prime} }\right)/{F}^{{\prime} }={\mathrm{PQ}}^{* }+{\mathrm{mNPQ}}^{\mathrm{Closed}}$ (Supplementary Note 3.3) is the sum of the photochemical and non-photochemical quenching, ${\mathrm{NPQ}}^{\mathrm{Closed}}={k}_{\mathrm{NPQ}}^{\mathrm{Closed}}/\left({k}_{{\rm{f}}}+{k}_{{\rm{d}}}\right)$ is the value of NPQ when the Q_A_ pool is fully reduced (fully closed ^PSII^RCs), qP is a proxy for the portion of open ^PSII^RCs (${\rm{qP}}=1\Rightarrow$ fully open ^PSII^RCs), $k_{\rm{p}}^*$ is the apparent rate of photochemical quenching when the Q_A_ pool is partially reduced (^PSII^RCs are partially open), ${k}_{{\rm{p}}}$ is the rate of photochemical quenching when the Q_A_ pool is fully oxidized (all ^PSII^RCs are open), and ${\mathrm{PQ}}^{* }={k}_{{\rm{p}}}^{* }/\left({k}_{{\rm{f}}}+{k}_{{\rm{d}}}\right)$ is the photochemical quenching when the Q_A_ pool is partially reduced (^PSII^RCs are partially open)), for wt *A. thaliana* (*n* = 8) (calculated with yields corrected for the contribution of PSI; Supplementary Note 1). All of the values were calculated at the end of each 10-min illumination step in the light response curve. All plants were dark-adapted overnight before measurement and illuminated with red actinic light. Error bars show the associated standard errors. DA, dark-adapted.

To further explore the NPQ discrepancy seen in the ultrafast measurements, the fluorescence yield was measured using a pulse-amplitude-modulated (PAM) fluorometer. The fluorescence lifetime and yield are linearly proportional, with the yield being the lifetime multiplied by the rate constant for fluorescence emission (${k}_{{\rm{f}}}$)^41^. Therefore, any discrepancies seen in the ultrafast measurements should also be present in the steady-state yield data. The fluorescence yield measured in PAM fluorometry is known to contain a contribution from PSI, and so to directly compare these data with the lifetimes observed in the ultrafast measurements, the contribution of PSI must be taken into account (Supplementary Note 1). From the results of the ultrafast measurements, it can be concluded that during NPQ induction the PSI fluorescence yield contribution is constant because both the PSI spectra and lifetime are constant (Figs. 1–3). This contribution was calculated from the ultrafast spectrum obtained under ${F}_{\rm{m}}$ conditions (Supplementary Note 1 and Supplementary Fig. 3), and the PAM light response curves were corrected by subtracting this constant contribution. Following this, ${\rm{NPQ}}^{\rm{Closed}}$ and ${\mathrm{NPQ}}^{\mathrm{Open}}$ were calculated following each illumination step in the light response curve. The correlation between ${\mathrm{NPQ}}^{\mathrm{Open}}$ and ${\mathrm{NPQ}}^{\mathrm{Closed}}$ is summarized in Fig. 4.

${\rm{NPQ}}^{\rm{Open}}$ was found to be strongly positively correlated to ${\mathrm{NPQ}}^{\mathrm{Closed}}$ (adjusted *r*^2^ = 0.88 (Fig. 4a), yielding residuals randomly distributed around zero; Supplementary Fig. 7a). A linear fit of this correlation yields a gradient of (0.65 ± 0.03) and an intercept of (0.00 ± 0.07). The fact that this correlation has an intercept at the origin shows that under dark adaptation both ${\rm{NPQ}}^{\rm{Open}}$ and ${\mathrm{NPQ}}^{\mathrm{Closed}}$ are zero, fully consistent with the idea that dark-adapted plants should not exhibit any NPQ. However, the fact that the gradient is not 1 reveals a distinct discrepancy between the NPQ calculated for closed and open ^PSII^RCs. Specifically, this gradient shows that the value of ${\rm{NPQ}}^{\rm{Open}}$ is always smaller than ${\mathrm{NPQ}}^{\mathrm{Closed}}$ ($\Rightarrow {k}_{\mathrm{NPQ}}^{\mathrm{Open}}$ < ${k}_{\mathrm{NPQ}}^{\mathrm{Closed}}$). This correlation also appears to be consistent with the NPQ values for open and closed ^PSII^RCs obtained from the ultrafast fluorescence measurements (Fig. 4a, magenta diamond).

The values of ${\rm{NPQ}}^{\rm{Open}}$ obtained from the fluorescence yield and the ultrafast measurements rely on accurate measurements of ${F}_{{\rm{o}}}^{{\prime} }$ or $\langle {\tau }_{{F}_{{\rm{o}}}^{{\prime} }}^{\mathrm{PSII}}\rangle$. These parameters can only be accurately determined if the ^PSII^RCs are fully reopened (for example, ${\rm{qP}}=1$) immediately following illumination, and before any NPQ relaxation occurs. Far-red light is often used to ensure that as many of the ^PSII^RCs are reopened as quickly as possible following illumination. However, from the PAM traces it is clear that, even with the application of far-red light, it takes several seconds to reach the ${F}_{\rm{o}}^{{\prime} }$ state. At this time, it is possible that there is a small degree of relaxation of the fastest NPQ processes. Combined, this means that it is likely that only approximate values of ${F}_{\rm{o}}^{{\prime} }$ or $\langle {\tau }_{{F}_{{\rm{o}}}^{{\prime} }}^{\mathrm{PSII}}\rangle$ are measured. This might lead to a lower-than-expected value of ${\rm{NPQ}}^{\rm{Open}}$. To further probe the observed discrepancy between ${\mathrm{NPQ}}^{\mathrm{Open}}$ and ${\mathrm{NPQ}}^{\mathrm{Closed}}$, the correlation between ${\rm{CQ}}$ and ${\rm{NPQ}}^{\rm{Closed}}$ was explored (Fig. 4b). ${\rm{CQ}}$ is found to also be strongly positively correlated with ${\mathrm{NPQ}}^{\mathrm{Closed}}$ (adjusted *r*^2^ = 0.94; Fig. 4b, yielding residuals randomly distributed around zero, Supplementary Fig. 7b, yielding a gradient of (0.64 ± 0.02) (${k}_{\mathrm{NPQ}}^{{\prime} }/{k}_{\mathrm{NPQ}}^{\mathrm{Closed}}$ in Fig. 4b) and an intercept of (4.56 ± 0.04) (${{\rm{PQ}}}^{* }$ in Fig. 4b). $\left({F}_{\rm{m}}-{F}^{{\prime} }\right)/{F}^{{\prime} }$ is a parameter that describes the combined photochemical (${\mathrm{PQ}}^{* }={k}_{{\rm{p}}}^{*}/\left({k}_{{\rm{f}}}+{k}_{{\rm{d}}}\right)$) and non-photochemical (${\mathrm{NPQ}}^{{\prime} }={k}_{\mathrm{NPQ}}^{{\prime} }/\left({k}_{{\rm{f}}}+{k}_{{\rm{d}}}\right)$) quenching. Here, this parameter and the corresponding value of ${\mathrm{NPQ}}^{\mathrm{Closed}}$ were calculated using the values of ${F}^{{\prime} }$ and ${F}_{{\rm{m}}}^{{\prime} }$ obtained from the saturating pulse applied following the determination of ${F}_{\rm{o}}^{{\prime} }$ (Supplementary Fig. 2b). Because these pulses are obtained for similar qP values and the overall size of the ${\rm{PQ}}^{* }$ is linearly proportional to qP, this means that ${\rm{CQ}}$ should be linearly correlated with ${\rm{NPQ}}^{\rm{Closed}}$, fully consistent with the experimental results (Fig. 4b). This linear proportionality between ${\rm{PQ}}^{* }$ and qP is further illustrated by the fact that ${\rm{qP}}\cong {k}_{\rm{p}}^{* }/{k}_{\rm{p}}(={{\rm{PQ}}}^{* }/{\rm{PQ}})$ (Fig. 4b, insert; outlined in Supplementary Note 3.8). The gradient of this correlation describes the ratio between ${{\rm{NPQ}}}^{{\prime} }$ and ${\mathrm{NPQ}}^{\mathrm{Closed}}$. Because the values of ${F}^{{\prime} }$ were obtained for leaves with ${\rm{qP}} \approx 1$; (or qL ≈ 1; insert, Fig. 4b, insert), this means that the combined quenching was calculated in the presence of almost fully open RCs and so ${\rm{NPQ}}^{{\prime} }\approx {\rm{NPQ}}^{\rm{Open}}$. In addition, the ${\rm{CQ}}_{\rm{r}}$ versus ${\rm{NPQ}}_{\rm{r}}^{\rm{Closed}}$ correlation, obtained during the post-illumination recovery following the light response curve, also yielded a gradient of (0.7 ± 0.05) (Supplementary Fig. 8). This again shows that there is a discrepancy between the values of ${\rm{NPQ}}^{\rm{Open}}$ and ${\mathrm{NPQ}}^{\mathrm{Closed}}$, and all of the correlations show that ${\mathrm{NPQ}}^{\mathrm{Open}}=0.65{\mathrm{NPQ}}^{\mathrm{Closed}}$ ($\Rightarrow {k}_{\mathrm{NPQ}}^{\mathrm{Open}}=0.65{k}_{\mathrm{NPQ}}^{\mathrm{Closed}}$). Furthermore, because ${F}^{{\prime} }$ is the fluorescence yield obtained just before the saturating pulse, this means that here the value of ${\rm{NPQ}}^{\rm{Open}}$ is obtained first and then the ^PSII^RCs are closed to obtain the corresponding ${\mathrm{NPQ}}^{\mathrm{Closed}}$ value, allowing these values to be obtained within a second of each other.

**Fig. 5:**
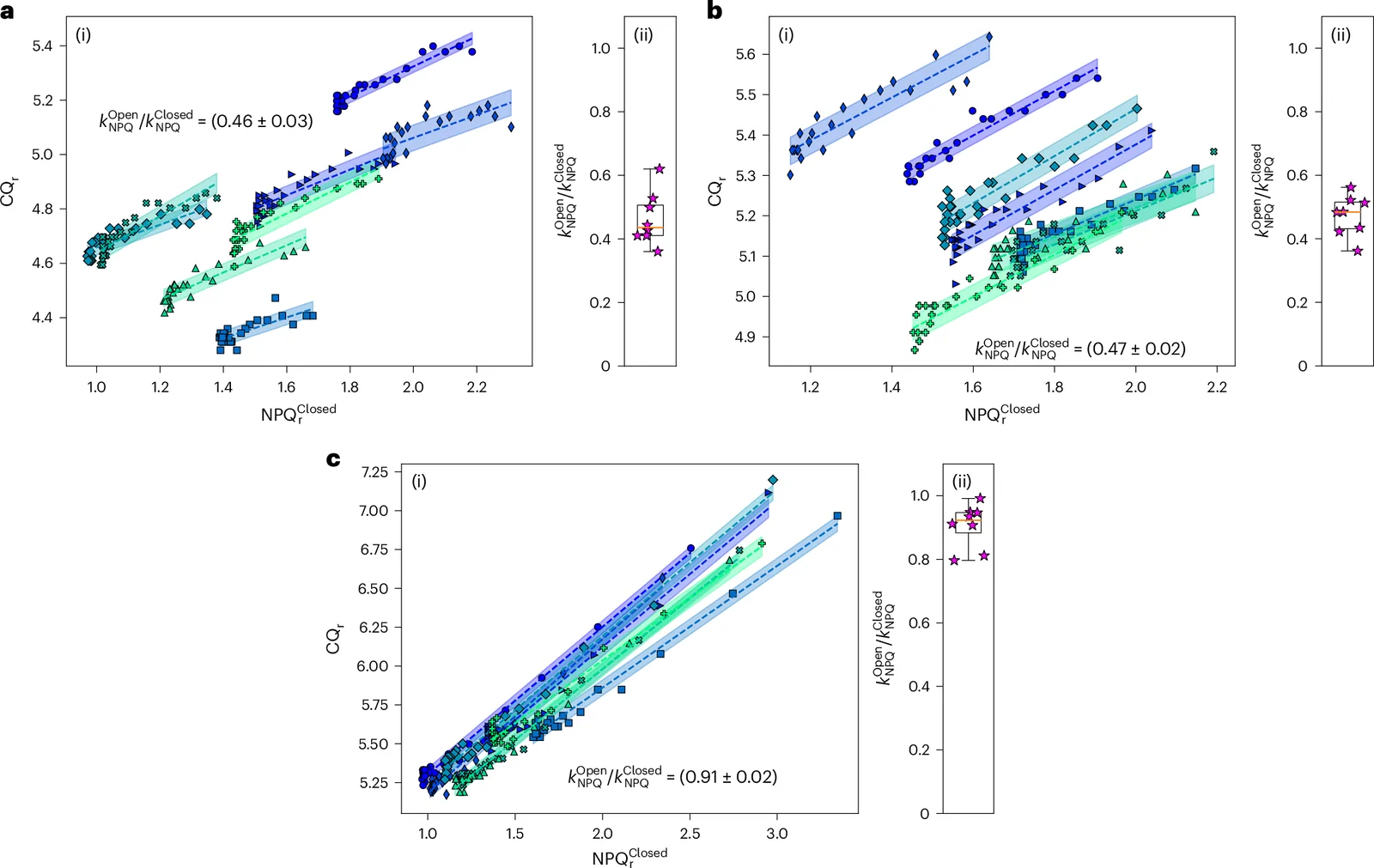
Non-photochemical quenching in the absence of and combined quenching in *npq4*, *npq1* and *L17A. thaliana*. **a**–**c**, Fitted correlation plot of ${\rm{CQ}}_{\rm{r}}$ versus ${\rm{NPQ}}_{\rm{r}}^{\rm{Closed}}$ for *npq4* (**a**), *npq1*(**b**) and *L17* (**c**) *A. thaliana* (*n* = 8). All values were calculated at the end of the light response curve during the post-illumination recovery period. All plants were dark-adapted overnight before measurement and illuminated with red actinic light (calculated with yields corrected for the contribution of PSI). The whiskers on all of the boxplots represent the maximum and minimum of the obtained ${k}_{\rm{NPQ}}^{\rm{Open}}/{k}_{\rm{NPQ}}^{\rm{Closed}}$ values, whereas the bottom, middle and top of the box represent the 25th, 50th and 75th percentiles of these values, respectively.

To understand the effect(s) of PsbS and Zx on the discrepancy between ${\rm{NPQ}}^{\rm{Open}}$ and ${\mathrm{NPQ}}^{\mathrm{Closed}}$, the ${\rm{CQ}}_{\rm{r}}$ versus ${\rm{NPQ}}_{\rm{r}}^{\rm{Closed}}$ correlations of *npq4*, *npq1* and *L17 A. thaliana* were explored. The correlations for these mutants are summarized in Fig. 5 (note that the NPQ values of these mutants obtained during the post-illumination recovery period cannot be directly compared with those presented for wt (Fig. 4) as the wt NPQ values were determined at the end of each 10-min illumination step).

Both *npq4* and *npq1* yield a series of parallel but offset ${{\rm{CQ}}}_{\rm{r}}$ versus ${\rm{NPQ}}_{\rm{r}}^{\rm{Closed}}$ correlations (Fig. 5a,b). While offset, the gradients of these correlations are similar, yielding average gradients of (0.46 ± 0.03) and (0.47 ± 0.02) for *npq4* and *npq1*, respectively. These correlations show that ${\mathrm{NPQ}}^{\mathrm{Open}}=0.46{\mathrm{NPQ}}^{\mathrm{Closed}}$ ($\Rightarrow{{k}}^{\mathrm{Open}}_{\mathrm{NPQ}}=0.46{{k}}^{\mathrm{Closed}}_{\mathrm{NPQ}}$) in *npq4* and *npq1*. The differing intercepts seen in the individual correlations for these plants are thought to be caused by the accumulation of differing amounts of qI (Supplementary Note 3.7). This is consistent with the fact these plants show lower amounts of inducible NPQ upon illumination. Finally, the ${{\rm{CQ}}}_{\rm{r}}$ versus ${\mathrm{NPQ}}_{\rm{r}}^{\mathrm{Closed}}$ correlations (Fig. 5c) yield a gradient of (0.91 ± 0.02) for *L17*. These correlations show that ${\mathrm{NPQ}}^{\mathrm{Open}}=0.91{\mathrm{NPQ}}^{\mathrm{Closed}}$ ($\Rightarrow {k}_{\rm{NPQ}}^{\rm{Open}}=0.91{k}_{\rm{NPQ}}^{\rm{Closed}}$) in *L17*.

## Discussion

Fluorescence lifetime and yield measurements were used to determine the value of NPQ in the presence of fully open and closed ^PSII^RCs. All of these measurements were consistent with each other and revealed that ${\mathrm{NPQ}}^{\mathrm{Open}}=0.65{\mathrm{NPQ}}^{\mathrm{Closed}}$ ($\Rightarrow {k}_{\mathrm{NPQ}}^{\mathrm{Open}}=0.65{k}_{\mathrm{NPQ}}^{\mathrm{Closed}}$). In the case of the yield measurements, it was shown that this discrepancy was observed for ^PSII^RCs that transitioned from being closed to being open (Fig. 4a) as well as for ^PSII^RCs that transitioned from being open to being closed (Fig. 4b). In the latter case, it is noted that the value of ${\rm{NPQ}}^{\rm{Closed}}$ (${k}_{\mathrm{NPQ}}^{\mathrm{Closed}}$) was obtained after the value of ${\mathrm{NPQ}}^{\mathrm{Open}}$ (${k}_{\mathrm{NPQ}}^{\mathrm{Open}}$) and that these values were obtained within a second of each other. This demonstrates that the discrepancy seen between these two values cannot be due to NPQ relaxation processes. Because the fluorescence lifetimes were obtained from deconvoluted streak spectra and the contribution of PSI to the fluorescence yield was removed from the PAM data, any effects due to the contributions of PSI to the yield or lifetimes can also not be responsible for the observed discrepancy (Supplementary Fig. 9). In addition, aspects of the experimental design/measurement source were considered as possible sources for this discrepancy, in particular the parameters of the saturating pulses utilized in the PAM measurement and the possibility that not all of the ^PSII^RCs were fully closed in the ultrafast ${F}_{\rm{m}}$ measurements. For the PAM measurements, the saturating nature of the pulses can be assessed by calculating the linear electron transport rate at the end of each intensity step in the light response curve (Supplementary Fig. 10). The electron transport rate is found to increase monotonically towards an asymptote as the light intensity increases, and this profile shows that in these measurements the saturating pulses are always saturating, eliminating incomplete closure as a possible source of the discrepancy. For the ultrafast ${F}_{\rm{m}}$ measurements, the PSII ${F}_{\rm{m}}$ lifetime was very similar to the PSII lifetime obtained for leaves infiltrated with DCMU (an inhibitor that permanently closes ^PSII^RCs) (Supplementary Fig. 3a, insert), showing that the laser power was sufficient to close all the ^PSII^RCs and also eliminating this as a source of the discrepancy.

In addition to the effects mentioned above, there are several other possible physical/biological sources that might lead to the observed discrepancy. These include state transitions^42–44^, chloroplast movement^45–47^, NPQ heterogeneity, qI-like processes and ^PSII^RC-induced NPQ rate switching^31,32^. State transitions lead to the redistribution of antenna proteins between PSI and PSII, whereas chloroplast movements change the optical properties of the leaf^46^. Overall, both processes can lead to illumination-driven changes in the fluorescence yield parameters (${F}_{\rm{m}}^{{\prime} }$, ${F}_{{\rm{o}}}^{{\prime} }$ and ${F}^{{\prime} }$) obtained during and directly following illumination^42,43^. The use of red actinic light for the fluorescence yield experiments eliminates chloroplast movement, eliminating this as a possible source of the discrepancy. However, the potential effects of state transitions were explored using both a series of numerical simulations and measurements on *stn7 A. thaliana* (outlined in Supplementary Note 3.4), based on the Butler model^39,48^, to determine their effects on the parameters calculated from the fluorescence yield. These simulations and measurements (Supplementary Note 3.5) revealed that these processes will not affect the gradients of fluorescence yield correlations (Supplementary Fig. 12a(ii),b(ii)) but will increase the intercepts away from their input values (Supplementary Fig. 12a(iii),b(iii)). This is inconsistent with the experimental results (Fig. 4), and as these processes do not affect the correlation gradients, they cannot explain the observed discrepancy.

The presented measurements on leaves are noted to be the average response of a large ensemble of individual photosystems. Therefore, it is likely that there is a degree of heterogeneity in both the induction of NPQ and the associated NPQ quenching rate. Again, this effect is difficult to probe experimentally, and so additional numerical simulations (Supplementary Note 3.6) were used to explore the effects of NPQ heterogeneity on the fluorescence yield correlations (Fig. 4). For both correlations, increases in the overall NPQ heterogeneity (Supplementary Figs. 14 and 15) lead to an increase of the gradient of the correlations above the input value (gradient >1). In parallel, the intercept of ${\rm{NPQ}}^{\rm{Open}}$ versus ${\rm{NPQ}}^{\rm{Closed}}$ correlation also becomes negative with increasing heterogeneity (Supplementary Fig. 14). Overall, these changes are inconsistent with the experimental results (Fig. 4), which show that both correlations exhibit gradients <1 and non-negative intercepts. This eliminates also NPQ heterogeneity as a possible source of the observed discrepancy. The effect of the accumulation of slowly reversible NPQ via qI-like processes was also explored as a possible explanation for the observed discrepancy. A series of numerical simulations (Supplementary Note 3.7) revealed that any effects of the accumulation of NPQ from such processes did not significantly affect the gradient of the ${{\rm{CQ}}}_{\rm{r}}$ versus ${\rm{NPQ}}_{\rm{r}}^{\rm{Closed}}$ correlations, obtained during the short post-illumination period when the contributions from these processes are thought to be approximately constant. Because gradients of all three of these correlations for wt are the same within error (Fig. 4 and Supplementary Fig. 8), this also eliminates the accumulation of slowly reversible NPQ via qI-like processes as a possible explanation.

Finally, ‘instantaneous’ ^PSII^RC-induced NPQ quenching rate-switching was considered as a possible source of the observed discrepancy between ${\rm{NPQ}}^{\rm{Open}}$ and ${\mathrm{NPQ}}^{\mathrm{Closed}}$. First proposed to explain differences seen in the $F_{\rm{o}}$ and ${F}_{{\rm{m}}}$ ultrafast fluorescence measurements with and without NPQ, in wt *Spinacia oleracea*, this mechanism indicates that the state of ^PSII^RCs alters the size of the NPQ quenching rate ($\Rightarrow {k}_{\rm{NPQ}}^{\rm{Open}}\ne {k}_{\rm{NPQ}}^{\rm{Closed}}$)^31^. This is fully consistent with the experimentally obtained correlations (Fig. 4), indicating that the discrepancies can only be explained by the presence of ‘instantaneous’ ^PSII^RC-induced NPQ quenching rate-switching (outlined in Supplementary Note 3.8 and Supplementary Fig. 17). This idea is further supported by the intensity increase in the far-red region (~725 nm) of the PSII spectrum (~5–6%; Fig. 2a), upon the induction of NPQ in the ${F}_{\rm{m}}$ state. While the spectral change observed here is smaller, this change in the PSII spectrum was also reported in the previous ultrafast fluorescence study of *S. oleracea*, which proposed the ^PSII^RC-induced quenching rate-switching nature of NPQ^31^. This more complicated nature of NPQ and its quenching rate (${k}_{\rm{NPQ}}$) is not included in the Butler model^48^ and so it has far reaching consequences. Because ${k}_{\mathrm{NPQ}}^{\mathrm{Closed}}\ne {k}_{\mathrm{NPQ}}^{\mathrm{Open}}$, the equations commonly used to calculate several important photosynthetic parameters, such as ${\Phi }_{\rm{PSII}}$ (the quantum yield of PSII), ${\Phi }_{\rm{NPQ}}$ (the quantum yield of NPQ), ${\rm{qP}}$ and $\mathrm{qL}$ (refs. ^35,39^), as well as the Oxborough–Baker equation^49^ for estimating ${F}_{\rm{o}}^{{\prime} }$ (and all expressions derived from this equation)^50,51^, are not fully accurate and need to be adjusted to account for ^PSII^RC-driven changes in the NPQ (outlined for ${\Phi }_{\rm{PSII}}$ in Supplementary Note 5).

A potential explanation for the difference in rates of NPQ might be related to a photoprotection mechanism that was proposed for ^PSII^RCs^52^. In this study, it was found that in closed ^PSII^RCs triplets were formed on the D2 branch β-carotene with a quantum yield between 10% and 20%. These triplets were concluded to originate from triplets created on the nearby D2 chlorophyll (Chl_D2_) via the radical pair dephasing mechanism^53^, mediated by the negatively charged Q_A_ in the closed ^PSII^RC. Because triplets are transferred efficiently from chlorophylls to β-carotenes (with a time constant of 190 ns, much shorter than a typical Chl triplet state lifetime of 1 ms) the quantum yield of Chl_D2_ triplet formation must almost be identical to the observed β-carotene quantum yield (that is, 10–20%). Because these Chl_D2_ triplets are formed from PSII singlet excitations, this is an additional source of quenching in PSII, while the resulting Chl triplets are safely dissipated via the D2 branch β-carotene. This effectively gives closed ^PSII^RCs an additional NPQ channel, explaining the increase of the observed rate of NPQ for closed ^PSII^RCs ($\Rightarrow {k}_{\rm{NPQ}}^{\rm{Open}}$ < ${k}_{\mathrm{NPQ}}^{\mathrm{Closed}}$). It can be roughly estimated (Supplementary Note 6) that the above-mentioned quantum yield of 10–20% corresponds to a rate of quenching of (0.35 ± 0.12) ns^−1^, reflecting an NPQ value of (0.6 ± 0.2), somewhat smaller but close to the difference in the NPQ values found here for open and closed ^PSII^RCs.

Finally, this potential explanation is consistent with the ${k}_{\rm{NPQ}}^{\rm{Open}}/{k}_{\rm{NPQ}}^{\rm{Closed}}$ values seen for *npq4*, *npq1* and *L17*. The additional quenching via the D2 branch β-carotene in closed ^PSII^RCs is presumed to be unaffected by PsbS expression and zeaxanthin levels and suggests that the correlation gradients are given by ${\rm{NPQ}}^{\rm{Open}}/{\rm{NPQ}}^{\rm{Closed}}=\frac{\left({\rm{NPQ}}_{\rm{Zx}}+{\rm{NPQ}}_{\rm{PsbS}}\right)}{({\rm{NPQ}}_{\rm{Zx}}+{\rm{NPQ}}_{\rm{PsbS}}+{\rm{NPQ}}_{\rm{RC}})}$. This implies that the ratio should be higher for *L17* (which has a larger value of $\left({\mathrm{NPQ}}_{\mathrm{Zx}}+{\mathrm{NPQ}}_{\mathrm{PsbS}}\right)$ than wt, increasing ${k}_{\mathrm{NPQ}}^{\mathrm{Open}}/{k}_{\mathrm{NPQ}}^{\mathrm{Closed}}$) and lower in both *npq1* and *npq4* (which have a smaller value of $\left({\mathrm{NPQ}}_{\mathrm{Zx}}+{\mathrm{NPQ}}_{\mathrm{PsbS}}\right)$ than wt, decreasing ${k}_{\mathrm{NPQ}}^{\mathrm{Open}}/{k}_{\mathrm{NPQ}}^{\mathrm{Closed}}$), fully consistent with the experimental results (Fig. 5). Although this potential explanation is fully consistent with the experimental results, the possibility that a non-additive kinetic effect is contributing to the difference between ${\mathrm{NPQ}}^{\mathrm{Closed}}$ and ${\mathrm{NPQ}}^{\mathrm{Open}}$ cannot be excluded.

## Conclusion

Both ultrafast fluorescence lifetime and steady-state fluorescence yield measurements reveal a distinct difference between the value of NPQ obtained for open and closed ^PSII^RCs in wt *A. thaliana*. This difference shows that, in the presence of open ^PSII^RCs, the obtained value of NPQ is ~35% lower than the corresponding NPQ value in the presence of closed ^PSII^RCs. The experimental design coupled with numerical simulations eliminate a wide range of possible physical/biological sources for this difference. Only ‘instantaneous’ ^PSII^RC-induced switching of the rate of NPQ is able to explain the observed experimental results. This means that the NPQ-associated quenching rate (${k}_{\rm{NPQ}}$) ‘instantaneously’ increases when the ^PSII^RCs close. Interestingly, the observed size of this increase in ${k}_{\mathrm{NPQ}}$ (and so NPQ) ^PSII^RC closure is somewhat similar to the size of an additional NPQ channel that we propose in closed ^PSII^RC, based on the formation of Chl_D2_ triplets, which are subsequently quenched by the D2-branch β-carotene^52^. Our results overturn the conventional view that NPQ is an indiscriminate form of photoprotection and indicate that many of the equations commonly used to calculate photosynthetic parameters (for example, ${\Phi }_{\rm{PSII}}$, ${\rm{qP}}$ and ${\Phi }_{\rm{NPQ}}$) need to be adjusted to account for the RC-induced rate-switching nature of NPQ. Moreover, it demonstrates that the potential increase in biomass production by increasing NPQ induction and relaxation rates previously predicted^24^ should be reduced from 30% to 20% (as ${\mathrm{NPQ}}^{\mathrm{Open}}={0.65\mathrm{NPQ}}^{\mathrm{Closed}}$).

## Methods

### Materials

All *A. thaliana* plants were grown in a Hettich ESP PRC 1200 WL growth cabinet (Hettich; https://www.hettichbenelux.com). The cabinet was set to grow plants in short-day conditions (8 h of light and 16 h of darkness) at an actinic light intensity of 125 µmol m^−^^2 ^s^−1^ (see spectrum in Supplementary Fig. 1a), a day-time temperature of 24 °C, a night-time temperature of 22 °C and a relative humidity of 60%. wt, *stn7* (state transition-lacking), *npq4* (PsbS-lacking), *npq1* (Zx-lacking) and *L17* (PsbS-overexpressing) seeds of *A. thaliana* (columbia-0) were sown on soil and allowed to germinate and grow into seedlings for approximately 2 weeks. After this initial growth period, the seedlings were transferred to separate pots and allowed to grow to maturity for a further 4–5 weeks. DCMU and ethanol were purchased from Sigma-Aldrich and used without any further purification. A 50 mM stock solution of DCMU was prepared in ethanol. This stock solution was then used to prepare a 50 μM aqueous solution of DCMU for the infiltration of the leaves before the ultrafast fluorescence reference measurements.

### Fluorescence measurements

#### Ultrafast fluorescence spectrometer

All ultrafast fluorescence measurements were carried out using a Streak camera (Hamumatsu, https://www.hamamatsu.com) with a Leukos Rock supercontinuum picosecond laser system (Leukos, https://www.leukos-laser.com). For these measurements, the supercontinuum laser pulses were filtered to produce a pulse centred at 440 nm with a full width at half maximum of 10 nm and a repetition rate of 38 MHz. The ultrafast spectrometer geometry utilized in these experiments has been described previously^31,54^. In brief, the filtered pulses were coupled to the optical path of the spectrometer utilizing a single-core fibre optic. The excitation pulse was passed through a neutral density filter wheel, allowing its intensity to be adapted, and focused by an achromatic lens onto a rotating cell (diameter 7 cm)^31^ containing the sample (freshly detached leaves placed into the cell on damp paper to provide the leaves with moisture during the measurement). This lens is also used to collect the emitted fluorescence from the sample. The emitted fluorescence is directed through a dichroic long-pass filter, utilized to remove residual reflections of the excitation pulse, before being focused into an Andor Shamrock 500i (Oxford Instruments Group, https://andor.oxinst.com) spectrograph connected to the streak camera. For NPQ induction measurements with closed RCs (${F}_{\rm{m}}$ and ${F}_{\rm{m}}^{{\prime} }$), the rotational speed of the cell was set to 2 rpm and the intensity of the excitation pulse was set to 5 μW (spot size ~100 μm). An additional 532-nm continuous-wave 1-mW diode laser (spot size on the order of millimetres) was used to ensure that all of the RCs were closed during these measurements. An ${F}_{\rm{m}}$ spectrum was then measured on the dark-adapted leaves using the analogue integration of 1,200 individual spectra with an exposure time of 500 ms, yielding a spectrum with a total integration time of 10 min. After this, the ${F}_{{\rm{m}}}$ spectrum was measured, a blue actinic light source (see spectrum in Supplementary Fig. 1b) was turned on (at 14.1 mW) for 20 min to induce NPQ. During this initial 20-min induction period, a series of spectra (2.5 s exposure time) were recorded every 10 s to track the changes in the ultrafast spectrum during the induction period. Following this induction period, a ${F}_{\rm{m}}^{{\prime} }$ spectrum was measured (again using the analogue integration of 1,200 individual spectra with an integration time of 500 ms to obtain a total exposure time of 10 min). For NPQ induction measurements with open RCs (${F}_{\rm{o}}$ and ${F}_{\rm{o}}^{{\prime} }$), the rotational speed of the cell was set to 800 rpm and intensity of the excitation pulse was set to 250 nW (spot size ~100 μm). An ${F}_{\rm{o}}$ spectrum was then measured on the dark-adapted leaves using the analogue integration of 1,200 individual spectra with an exposure time of 500 ms, yielding a spectrum with a total integration time of 10 min. Following this ${F}_{\rm{o}}$ measurement, the leaves were illuminated with a blue actinic light source (see spectrum in Supplementary Fig. 1b) controlled via a microcontroller (ESP32 – Espressif Systems; https://www.espressif.com). For these measurements, the microcontroller was programmed to illuminate the leaves at 14.1 mW for 20 min, to induce NPQ. After this initial induction period, the light was set to switch off for 10 s and then on again (at 14.1 mW) for 60 s in a cyclical manner allowing a 7-s ${F}_{\rm{o}}^{{\prime} }$ spectrum to be measured every cycle. This illumination cycle was repeated 86 times, allowing an ${F}_{\rm{o}}^{{\prime} }$ spectrum with a total integration time of 10 min to be measured^31^.

Finally, for the reference ${F}_{\rm{m}}$ measurements, the leaves were immersed in a 50 μM DCMU solution for 2 h in the dark. After infiltration, the leaves were transferred to the sample cell and the intensity of the excitation pulse was set to 5 μW (spot size ~100 μm). During these measurements, the sample cell was set to rotate at 3 rpm and translate in the horizontal direction at 1 rpm. These reference measurements were used to verify that the average PSII lifetime corresponds to the ${F}_{\rm{m}}$ state. All measurements were performed in triplicate.

#### Walz mini-PAM-II fluorimeter

All steady-state fluorescence measurements were carried out using a Walz mini-PAM-II fluorimeter (Walz; https://www.walz.com) utilizing the inbuilt red actinic light and far-red light sources (see spectra in Supplementary Fig. 1c,d, respectively). wt, *stn7*, *npq4*, *npq1* and *L17 A. thaliana* leaves were dark-adapted overnight before the experiment. During the measurements, individual leaves (intact and attached to the plant) were held in the leaf clamp, and the measuring light, red actinic light, far-red light and saturating pulses (1 s duration and an intensity of 8,000 µmol m^−^^2 ^s^−1^) were directed onto the clamped leaf via a multicore optical fibre. As before, the emitted chlorophyll fluorescence was measured using the same optical fibre. A saturating pulse was used to obtain a dark-adapted measurement of ${F}_{\rm{m}}$. Following this saturating pulse, a light response curve was performed on the leaf consisting of seven steps, lasting 10 min each. Red actinic light intensities of 25, 64, 123, 281, 621, 811 and 1,135 µmol m^−^^2 ^s^−1^ were utilized for the steps in the light response curve measurements applied to wt plants (Supplementary Fig. 2). Between each of these illumination steps, the actinic light was turned off for 10 s, to allow ${F}_{\rm{o}}^{{\prime} }$ to be determined for each illumination intensity. During the determination of ${F}_{{\rm{o}}}^{{\prime} }$, the leaf was illuminated with the inbuilt far-red light source. Following each of these ${F}_{\rm{o}}^{{\prime} }$ measurements, another saturating pulse was used to obtain ${F}_{\rm{m}}^{{\prime} }$ and ${F}^{{\prime} }$ values. Finally, after the final light intensity, the plants were allowed to undergo a post-illumination recovery for a further 5 min, during which saturating pulses were applied at 30-s intervals, yielding ${F}_{\rm{m}}^{{\prime} }$ and ${F}^{{\prime} }$ values throughout this recovery. These values were corrected for the PSI contribution to the fluorescence yield and for the slight increase in measuring light intensity caused by the inbuilt far-red light source (Supplementary Note 1). Then, these values were used to calculate ${\rm{NPQ}}^{\rm{Closed}}$, ${\mathrm{NPQ}}^{\mathrm{Open}}$ and $\mathrm{CQ}$ following each step in the light response curve, allowing the ${\rm{NPQ}}^{\rm{Open}}$ versus ${\rm{NPQ}}^{\rm{Closed}}$, ${\mathrm{CQ}}$ versus ${\mathrm{NPQ}}^{\mathrm{Closed}}$ and ${\mathrm{CQ}}_{\mathrm{r}}$ versus ${\rm{NPQ}}_{\rm{r}}^{\rm{Closed}}$ (obtained from the post-illumination recovery period) correlations to be explored. This measurement was repeated eight times on randomly selected leaves for each plant.

### Data analysis of ultrafast fluorescence spectra

All of the ultrafast fluorescence spectra were analysed using a custom Python3.9 script. In brief, this script utilizes ICA^40^ applied along the temporal axis to identify the independent spectral components (in this case these arise from PSI and PSII, respectively) contributing to the spectral slice at each recorded timepoint. Because ICA extracts these components using the changes in the contributions of each spectral component over time, these extracted components represent the spectral components associated with the ultrafast fluorescence. These spectral components are then fitted to each of the spectral slices to obtain the associated decay curves. These decays are then fitted using exponential decay functions (convoluted with a Gaussian to account for the instrument response function, including an additional non-negative offset to account for the steady-state background fluorescence due to either the CW laser or the CW laser and actinic light source in the ${F}_{\rm{m}}$ and ${F}_{\rm{m}}^{{\prime} }$ spectra) to obtain average PSII and PSI lifetimes. For the lower-SNR spectra measured during the initial 20-min NPQ induction, this analytical method is altered to allow the ICA to be guided by the results of the analysis of the ${F}_{\rm{m}}$ spectrum. This allowed the average lifetimes of PSII and PSI to be obtained every 10 s during the NPQ induction period. Using these lifetimes, ${\mathrm{NPQ}}_{{\rm\tau }}^{\mathrm{Closed}}$ was determined using the Stern–Volmer equation (${\mathrm{NPQ}}_{\tau }^{\mathrm{Closed}}=\left(\left\langle {\tau }_{{F}_{{\rm{m}}}}^{\mathrm{PSII}}\right\rangle -\left\langle {\tau }_{{F}_{{\rm{m}}}^{^{\prime} }}^{\mathrm{PSII}}\right\rangle \right)/\left\langle {\tau }_{{F}_{{\rm{m}}}^{{\prime} }}^{\mathrm{PSII}}\right\rangle$).

### Reporting summary

Further information on research design is available in the Nature Portfolio Reporting Summary linked to this article.

## Supplementary information

Supplementary Information Supplementary Figs. 1–24, Notes 1–6 and Tables 1 and 2 and discussion and numerical simulations.

Reporting Summary

Peer Review File

## Data Availability

The original contributions presented in the study are available via GitHub at https://github.com/L-Ramakers/Heimdall.

## Code availability

The custom analysis code used in the study is available via GitHub at https://github.com/L-Ramakers/Heimdall.

## References

[1] Blankenship, R. E. *Molecular Mechanisms of Photosynthesis* (Wiley, 2008); 10.1002/9780470758472

[2] Johnson, M. P. Photosynthesis. *Essays Biochem.* **60**, 255–273 (2016). 27784776 10.1042/EBC20160016 PMC5264509

[3] Nelson, N. & Yocum, C. F. Structure and function of photosystems I and II. *Annu. Rev. Plant Biol.*10.1146/annurev.arplant.57.032905.105350 (2006). 10.1146/annurev.arplant.57.032905.105350 16669773

[4] Demmig-Adams, B. & Adams, W. W. The role of xanthophyll cycle carotenoids in the protection of photosynthesis. *Trends Plant Sci.* **1**, 14162–14167 (1996).

[5] De Bianchi, S., Ballottari, M., Dall’Osto, L. & Bassi, R. Regulation of plant light harvesting by thermal dissipation of excess energy. *Biochem. Soc. Trans.*10.1042/BST0380651 (2010). 10.1042/BST0380651 20298238

[6] Horton, P., Ruban, A. V. & Walters, R. G. Regulation of light harvesting in green plants. *Annu. Rev. Plant Physiol. Plant Mol. Biol.* **47**, 655–684 (1996). 15012304 10.1146/annurev.arplant.47.1.655

[7] Ruban, A. V., Johnson, M. P. & Duffy, C. D. P. The photoprotective molecular switch in the photosystem II antenna. *Biochim. Biophys. Acta Bioenerg.*10.1016/j.bbabio.2011.04.007 (2012). 10.1016/j.bbabio.2011.04.007 21569757

[8] van Amerongen, H. & Croce, R. Nonphotochemical quenching in plants: mechanisms and mysteries. *Plant Cell* **37**, koaf240 (2025). 41058045 10.1093/plcell/koaf240 PMC12619064

[9] Goldschmidt-Clermont, M. & Bassi, R. Sharing light between two photosystems: mechanism of state transitions. *Curr. Opin. Plant Biol.*10.1016/j.pbi.2015.04.009 (2015). 10.1016/j.pbi.2015.04.009 26002067

[10] Townsend, A. J. et al. The causes of altered chlorophyll fluorescence quenching induction in the *Arabidopsis* mutant lacking all minor antenna complexes. *Biochim. Biophys. Acta Bioenerg.* **1859**, 666–675 (2018). 29548769 10.1016/j.bbabio.2018.03.005

[11] Li, Z. et al. Lutein accumulation in the absence of zeaxanthin restores nonphotochemical quenching in the *Arabidopsis thaliana* npq1 mutant. *Plant Cell* **21**, 1798–1812 (2009). 19549928 10.1105/tpc.109.066571 PMC2714924

[12] Long, S. P. et al. Into the shadows and back into sunlight: photosynthesis in fluctuating light. *Annu. Rev. Plant Biol.* **73**, 617–648 (2022). 35595290 10.1146/annurev-arplant-070221-024745

[13] Armbruster, U. et al. Regulation and levels of the thylakoid K^+^/H^+^ antiporter KEA3 shape the dynamic response of photosynthesis in fluctuating light. *Plant Cell Physiol.* **57**, 1557–1567 (2016). 27335350 10.1093/pcp/pcw085 PMC4937787

[14] Ruban, A. V. Nonphotochemical chlorophyll fluorescence quenching: mechanism and effectiveness in protecting plants from photodamage. *Plant Physiol.* **170**, 1903–1916 (2016). 26864015 10.1104/pp.15.01935 PMC4825125

[15] Ruban, A. V. The mechanism of nonphotochemical quenching: the end of the ongoing debate. *Plant Physiol.* **181**, 383–384 (2019). 31582470 10.1104/pp.19.00538 PMC6776853

[16] Ruban, A. V. & Wilson, S. The mechanism of non-photochemical quenching in plants: localization and driving forces. *Plant Cell Physiol.* **62**, 1063–1072 (2021). 33351147 10.1093/pcp/pcaa155

[17] D’Haese, D. et al. Non-photochemical quenching kinetics during the dark to light transition in relation to the formation of antheraxanthin and zeaxanthin. *J. Theor. Biol.* **227**, 175–186 (2004). 14990382 10.1016/j.jtbi.2003.10.011

[18] Johnson, M. P., Pérez-Bueno, M. L., Zia, A., Horton, P. & Ruban, A. V. The zeaxanthin-independent and zeaxanthin-dependent qE components of nonphotochemical quenching involve common conformational changes within the photosystem II antenna in *Arabidopsis*. *Plant Physiol.* **149**, 1061–1075 (2009). 19011000 10.1104/pp.108.129957 PMC2633848

[19] Demmig-Adams, B. & Adams, W. W. Xanthophyll cycle and light stress in nature: uniform response to excess direct sunlight among higher plant species. *Planta* **198**, 460–470 (1996).

[20] Li, X. P. et al. Regulation of photosynthetic light harvesting involves intrathylakoid lumen pH sensing by the PsbS protein. *J. Biol. Chem.* **279**, 22866–22874 (2004). 15033974 10.1074/jbc.M402461200

[21] Jahns, P. & Holzwarth, A. R. The role of the xanthophyll cycle and of lutein in photoprotection of photosystem II. *Biochim. Biophys. Acta Bioenerg.* **1817**, 182–193 (2012). 10.1016/j.bbabio.2011.04.012 21565154

[22] Sylak-Glassman, E. J. et al. Distinct roles of the photosystem II protein PsbS and zeaxanthin in the regulation of light harvesting in plants revealed by fluorescence lifetime snapshots. *Proc. Natl Acad. Sci. USA* **111**, 17498–17503 (2014). 25422428 10.1073/pnas.1418317111 PMC4267351

[23] Belgio, E. et al. Economic photoprotection in photosystem II that retains a complete light-harvesting system with slow energy traps. *Nat. Commun.* **5**, 4433 (2014). 25014663 10.1038/ncomms5433

[24] Zhu, X. G., Long, S. P. & Ort, D. R. Improving photosynthetic efficiency for greater yield. *Annu. Rev. Plant Biol.* **61**, 235–261 (2010). 20192734 10.1146/annurev-arplant-042809-112206

[25] Garcia-Molina, A. & Leister, D. Accelerated relaxation of photoprotection impairs biomass accumulation in *Arabidopsis*. *Nat. Plants* **6**, 9–12 (2020). 31907400 10.1038/s41477-019-0572-z

[26] Souza, A. P. et al. Soybean photosynthesis and crop yield are improved by accelerating recovery from photoprotection. *Science* **377**, 851–854 (2022). 35981033 10.1126/science.adc9831

[27] Lehretz, G. G., Schneider, A., Leister, D. & Sonnewald, U. High non-photochemical quenching of VPZ transgenic potato plants limits CO_2_ assimilation under high light conditions and reduces tuber yield under fluctuating light. *J. Integr. Plant Biol.* **64**, 1821–1832 (2022). 35763422 10.1111/jipb.13320

[28] Gotarkar, D. et al. Variation in relaxation of non-photochemical quenching between the founder genotypes of the soybean (*Glycine max*) nested association mapping population. *Plant J.* **121**, e17219 (2025).

[29] Cowling, S. B. et al. Out of Africa: characterizing the natural variation in dynamic photosynthetic traits in a diverse population of African rice (*Oryza glaberrima*). *J. Exp. Bot.* **73**, 3283–3298 (2022). 34657157 10.1093/jxb/erab459 PMC9126740

[30] Kromdijk, J. et al. Improving photosynthesis and crop productivity by accelerating recovery from photoprotection. *Science* **354**, 851–857 (2016). 27856901 10.1126/science.aai8878

[31] Farooq, S. et al. Dynamic feedback of the photosystem II reaction centre on photoprotection in plants. *Nat. Plants* **4**, 225–231 (2018). 29610535 10.1038/s41477-018-0127-8

[32] Van Amerongen, H. & Chmeliov, J. Instantaneous switching between different modes of non-photochemical quenching in plants. Consequences for increasing biomass production. *Biochim. Biophys. Acta Bioenerg.* **1861**, 1–9 (2020). 10.1016/j.bbabio.2019.148119 31734196

[33] Steen, C. J., Morris, J. M., Short, A. H., Niyogi, K. K. & Fleming, G. R. Complex roles of PsbS and xanthophylls in the regulation of nonphotochemical quenching in *Arabidopsis thaliana* under fluctuating light. *J. Phys. Chem. B* **124**, 10311–10325 (2020). 33166148 10.1021/acs.jpcb.0c06265

[34] Amarnath, K., Zaks, J., Park, S. D., Niyogi, K. K. & Fleming, G. R. Fluorescence lifetime snapshots reveal two rapidly reversible mechanisms of photoprotection in live cells of *Chlamydomonas reinhardtii*. *Proc. Natl Acad. Sci. USA* **109**, 8405–8410 (2012). 22586081 10.1073/pnas.1205303109 PMC3365229

[35] Maxwell, K. & Johnson, G. N. Chlorophyll fluorescence—a practical guide. *J. Exp. Bot.* **51**, 659–668 (2000). 10938857 10.1093/jxb/51.345.659

[36] Baker, N. R. Chlorophyll fluorescence: a probe of photosynthesis in vivo. *Annu. Rev. Plant Biol.* **59**, 89–113 (2008). 18444897 10.1146/annurev.arplant.59.032607.092759

[37] Croce, R., van Grondelle, R., van Amerongen, H. & van Stokkum, I. (eds) *Light Harvesting in Photosynthesis* (CRC Press, 2018). 10.1007/s11120-017-0457-9 29043539

[38] Bilger, W. & Bjӧrkman, O. Role of the xanthophyll cycle in photoprotection elucidated by measurements of light-induced absorbance changes, fluorescence and photosynthesis in leaves of *Hedera canariensis*. *Photosynth. Res.* **25**, 173–185 (1990). 24420348 10.1007/BF00033159

[39] Harbinson, J. in *Light Harvesting in Photosynthesis* (eds Croce, R. et al.) 539–573 (CRC Press, 2018).

[40] Hyvärinen, A. & Oja, E. Independent component analysis: algorithms and applications. *Neural Netw.* **13**, 411–430 (2000). 10946390 10.1016/s0893-6080(00)00026-5

[41] Hollas, J. M. *Modern Spectroscopy* (Wiley, 2013).

[42] Vetoshkina, D. V. et al. Dependence of state transitions on illumination time in arabidopsis and barley plants. *Protoplasma*10.1007/s00709-023-01877-z (2023). 10.1007/s00709-023-01877-z 37462717

[43] Papageorgiou, G. C. & Govindjee. Photosystem II fluorescence: slow changes—scaling from the past. *J. Photochem. Photobiol. B* **104**, 258–270 (2011). 21530301 10.1016/j.jphotobiol.2011.03.008

[44] Verhoeven, D., van Amerongen, H. & Wientjes, E. Single chloroplast in folio imaging sheds light on photosystem energy redistribution during state transitions. *Plant Physiol.* **191**, 1186–1198 (2023). 36478277 10.1093/plphys/kiac561 PMC9922397

[45] Baránková, B., Lazár, D. & Nauš, J. Analysis of the effect of chloroplast arrangement on optical properties of green tobacco leaves. *Remote Sens. Environ.* **174**, 181–196 (2016).

[46] Davis, P. A., Caylor, S., Whippo, C. W. & Hangarter, R. P. Changes in leaf optical properties associated with light-dependent chloroplast movements. *Plant Cell Environ.* **34**, 2047–2059 (2011). 21819411 10.1111/j.1365-3040.2011.02402.x

[47] Wilson, S. & Ruban, A. V. Rethinking the influence of chloroplast movements on non-photochemical quenching and photoprotection. *Plant Physiol.* **183**, 1213–1223 (2020). 32404415 10.1104/pp.20.00549 PMC7333707

[48] Butler, W. L. Energy distribution in the photochemical apparatus of photosynthesis. *Ann. Rev. Plant Physiol.* **29**, 345–378 (1978).

[49] Oxborough, K. & Baker, N. R. Resolving chlorophyll a fluorescence images of photosynthetic efficiency into photochemical and non-photochemical components—calculation of qP and without measuring . *Photosynth. Res.* **54**, 135–142 (1997).

[50] Ruban, A. V. & Murchie, E. H. Assessing the photoprotective effectiveness of non-photochemical chlorophyll fluorescence quenching: a new approach. *Biochim. Biophys. Acta Bioenerg.* **1817**, 977–982 (2012). 10.1016/j.bbabio.2012.03.026 22503831

[51] Pfündel, E. E., Klughammer, C., Meister, A. & Cerovic, Z. G. Deriving fluorometer-specific values of relative PSI fluorescence intensity from quenching of F_0_ fluorescence in leaves of *Arabidopsis thaliana* and *Zea mays*. *Photosynth. Res.* **114**, 189–206 (2013). 23196877 10.1007/s11120-012-9788-8

[52] Martínez-Junza, V. et al. A photoprotection mechanism involving the D2 branch in photosystem II cores with closed reaction centers. *Photochem. Photobiol. Sci.* **7**, 1337–1343 (2008). 18958320 10.1039/b809884k

[53] Hoff, A. J., Rademaker, H., van Grondelle, R. & Duysens, L. N. M. On the magnetic field dependence of the yield of the triplet state in reaction centers of photosynthetic bacteria. *Biochim. Biophys. Acta* **460**, 547–554 (1977). 301748 10.1016/0005-2728(77)90094-9

[54] Tian, L., Farooq, S. & Van Amerongen, H. Probing the picosecond kinetics of the photosystem II core complex in vivo. *Phys. Chem. Chem. Phys.* **15**, 3146–3154 (2013). 23340737 10.1039/c3cp43813a

