# Supplementary material for: The state of plant photosystem II reaction centres affects the rate of non-photochemical quenching
Source: Nat Plants. 2026 Aug 14;12(8):1637–46. doi: 10.1038/s41477-026-02357-x (PMC13481247; doi:10.1038/s41477-026-02357-x)
Supplement: Supplementary file 1 [file 41477_2026_2357_MOESM1_ESM.pdf]

nature plants

Supplementary information

https://doi.org/10.1038/s41477-026-02357-x

The state of plant photosystem II reaction centres affects the rate of non-photochemical quenching

In the format provided by the authors and unedited

The State of Plant Photosystem II Reaction Centres affects the rate of Non-Photochemical Quenching

Lennart A. I. Ramakers[1], Yuxi Niu[1], Jeremy Harbinson[1], Arjen Bader[1,2] and Herbert van Amerongen[1,2]*

1 – Laboratory of Biophysics, Wageningen University, Wageningen, The Netherlands

2 – MicroSpectroscopy Research Facility, Wageningen University, Wageningen, The Netherlands

*Corresponding author email: herbert.vanamerongen@wur.nl

Supporting Information:

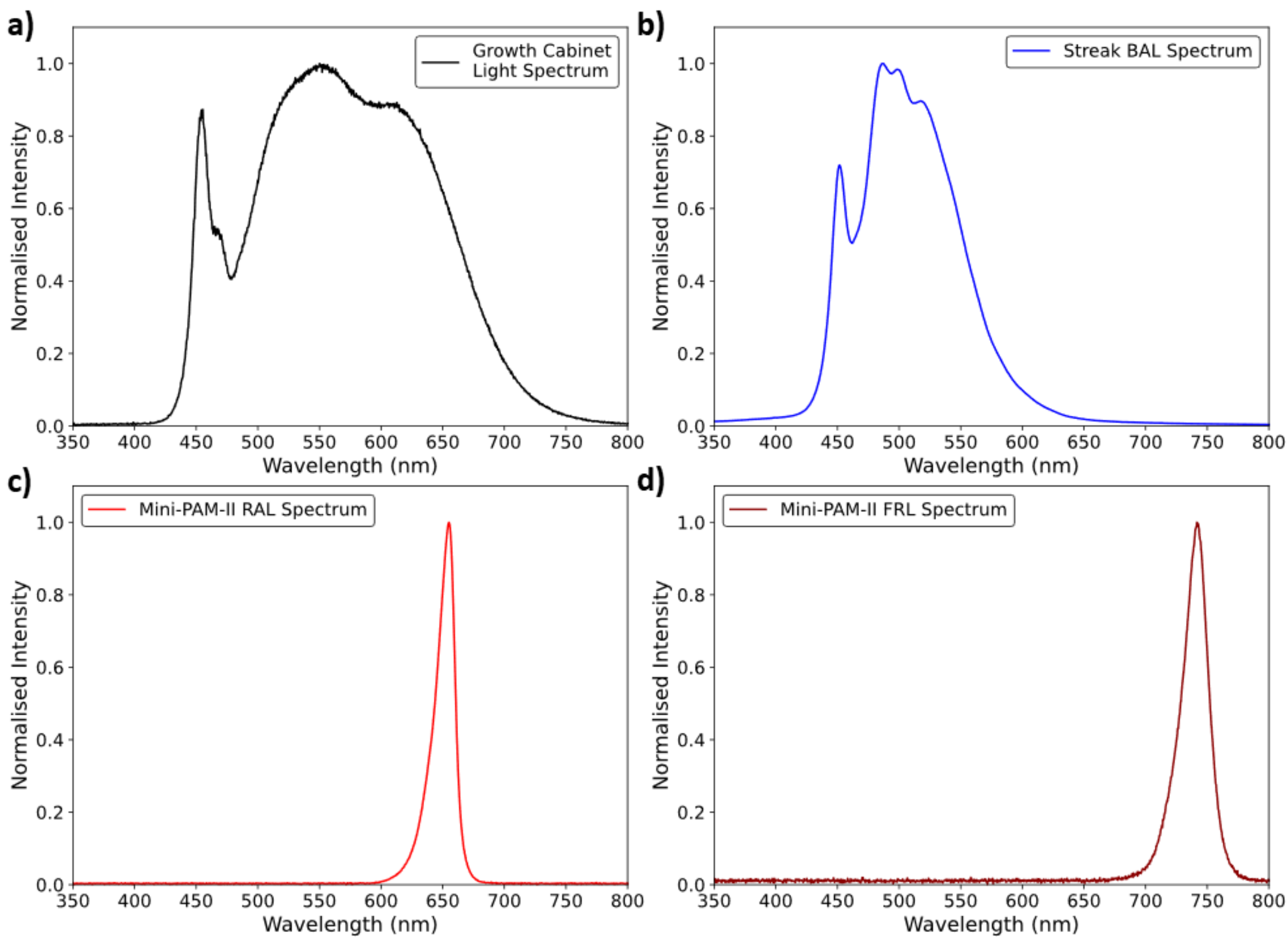

Figure S1: Emission spectra of a) the actinic light utilised by the Hettich ESP PRC 1200 WL growth cabinet, b) the blue actinic light (BAL) source used in the ultrafast fluorescence induction measurements, c) the Walz mini-PAM-II in-built red actinic light (RAL) source and d) the Walz mini-PAM-II in-built far-red light (FRL) source.

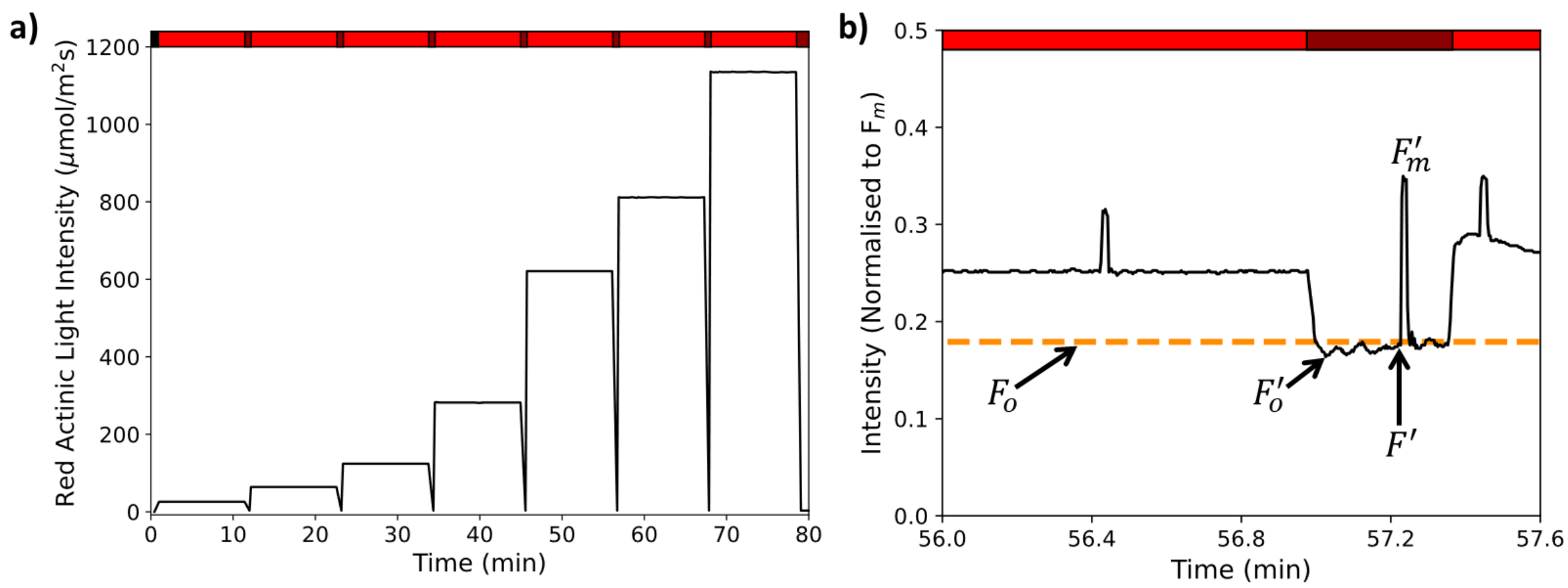

Figure S2: a) The red actinic light intensity profile utilised in the light response curve measurements for wt *A. thaliana* performed on the Walz mini-PAM-II and b) an example of a normalised fluorescence yield trace showing where the values of F_o, F_o', F' and F_m' are determined during the light response curve measurements (for the correlations shown in Fig.4).

Notes S1: Fluorescence Yield Parameter Corrections

S1.1 – Correction for the PSI contribution to the Fluorescence Yield

PSI provides a constant contribution (Fig. 1.(a)) to the overall fluorescence yield measured *via* steady-state techniques such as PAM. To allow the results of these steady state yield measurements and the ultrafast measurements to be directly compared the PSI contribution should be subtracted from the measured fluorescence yield. Here, the size of this correction (as a fraction) is calculated directly using the deconvoluted ultrafast fluorescence measurement performed on wt leaves in the F_m state (with fully closed ^{PSII}RCs). The results of these measurements and calculation are summarised in figure S3.

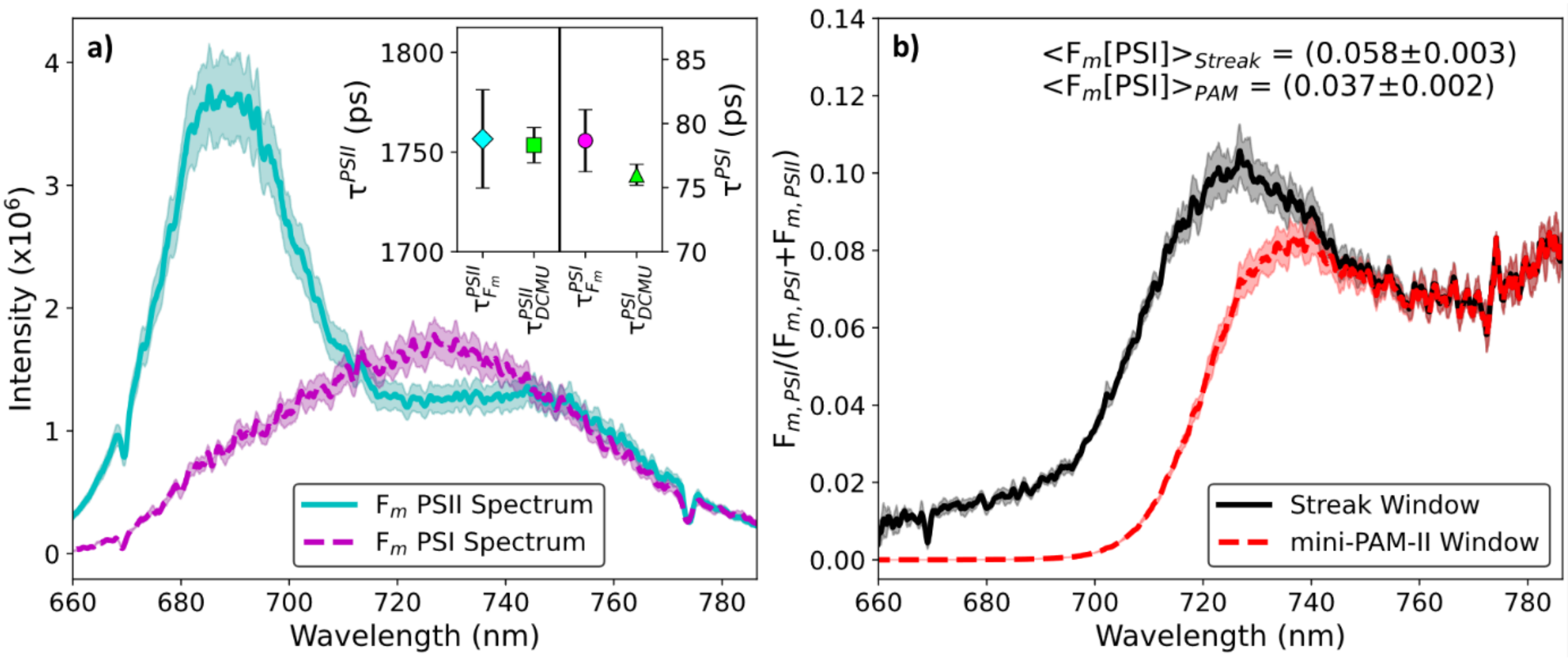

Figure S3: a) The PSII (cyan) and PSI (magenta) F_m spectra, with an insert showing the average lifetimes of PSII and PSI (with a comparison to DCMU-treated leaves), and b) the contribution of PSI to F_m considering the detection window of the streak (black) and mini-PAM-II (red). Shaded areas show the spectral associated standard error and the error bars in the insert show the standard error associated with the average lifetimes.

These measurements show that for the mini-PAM-II the contribution of PSI to F_m is (3.7±0.2)%. In order to account for this PSI contribution and allow for a direct comparison with the ultrafast measurements, the contribution is calculated for each of the PAM measurements and subtracted.

S1.2 – Correction for the far-red illumination

In the PAM measurements we apply far-red light to ensure that F'_o values are accurately determined between each of the illumination steps in the light response curve. Since the far-red light is on during the determination of F'_o but not on during the initial measurement of F_o (to ensure the leaf remains fully dark adapted the measurement of F_o), it is important to check if the far-red light has any effect on the measured fluorescence yield. The spectra of the inbuilt mini-PAM-II measuring light source and far-red light source as well as their effect on the measured fluorescence yield are summarised in figure S4.

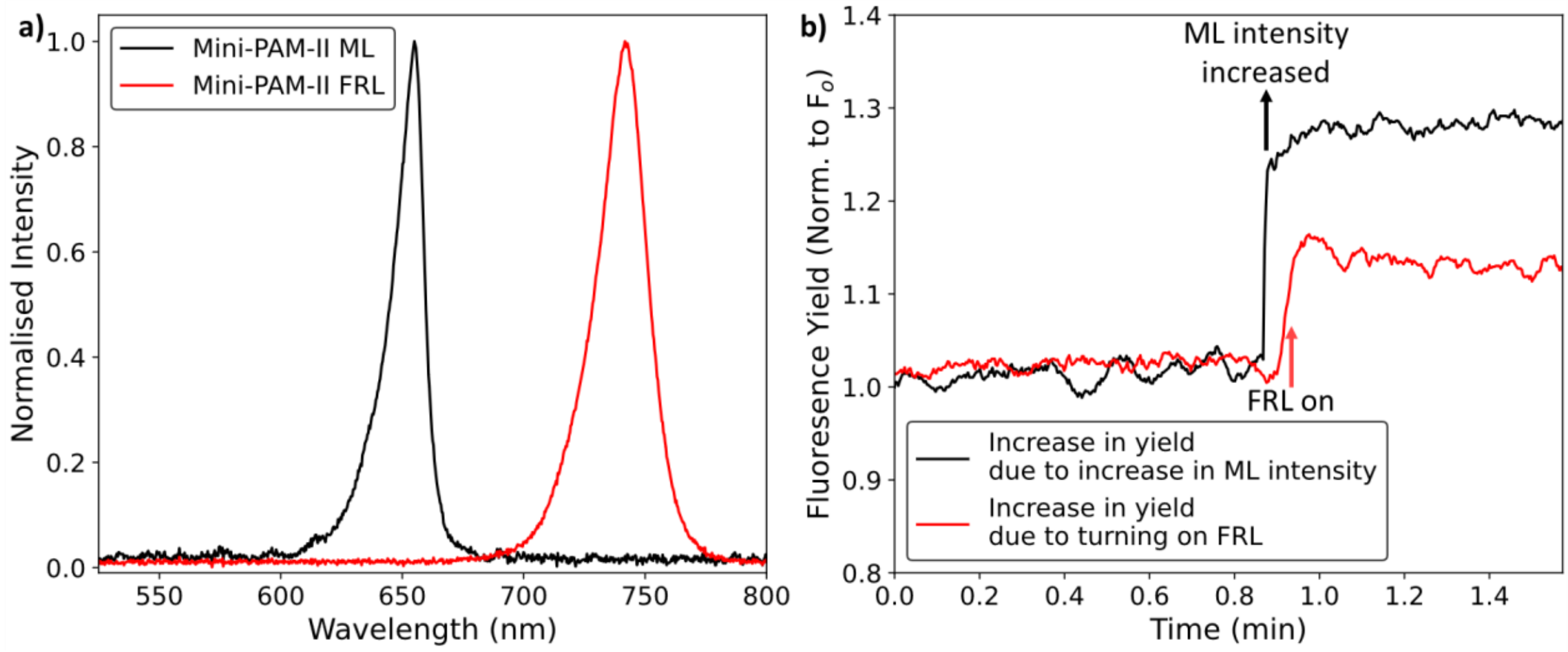

Figure S4: a) Emission spectra of the inbuilt measuring light (black) and the far-red light utilised by the Walz mini-PAM-II and b) the effect of an increase in measuring light intensity (black) or turning on the far-red light source (red).

Both an increase in the intensity of the measuring light or the activation of the far-red light leads to a step-wise increase in the fluorescence yield (~30% upon an increase in the intensity of the measuring light and ~12% upon the activation of the far-red light). This suggests that the far-red

light leads to a small increase in the intensity of the measuring light even though these light sources are found to have well separated emission spectra. However, since the far-red light is required to accurately determine the value of F'$_o$, these values are corrected for the fact that the activation of this light source results in a small increase in the obtained fluorescence yield.

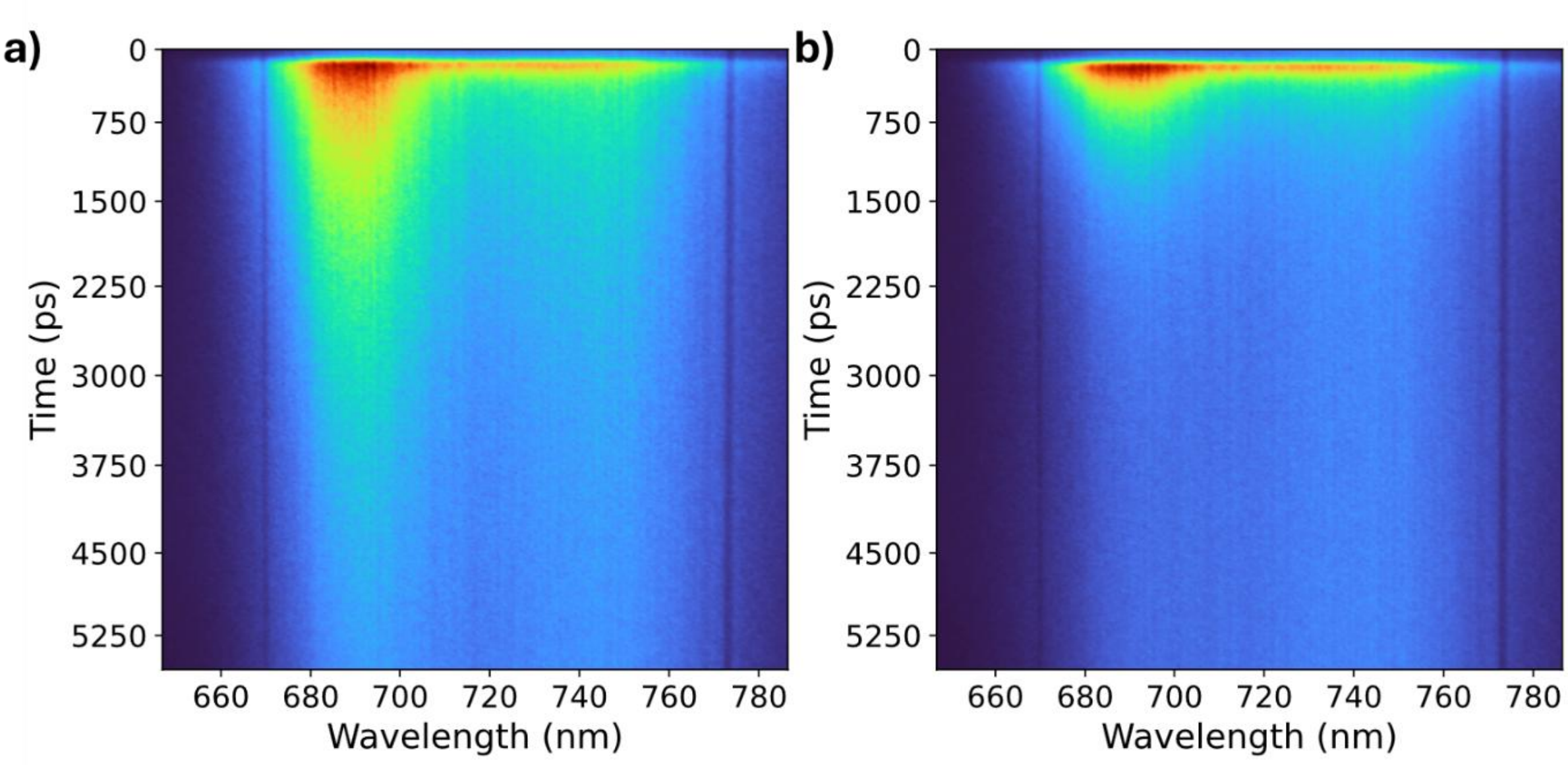

Figure S5: Representative ultrafast fluorescence spectra of wt *A. thaliana* under a) F_m and b) F_m' conditions.

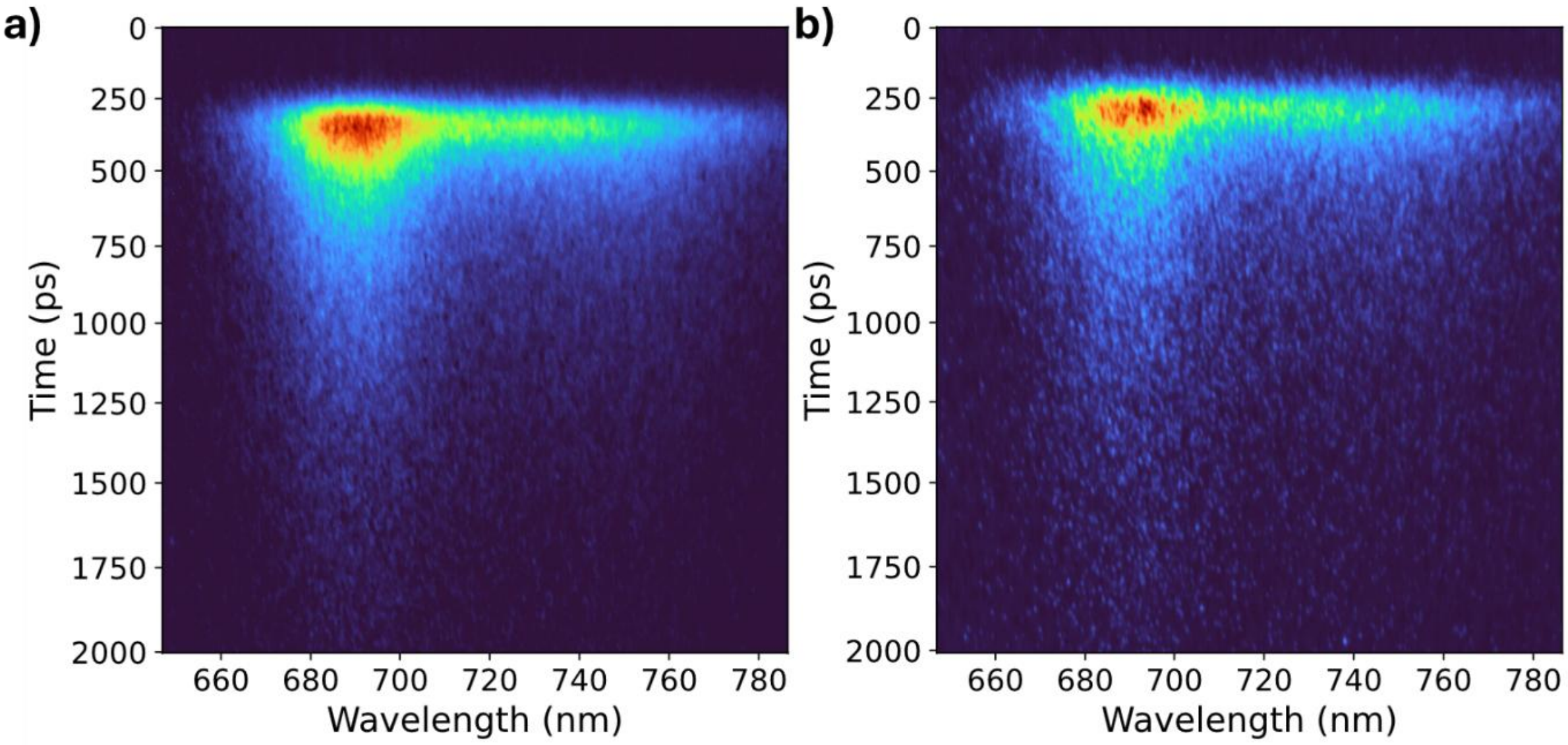

Figure S6: Representative ultrafast fluorescence spectra of wt *A. thaliana* under a) F_o and b) F_o' conditions.

Notes S2: Rates of NPQ for Open and Closed ^{PSII}RCs – PSII Lifetimes

A previous study[31] compared the rates of NPQ for open and closed ^{PSII}RCs using the average PSII lifetimes obtained from ultrafast measurements. Using the average lifetimes presented in the results section we find the following values of k_{NPQ}^{Closed} and k_{NPQ}^{Open} for wt *A. thaliana*:

$$k_{NPQ}^{Closed} = \frac{1}{\langle \tau_{F'_m}^{PSII} \rangle} - \frac{1}{\langle \tau_{F_m}^{PSII} \rangle} = (1.2 \pm 0.1)\, ns^{-1}$$

$$k_{NPQ}^{Open} = \frac{1}{\langle \tau_{F'_o}^{PSII} \rangle} - \frac{1}{\langle \tau_{F_o}^{PSII} \rangle} = (0.7 \pm 0.1)\, ns^{-1}$$

Using these values yield the following ratio for the rates of NPQ for open and closed ^{PSII}RCs:

$$k_{NPQ}^{Open} / k_{NPQ}^{Closed} = (0.6 \pm 0.1)$$

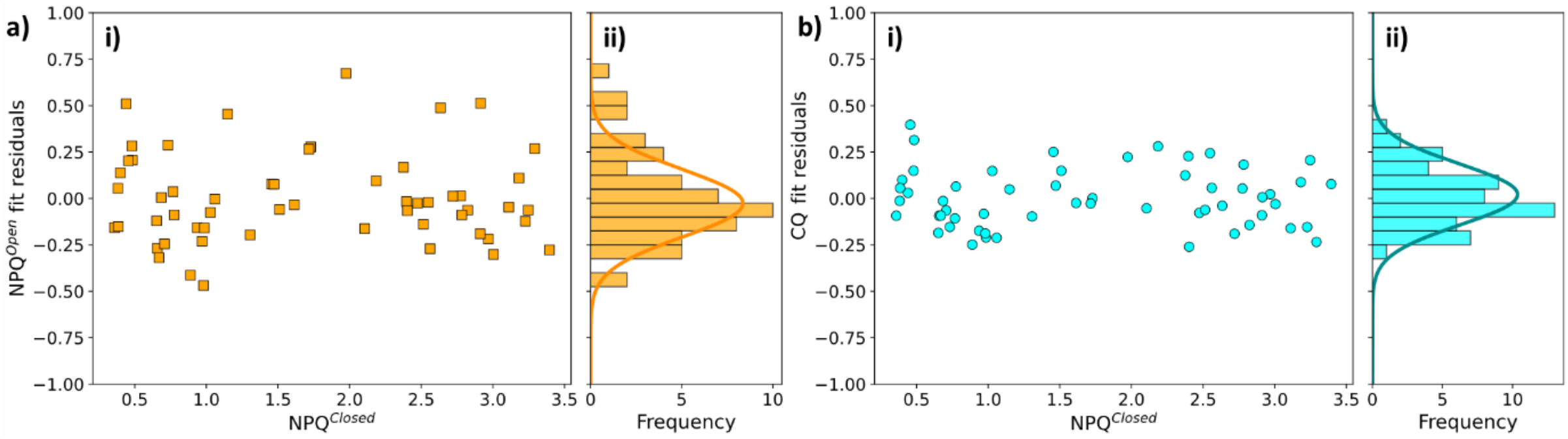

Figure S7: Residuals for the a).(i) NPQ^{Open} vs NPQ^{Closed} correlation as well as (ii) the fitted distribution of these residuals. The residuals for the b).(i) CQ vs NPQ^{Closed} correlation and (ii) the fitted distribution of these residuals.

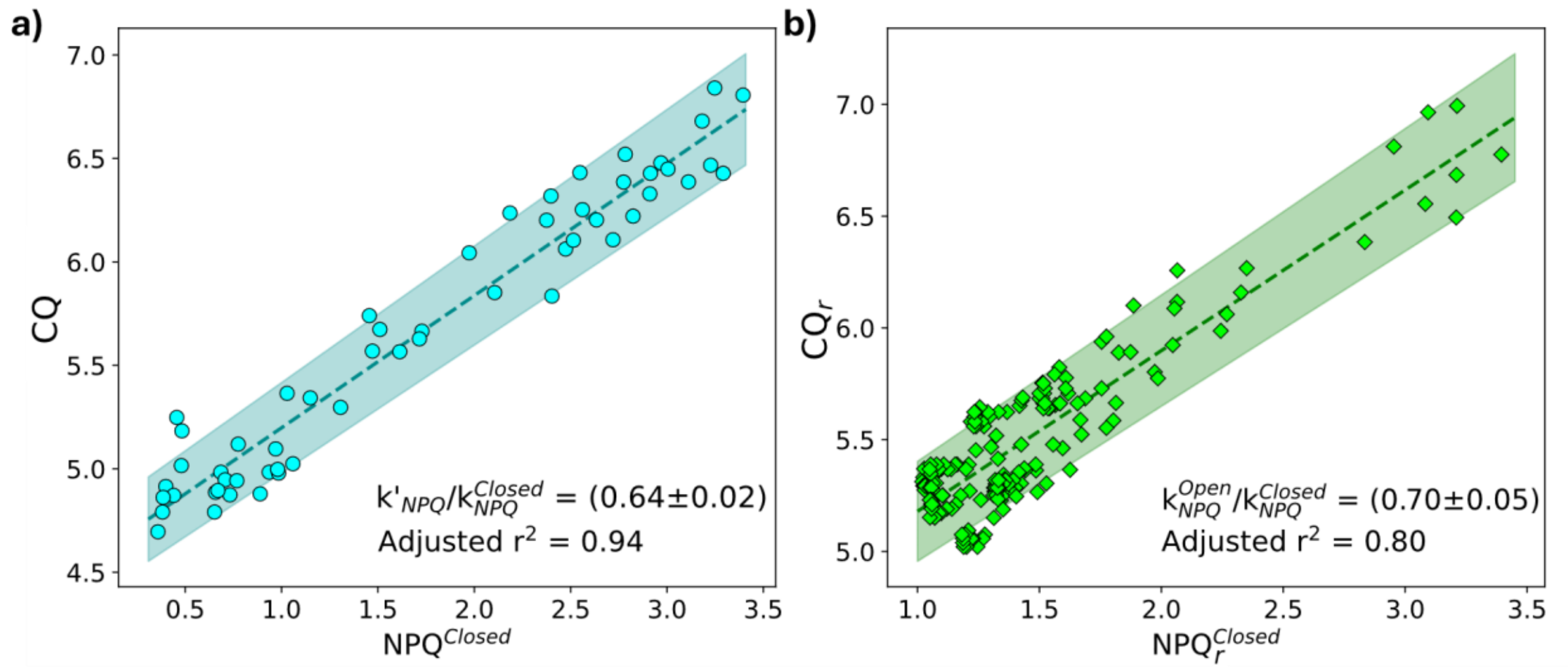

Figure S8: Fitted correlation plot of CQ vs NPQ^{Closed}, with an insert showing the average values of k_p^*/k_p (where $CQ = (F_m - F')/F' = PQ^* + mNPQ^{Closed}$, is the sum of the photochemical and non-photochemical quenching, $NPQ^{Closed} = k_{NPQ}^{Closed}/(k_f + k_d)$ is the value of NPQ when the Q_A pool is fully reduced (fully closed ^{PSII}RCs), k_p^* is the apparent rate of photochemical quenching when the Q_A pool is partially reduced (^{PSII}RCs are partially open), k_p is the rate of photochemical quenching when the Q_A pool is fully oxidised (all ^{PSII}RCs are open) and $PQ^* = k_p^*/(k_f + k_d)$ is the photochemical quenching when the Q_A pool is partially reduced (^{PSII}RCs are partially open)), for *stn7 A. thaliana* (calculated with yields corrected

for the contribution of PSI).

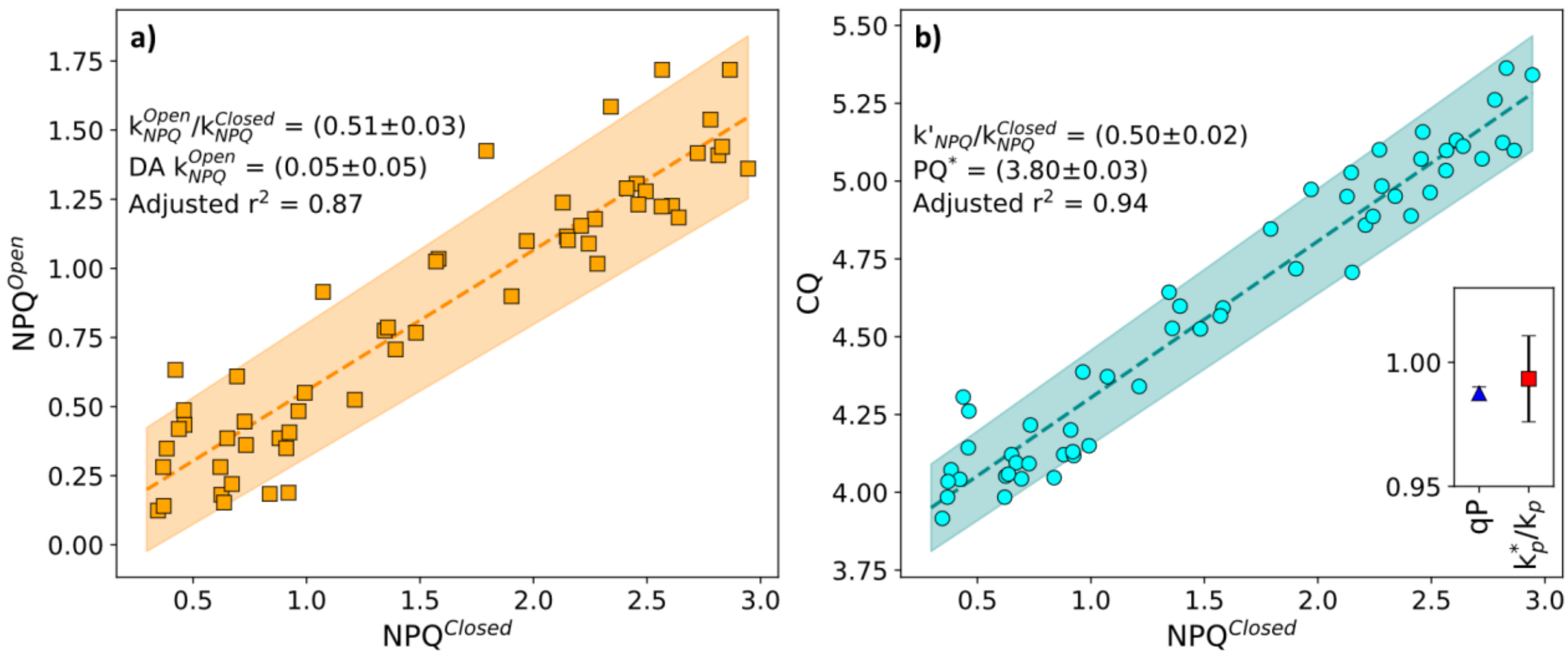

Figure S9: Fitted correlation plots of a) NPQ^{Open} vs NPQ^{Closed} (including the results of the ultrafast fluorescence measurements, magenta diamond) and b) CQ vs NPQ^{Closed}, with an insert comparing the average values of qP and k_p^*/k_p, for wt *A. thaliana*, without correcting for the fluorescence contribution of PSI. All of the values were calculated at the end of each 10 minute illumination step in the light response curve. All plants were dark adapted overnight prior to measurement and illuminated with red actinic light.

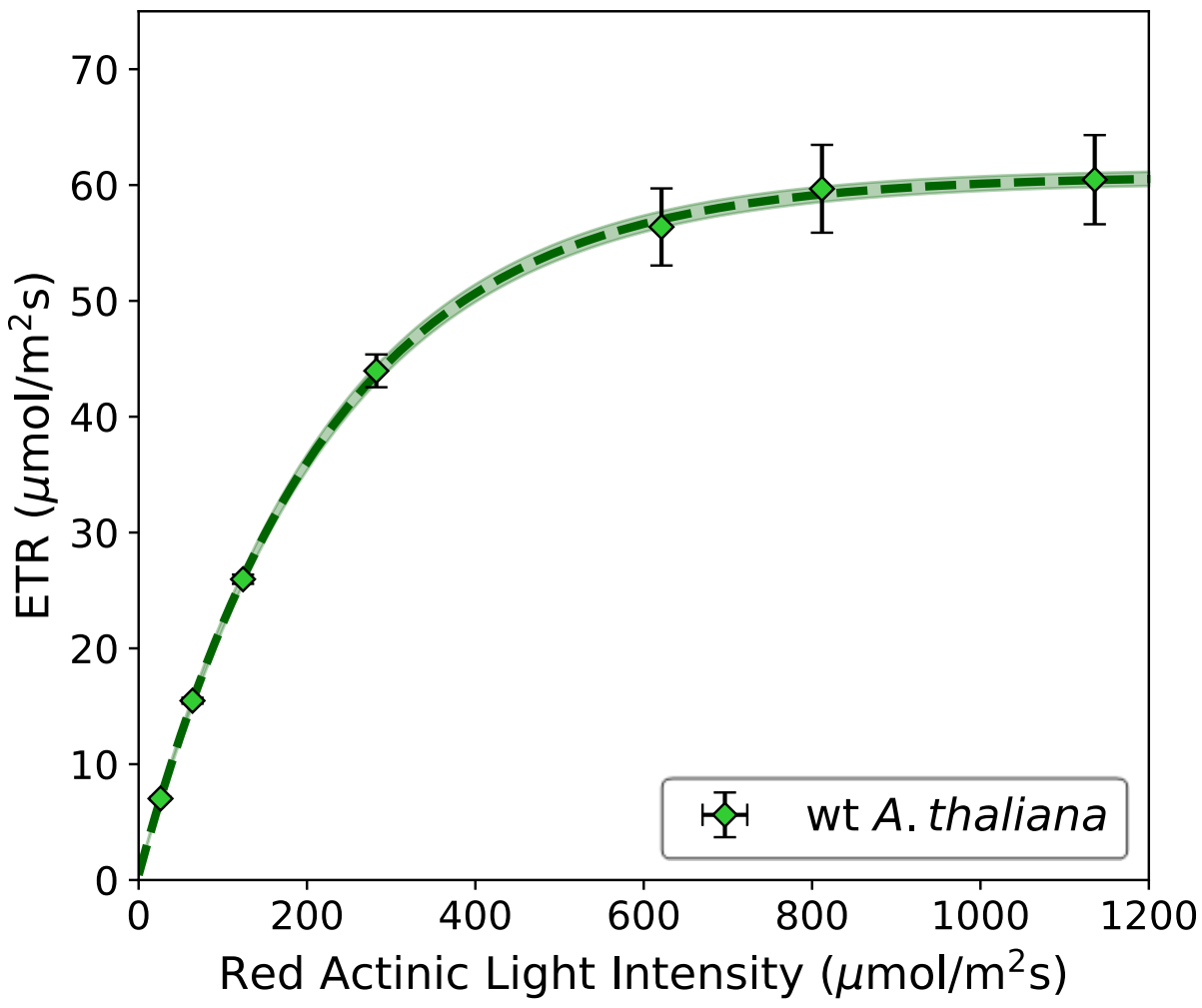

Figure S10: Average electron transport rate (ETR) vs red actinic light intensity for wt *A. thaliana*, obtained from the light response curve measurements performed on individual leaves. All plants were dark adapted overnight prior to measurement. The ETR is calculated using $ETR = \frac{PFDa}{2}((F'_m - F')/F'_m)$,[35,39] where $PFDa$ is the absorbed light (assumed to be 84% for the mini-PAM-II used here) and it is assumed that half of the absorbed energy is partitioned into PSII.

Notes S3: Equations and Numerical Examples

S3.1 – Fluorescence yields, lifetimes and calculating NPQ

The fluorescence yields or lifetimes measured in steady-state and ultrafast measurements can be expressed in terms of the rate constants associated with photochemical quenching, fluorescence, quenching due to both non-inducible and inducible thermal quenching of excitations in both the antenna and PSIIRCs.[37] These expressions are summarised in table S1:

***Table S1*:** Summary of the equations relating the fluorescence yields and lifetimes to the rate constants of the underlying physical and biological processes.

**Fluorescence Yield Equation**	**Fluorescence Lifetime Equation**
$F_m = \frac{k_f}{(k_f + k_d)} \quad (1a)$	$\tau_{F_m} = \frac{1}{(k_f + k_d)} \quad (1b)$
$F'_m = \frac{k_f}{(k_f + k_d + k_{NPQ})} \quad (2a)$	$\tau_{F'_m} = \frac{1}{(k_f + k_d + k_{NPQ})} \quad (2b)$
$F_o = \frac{k_f}{(k_f + k_d + k_p)} \quad (3a)$	$\tau_{F_o} = \frac{1}{(k_f + k_d + k_p)} \quad (3b)$
$F'_o = \frac{k_f}{(k_f + k_d + k_p + k_{NPQ})} \quad (4a)$	$\tau_{F'_o} = \frac{1}{(k_f + k_d + k_p + k_{NPQ})} \quad (4b)$
$F' = \frac{k_f}{(k_f + k_d + k_p^* + k_{NPQ})} \quad (5a)$	$\tau_{F'} = \frac{1}{(k_f + k_d + k_p^* + k_{NPQ})} \quad (5b)$

where k_f is the rate of fluorescence, k_d is the sum of the rates of interconversion and intersystem crossing, k_p is the rate of photochemical quenching when the Q_A pool is fully oxidised (all reaction centres are open), k_p^* is the apparent rate of photochemical quenching when the Q_A pool is partially reduced (PSIIRCs are partially open) and k_{NPQ} is the rate of non-photochemical quenching.

The Stern-Volmer equation can be used to calculate NPQ^{Closed} (NPQ in the presence of fully closed PSIIRCs):

$$NPQ^{Closed} = \frac{F_m - F'_m}{F'_m} = \frac{\tau_{F_m} - \tau_{F'_m}}{\tau_{F'_m}} = \frac{k_{NPQ}^{Closed}}{(k_f + k_d)} \quad (6)$$

S3.2 – Derivation of the F'_o version of the Stern-Volmer equation (NPQ^{Open}):

The F_o and F'_o fluorescence yield parameters are noted to represent leaves with fully open PSIIRCs. The only difference between these parameters is that the latter includes the NPQ induced following a period of illumination. This means that it is possible to obtain an expression for NPQ in the presence of fully open PSIIRCs (NPQ^{Open}) by considering the differences between these two parameters.

$$F_o - F_o' = \frac{k_f k_{NPQ}^{Open}}{(k_f + k_d + k_p)(k_f + k_d + k_p + k_{NPQ}^{Open})} = \frac{k_f}{(k_f + k_d + k_p + k_{NPQ}^{Open})}\left\{\frac{k_{NPQ}^{Open}}{(k_f + k_d + k_p)}\right\} \quad (8)$$

Since $F_o' = k_f/(k_f + k_d + k_p + k_{NPQ}^{Open})$, equation (8) simplifies to:

$$F_o - F_o' = F_o'\left\{\frac{k_{NPQ}^{Open}}{(k_f + k_d + k_p)}\right\} \Rightarrow \frac{(F_o - F_o')}{F_o'} = \left\{\frac{k_{NPQ}^{Open}}{(k_f + k_d + k_p)}\right\} \quad (9)$$

Since $NPQ^{Open} = k_{NPQ}^{Open}/(k_f + k_d)$ equation (9) can be rewritten as:

$$\frac{(F_o - F_o')}{F_o'} = NPQ^{Open}\left\{\frac{(k_f + k_d)}{(k_f + k_d + k_p)}\right\} \quad (10)$$

Finally, since $F_m = k_f/(k_f + k_d)$ and $F_o = k_f/(k_f + k_d + k_p)$, the equation (10) simplifies to:

$$\frac{(F_o - F_o')}{F_o'} = NPQ^{Open}\left\{\frac{F_o}{F_m}\right\} \quad (11)$$

Rearranging equation (11) yields the following equation for NPQ^{Open}:

$$NPQ^{Open} = \left(\frac{F_o - F_o'}{F_o'}\right)\frac{F_m}{F_o} = \left(\frac{\tau_{F_o} - \tau_{F_o'}}{\tau_{F_o'}}\right)\frac{\tau_{F_m}}{\tau_{F_o}} = \frac{k_{NPQ}^{Open}}{(k_f + k_d)} \quad (12)$$

S3.3 – Derivation of the F' version of the Stern-Volmer equation (NPQ'):

The F' fluorescence yield parameter is noted to represent leaves with NPQ in the presence of partially open ^{PSII}RCs. Therefore it is possible to use this parameter to obtain an expression for NPQ (NPQ', which yields the associated quenching rate k_{NPQ}') in the presence of partially open ^{PSII}RCs.

$$F' = \frac{k_f}{(k_f + k_d + k_p^* + k_{NPQ}')} \Rightarrow \frac{1}{F'} = \frac{(k_f + k_d + k_p^* + k_{NPQ}')}{k_f} \quad (13)$$

Using $F_m = k_f/(k_f + k_d)$, equation (13) can be rewritten as:

$$\frac{1}{F'} = \frac{k_p^*}{k_f} + \frac{k_{NPQ}'}{k_f} + \frac{(k_f + k_d)}{k_f} = \frac{k_p^*}{k_f} + \frac{k_{NPQ}'}{k_f} + \frac{1}{F_m} \quad (14)$$

Equation (14) can be rearranged to:

$$\frac{1}{F'} - \frac{1}{F_m} = \frac{k_p^*}{k_f} + \frac{k_{NPQ}'}{k_f} \quad (15)$$

Multiplying through by F_m equation (15) simplifies to:

$$\frac{F_m}{F'} - 1 = \frac{F_m - F'}{F'} = \frac{k_p^*}{(k_f + k_d)} + \frac{k'_{NPQ}}{(k_f + k_d)} \quad (16)$$

Since $NPQ' = k'_{NPQ}/(k_f + k_d)$ and defining two similar quenching parameters for the photochemical related quenching as $PQ^* = k_p^*/(k_f + k_d)$ and the combined quenching as $CQ = (k_p^* + k'_{NPQ})/(k_f + k_d)$ allows equation (16) to be rewritten as:

$$\frac{F_m - F'}{F'} = CQ = PQ^* + NPQ' \quad (17)$$

Assuming $NPQ' = mNPQ^{Closed}$, where m is a dimensionless non-negative real number ($\mathbb{R}_{>0}$), equation (17) can be simplified as:

$$CQ = PQ^* + mNPQ^{Closed} \quad (18)$$

S3.4 – Development of the Numerical Simulations of the NPQ Correlations

To understand the NPQ^{Open} vs NPQ^{Closed} and CQ vs NPQ^{Closed} correlations a series of numerical simulations were preformed using equations (1a)-(5a). In these simple numerical simulations the value of F_m was normalised to 1, the value of k_p was set to 2.9 ns^{-1} and the value of $(k_f + k_d)$ was set to 0.6 ns^{-1}. These values of k_p and $(k_f + k_d)$ were calculated using the average PSII lifetimes measured in the F_m and F_o states. Using these values the numerical simulation yielded a value of F_o = 0.171 and F_v/F_m = 0.829. These values are fully consistent with PAM values obtained for unstressed, fully dark adapted leaves after correcting for the contribution of PSI.

Using a range of k_{NPQ} values, the corresponding values of F'_m, F'_o and F' were calculated using equations (2a), (4a) and (5a). Random noise was added to each of these parameters to simulate biological variance and instrument noise, these random noise values were chosen from a Gaussian distribution with a FWHM of 5%. The values of NPQ^{Closed}, NPQ^{Open} and CQ were then calculated (equations outlined in S3.1-3.3) for each supplied value of k_{NPQ}. To simulate the measurements of individual leaves, one of the generated NPQ^{Closed}, NPQ^{Open} and CQ values is chosen and added to the simulated data. This process is then repeated, using new randomised noise, until 70 simulated leaves have been sampled. The resulting NPQ^{Open} vs NPQ^{Closed} and CQ vs NPQ^{Closed} correlations are shown in figure S11.

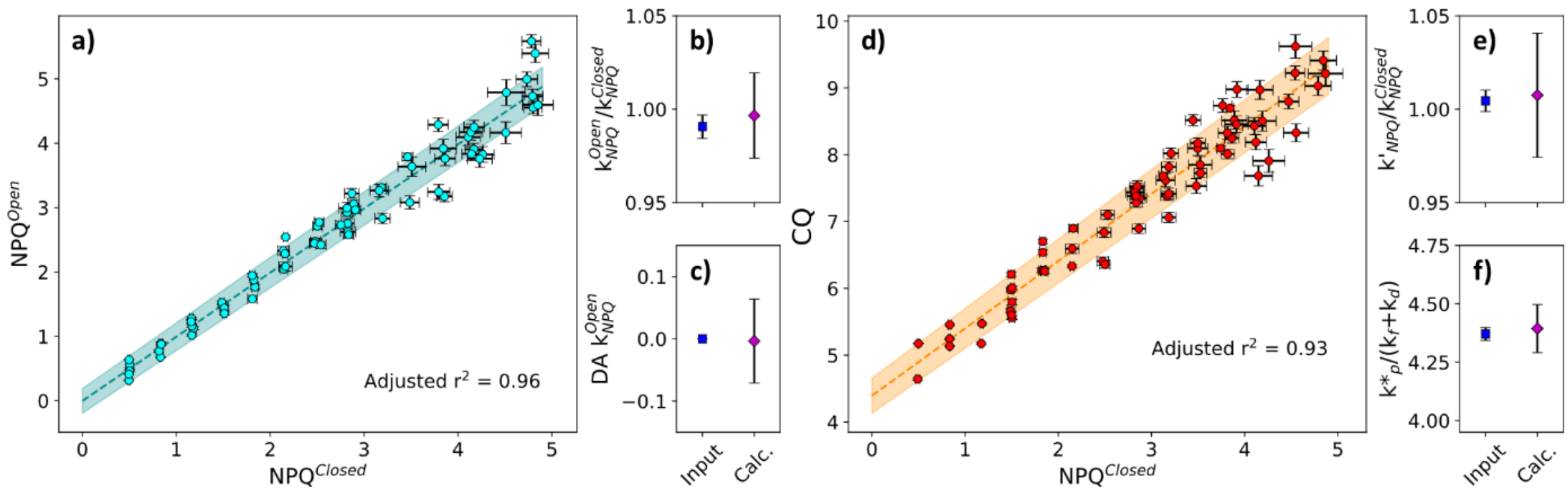

Figure S11: Results of the numerical simulation for the a) NPQ^{Open} vs NPQ^{Closed} correlation comparing the calculated and input b) gradients and c) intercepts; The d) CQ vs NPQ^{Closed} correlation comparing the calculated and input e) gradients and f) intercepts. Assumptions: No ^{PSII}RC induced NPQ rate switching (*e.g.* in these simulations $k_{NPQ}^{Closed} = k_{NPQ}^{Open}$) and no state transitions or chloroplast movement occurs.

These simulations reveal that both NPQ^{Open} and CQ show a strong linear correlation with NPQ^{Closed}, exhibiting adjusted r² values above 0.9 (Figs. S11.(a) & (d)). Additionally, the gradient of these correlation is found to be the ratio between the value of NPQ calculated in the presence of different amounts of photochemical quenching. The close agreement between the theoretical input and calculated parameters (Figs. S11.(b),(c),(e) &(f)) show that these numerical simulations can be used to explore the fluorescence yield correlations and so these simulations will allow the effect(s) of different physical/biological processes on the correlations to be explored and compared to the experimental correlations. These comparisons will allow the source of the observed NPQ discrepancy to be identified. The simulations for the effects of state transitions, NPQ heterogeneity, qI-like processes and RC-induced NPQ rate switching are outlined in S3.5-3.8.

S3.5 –Effects of illumination driven changes in fluorescence yield

Several illumination induced biological processes are known to lead to small changes in the values of the chlorophyll fluorescence yield obtained during steady-state measurements. Since all of the methodologies outlined here to calculate the NPQ compare fluorescence yield values obtained before illumination (F_m and F_o) with those obtained during or directly after illumination (F_m', F_o' and F') it is important to understand how such changes in the leaves alter the NPQ^{Open} vs NPQ^{Closed} and CQ vs NPQ^{Closed} correlations. In *A. thaliana* dark adapted plants there are two biological mechanisms that are known to change the size of several fluorescence yield parameters (F_m', F_o' and F'), state transitions[42–44] and chloroplast movement.[45–47] While the use of red actinic light in the fluorescence yield measurements eliminates chloroplast movement, upon illumination of fully dark adapted plants, state transitions lead to the movement of a portion of

antenna complexes from PSII to PSI (referred to as a state 1 to state 2 transition) and *vice versa* (referred to as a state 2 to state 1 transition). Dark-adaptation is known to induce state 1 in vascular plants, and upon illumination these plants undergo transition from state 1 to state 2.[42–44] Since this transition leads to a portion of antenna complexes moving from PSII to PSI this means that illumination of dark-adapted plants leads to small decreases in the fluorescence yield parameters obtained during or directly after illumination (F'_m, F'_o and F').[42–44] To reflect this the model underpinning the numerical simulation was updated to model a decrease in the fluorescence yield parameters obtained during or directly after illumination to account for these optical changes in the leaf. A summary of the numerical simulations run to explore the effect of state transitions is shown in figure S12.

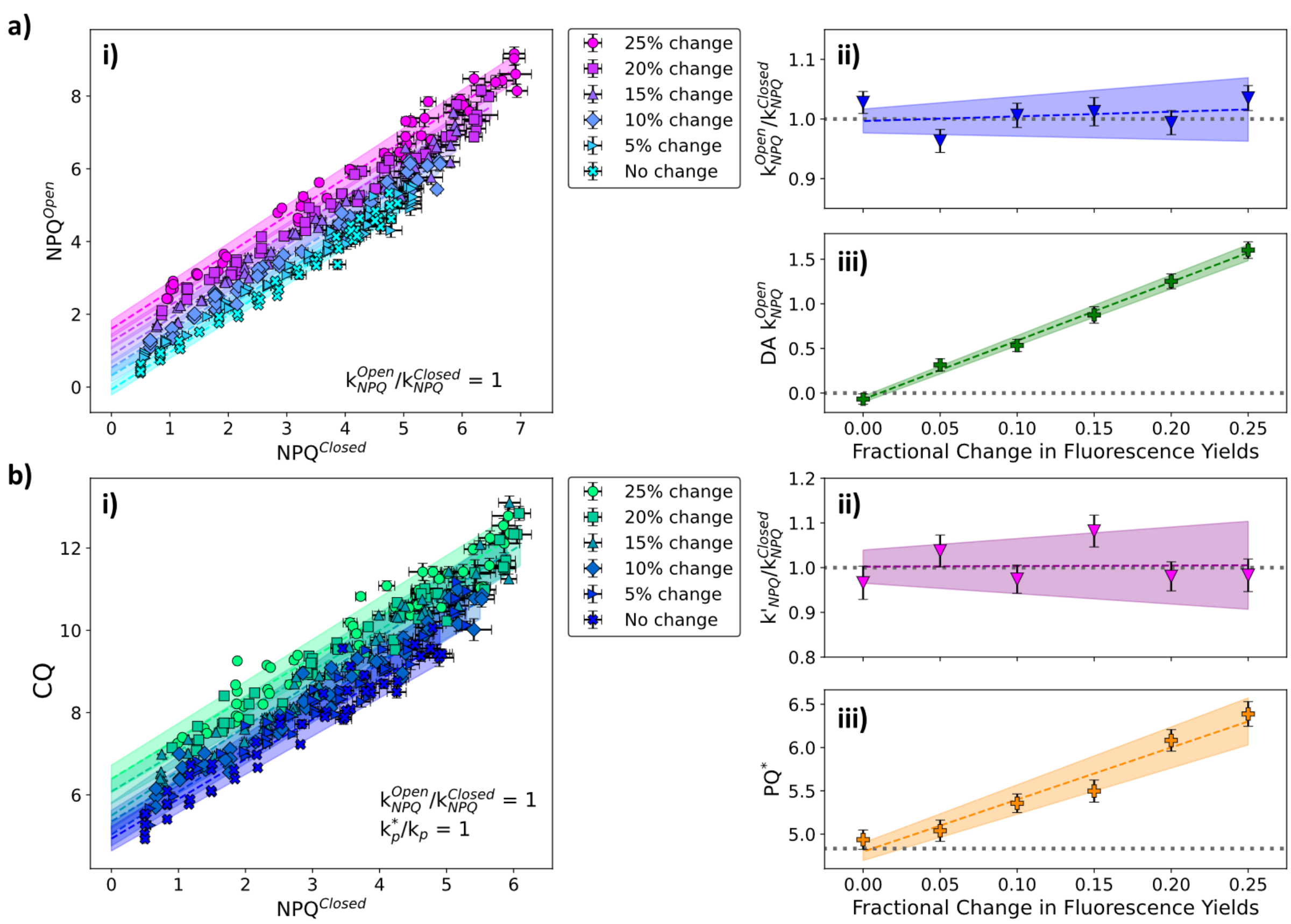

Figure S12: a) Results of the numerical simulation for the i) NPQ^{Open} vs NPQ^{Closed} correlation as well as the calculated ii) gradients and iii) intercepts; b) Results of the numerical simulation for the i) CQ vs NPQ^{Closed} correlation as well as the calculated ii) gradients and iii) intercepts. Assumptions: No ^{PSII}RC induced NPQ rate switching (e.g. in these simulations $k^{Closed}_{NPQ} = k^{Open}_{NPQ}$) and the percent change is caused by either state transitions or chloroplast movements.

These numerical simulations reveal that these illumination induced changes in the fluorescence yield increase the intercepts of the correlations leading to these moving away from the expected

value (dotted grey lines Fig. S12.(a).(iii)& S12.(b).(iii)). However the gradient of these correlations is unaffected by these changes in fluorescence yield (dotted grey lines Fig. S12.(a).(ii)& S12.(b).(ii)).

Since *stn7 A. thaliana* lacks state transitions it corresponds to the 'No change' scenario in Fig.S12.(b). The CQ versus NPQ^{Closed} correlation for this mutant is shown in figure S6. Correcting for the fact that not all the PSIIRCs were fully reopened (outlined in S3.8) this correlation reveals that in the absence of state transitions $\mathrm{k}_{\mathrm{NPQ}}^{\mathrm{Open}}/\mathrm{k}_{\mathrm{NPQ}}^{\mathrm{Closed}} = (0.72 \pm 0.09)$ (Fig.S13). This is noted to be the same (within error) as the wt *A. thaliana* value (obtained in the presence of state transitions), showing that state transitions do not contribute to the observed discrepancy, in complete agreement with our numerical simulations.

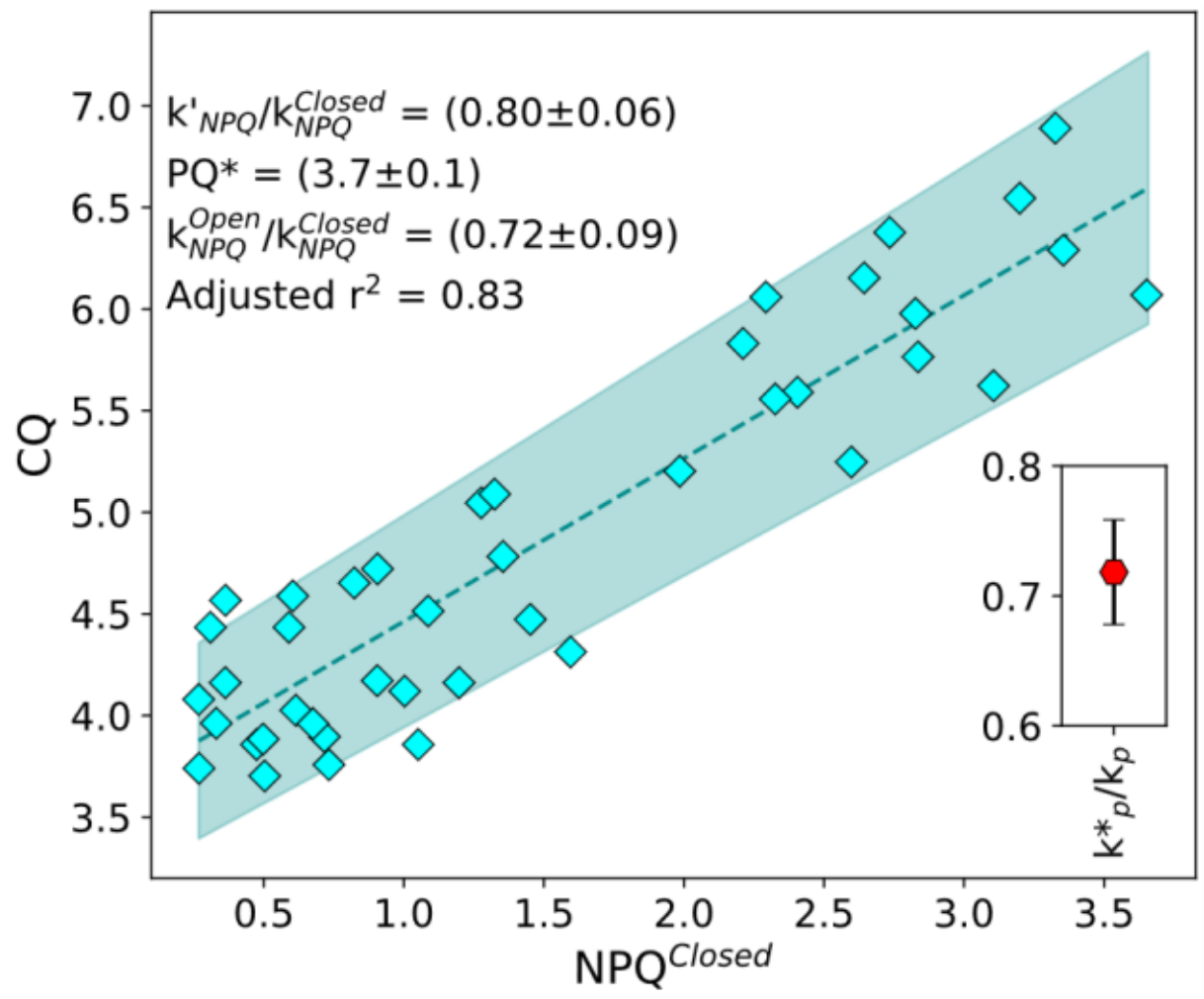

Figure S13: Fitted correlation plot of CQ vs NPQ^{Closed}, with an insert showing the average values of k_p^*/k_p (where $CQ = (F_m - F')/F' = PQ^* + mNPQ^{Closed}$, is the sum of the photochemical and non-photochemical quenching, $NPQ^{Closed} = k_{NPQ}^{Closed}/(k_f + k_d)$ is the value of NPQ when the Q_A pool is fully reduced (fully closed PSIIRCs), k_p^* is the apparent rate of photochemical quenching when the Q_A pool is partially reduced (PSIIRCs are partially open), k_p is the rate of photochemical quenching when the Q_A pool is fully oxidised (all PSIIRCs are open) and $PQ^* = k_p^*/(k_f + k_d)$ is the photochemical quenching when the Q_A pool is partially reduced (PSIIRCs are partially open)), for *stn7 A. thaliana* (calculated with yields corrected for the contribution of PSI).

S3.6 – Effect of NPQ Compartment Heterogeneity

NPQ heterogeneity is envisioned to arise from the presence of PSII super-complexes exhibiting NPQ with different quenching rates. For clarity here we will consider an ensemble consisting of two such photosynthetic compartments. These compartments are of equal size and only exhibit differing NPQ quenching rates. From equations (1a) and (3a) it can be concluded that the values

for F_m and F_o are unaffected by this type of heterogeneity between the compartments, as these fluorescence yields are measured in the absence of NPQ. Here, equations (2a), (4a) and (5a) indicate that this heterogeneity will lead to different values of F_m', F_o' and F' for each of the compartments. In PAM measurements the measuring light illuminates a relatively large area on the leaf and so these measurements will yield weighted average values of F_m', F_o' and F'. Altering the numerical simulations to calculate the weighted averages of F_m', F_o' and F' allows the effect of NPQ heterogeneity on the NPQ^{Open} versus NPQ^{Closed} and CQ versus NPQ^{Closed} correlations to be explored. The effect of NPQ heterogeneity on the NPQ^{Open} versus NPQ^{Closed} correlation is summarised in figure S14.

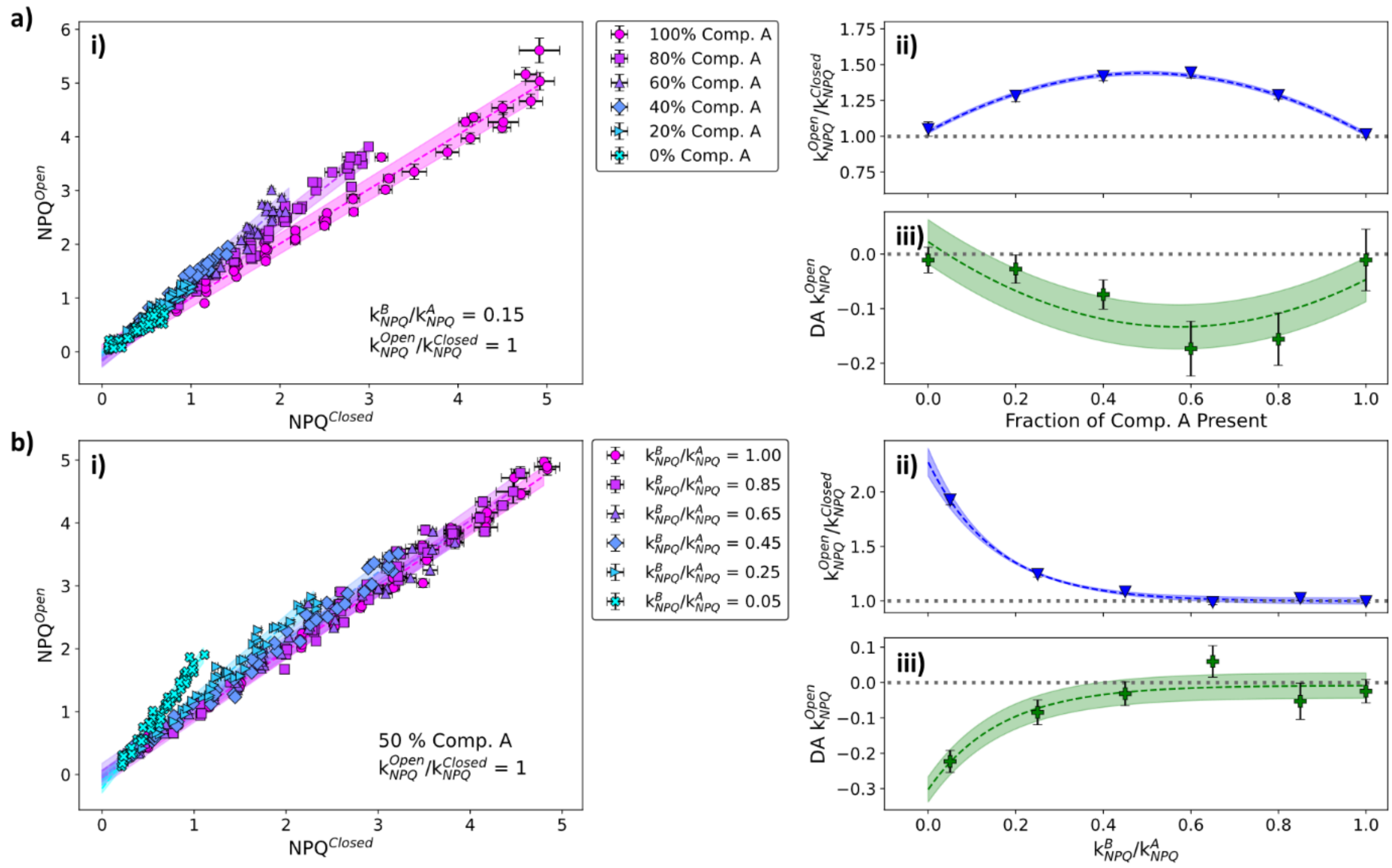

Figure S14: a) Results of the numerical simulation of the effect of different compartment sizes in the approximate model of NPQ heterogeneity for the i) NPQ^{Open} vs NPQ^{Closed} correlation as well as the calculated ii) gradients and iii) intercepts; b) Results of the numerical simulation for of the effect of different compartment quenching rates in the approximate model of NPQ heterogeneity the i) NPQ^{Open} vs NPQ^{Closed} correlation as well as the calculated ii) gradients and iii) intercepts. Assumptions: No ^{PSII}RC induced NPQ rate switching (e.g. in these simulations $k_{NPQ}^{Closed} = k_{NPQ}^{Open}$) and no state transitions or chloroplast movements occur.

Here the NPQ heterogeneity is two dimensional, with both the proportion of each compartment as well as the ratio between their NPQ quenching rates leading to changes in the NPQ^{Open} vs NPQ^{Closed} correlation. If the compartment size is varied at a constant ratio between the

compartment quenching rates (k_{NPQ}^{B}/k_{NPQ}^{A}; chosen to be 0.15 in these numerical simulations) both the gradient and intercept move away from the expected value (dotted grey lines, Fig. S14.(a).(ii)&(iii)). The discrepancy has a maximum when each compartment represents 50% of the system and it is noted that this heterogeneity leads to a larger than expected value for the gradient and a lower than expected value for the intercept. Alternatively if the ratio between the compartment quenching rates (k_{NPQ}^{B}/k_{NPQ}^{A}) changes for a fixed compartment size (Comp. A; chosen to be 50% in these numerical simulations) both the gradient and intercept move away from the expected value (dotted grey lines, Fig. S14.(b).(ii)&(iii)). Here the observed discrepancies increase as the ratio between the quenching rates increases (*e.g.* as $k_{NPQ}^{B}/k_{NPQ}^{A} \rightarrow 0$). Again the gradient is overestimated and the intercept is underestimated. The effect of NPQ heterogeneity on the CQ versus NPQ^{Closed} correlation is summarised in figure S15.

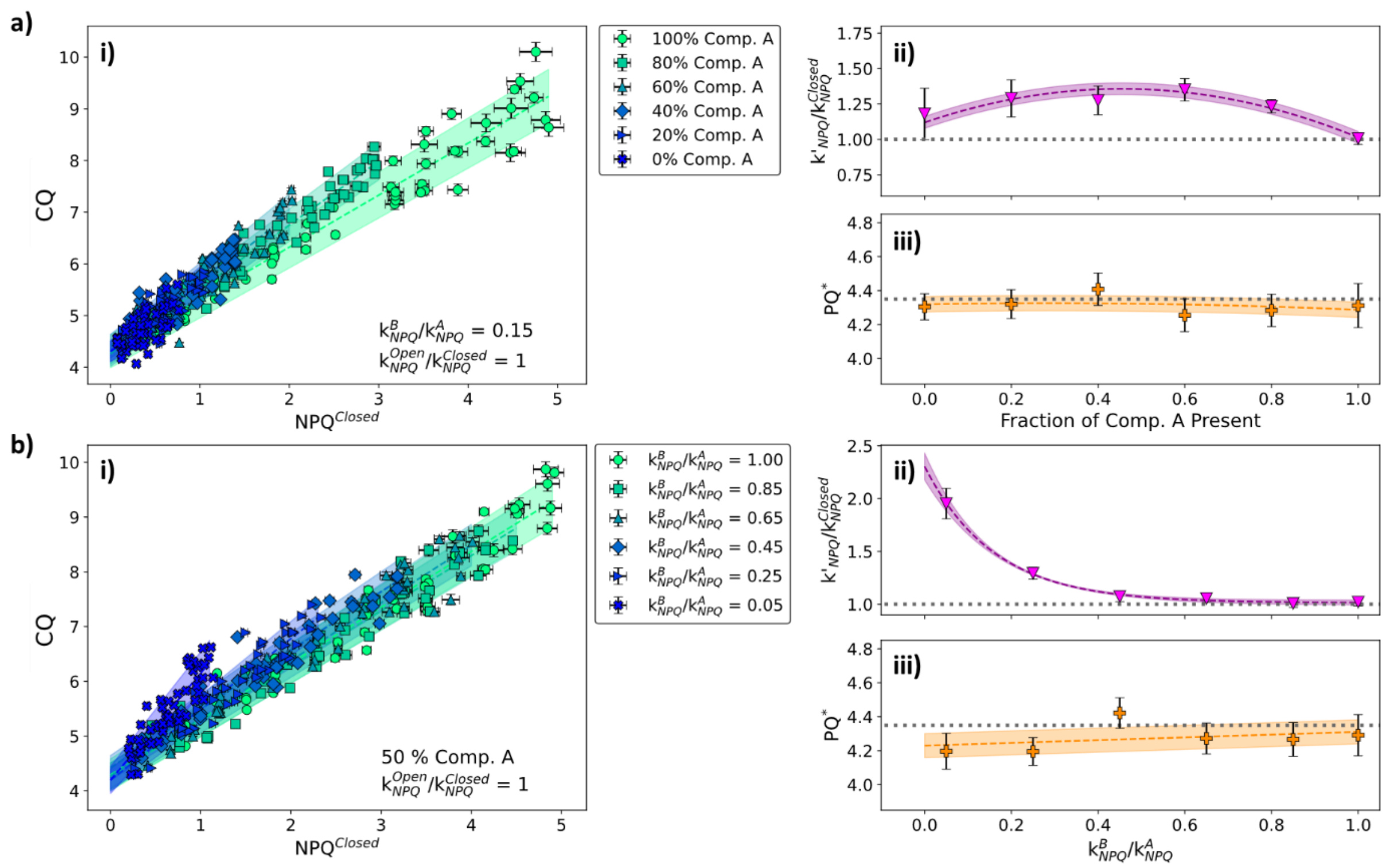

Figure S15: a) Results of the numerical simulation of the effect of different compartment sizes in the approximate model of NPQ heterogeneity for the i) CQ vs NPQ^{Closed} correlation as well as the calculated ii) gradients and iii) intercepts; b) Results of the numerical simulation of the effect of different compartment quenching rates in the approximate model of NPQ heterogeneity the i) CQ vs NPQ^{Closed} correlation as well as the calculated ii) gradients and iii) intercepts. Assumptions: No ^{PSII}RC induced NPQ rate switching (e.g. in these simulations $k_{NPQ}^{Closed} = k_{NPQ}^{Open}$) and no state transitions or chloroplast movements occur.

Similar to the other correlations, both compartment size and the ratio between the quenching rates lead to discrepancies between the observed and extracted gradients. Again these gradients

are overestimated. For the compartment size this effect is largest when both compartments contribute to the system equally (*e.g.* 50 % Comp. A) and as the ratio between the quenching rates increases (*e.g.* as $k^B_{NPQ}/k^A_{NPQ} \to 0$). However, for the CQ vs NPQ^{Closed} correlation, NPQ heterogeneity does not affect the intercept.

S3.7 – Effects of qI-like processes on the CQ vs NPQ^{Closed} correlation

In addition to rapidly reversible NPQ (qE), illumination can also result in several slowly reversible pH-independent NPQ and photodamage (qI-like) processes being activated. These processes accumulate during illumination, meaning that they only contribute to the fluorescence yields obtained during or after illumination (F'_m, F'_o and F'). As such it is important to understand how these processes affect the fluorescence yield correlations. Since these processes are slowly reversible, they are approximately constant during the first several minutes (<10 minutes) of the post-illumination NPQ relaxation. For the pH-independent NPQ processes, as a first approximation these processes provide the same constant additional source of quenching to both F'_m and F' during the initial post-illumination recovery period and so change CQ and NPQ^{Closed} by the same amount. This means these processes do not affect the CQ vs NPQ^{Closed} correlation obtained during the post-illumination recovery. However, as a first order approximation the qI-like processes lead to an additional source of quenching (hereafter referred to as k_{qI}) and lead to the accumulation of damaged ^{PSII}RCs (hereafter this portion of damaged ^{PSII}RCs is referred to as α). To reflect this, the model underpinning the numerical simulation was updated to model the impact of the approximately constant qI in the fluorescence yield parameters obtained after illumination. A summary of the numerical simulations run to explore the effect of qI in the initial post-illumination period is shown in figure S16.

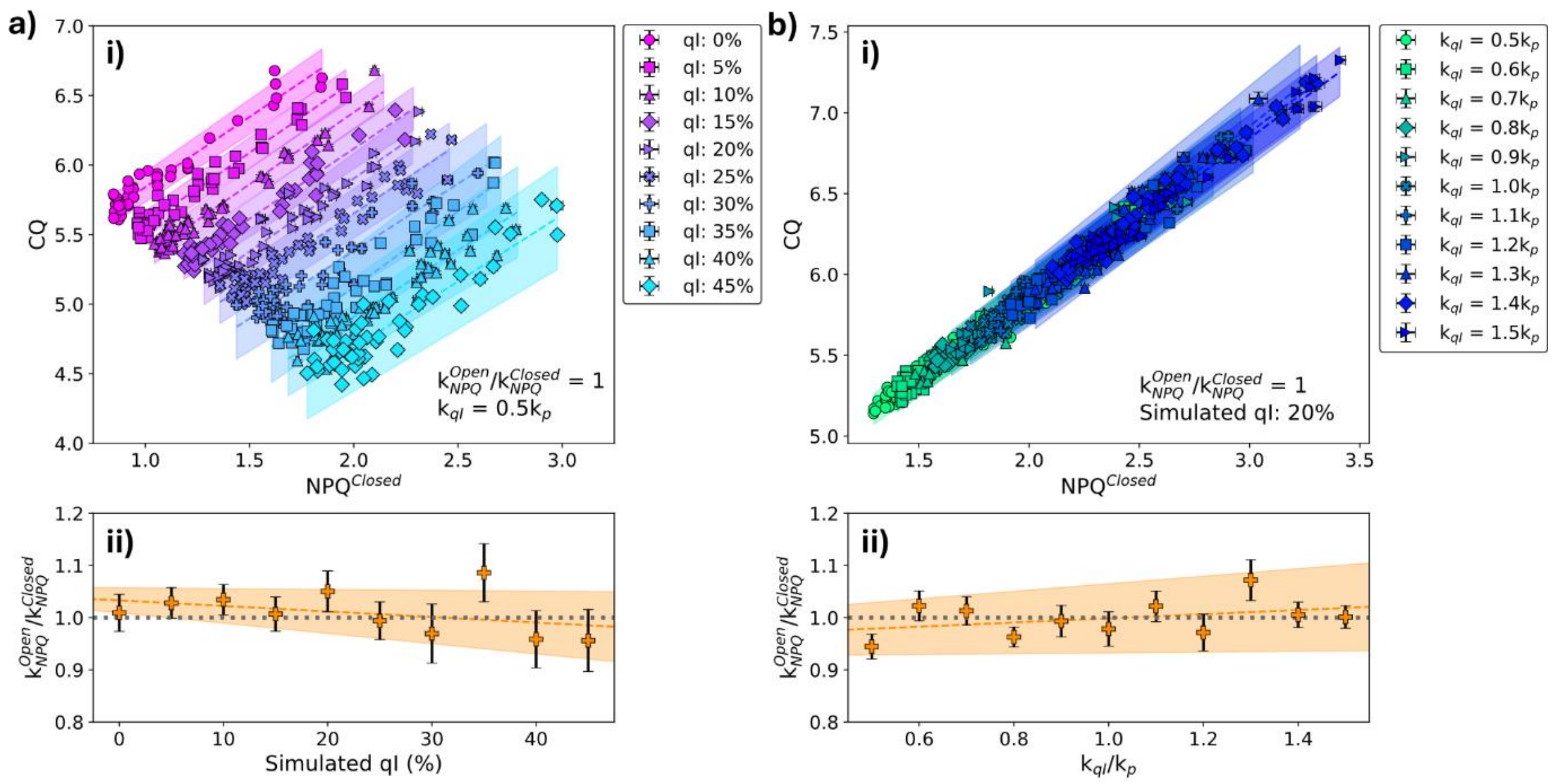

Figure S16: a) Results of the numerical simulation of the effect of different constant amounts of qI (α) on the i) CQ vs NPQ^{Closed} correlation as well as the calculated ii) $k_{NPQ}^{Open}/k_{NPQ}^{Closed}$; b) Results of the numerical simulation of the effect of different qI quenching rate (k_{qI}) on the i) CQ vs NPQ^{Closed} correlation as well as the calculated ii) $k_{NPQ}^{Open}/k_{NPQ}^{Closed}$. Assumptions: No ^{PSII}RC induced NPQ rate switching (e.g. in these simulations $k_{NPQ}^{Closed} = k_{NPQ}^{Open}$).

In the case of varying amounts of qI, the most significant effect on the CQ vs NPQ^{Closed} correlation is a change in the intercept (Fig.S16.(a).(i)), however the gradient is largely unaffected (Fig.S16.(a).(ii)). Furthermore, varying the value of k_{qI} was found to leave both the intercept and the gradient of the correlation largely unaffected (Fig.S16.(b)). This shows that a constant amount of accumulated qI does not affect the gradient of the CQ vs NPQ^{Closed} correlation and so does not affect the obtained $k_{NPQ}^{Open}/k_{NPQ}^{Closed}$ value. The results of these simulations are consistent with the experimentally observed CQ_r vs NPQ_r^{Closed} correlations during the post-illumination period (Fig.S8.(b)) observed for wt *A. thaliana*. These correlations yields a $k_{NPQ}^{Open}/k_{NPQ}^{Closed}$ value of (0.70 ± 0.05) (consistent within error with the values obtained from the NPQ^{Open} vs NPQ^{Closed} and CQ vs NPQ^{Closed} correlation obtained during the light response curve (Fig.4)). The small amount of spread in the experimental CQ_r vs NPQ_r^{Closed} correlations reveal that the small amount of qI accumulated during the wt *A. thaliana* light response curve measurements. Interestingly, the predicted spread in intercepts due to different overall amounts of qI can be seen in both the *npq4* and *npq1* CQ_r vs NPQ_r^{Closed} correlations (Fig.5). This is thought to be due to fact that these mutants, which exhibit lower overall amounts of NPQ, are more susceptible to photodamage.

S3.8 – Effect of ^{PSII}RC state dependent NPQ rate switching

The ^{PSII}RC state dependent NPQ rate switching model assumes that the rate of NPQ is directly affected by the state of the reaction centre.[31,32] To understand the effect of this on the fluorescence yield correlations the numerical simulations are altered to allow the rate on NPQ for fully open and fully closed ^{PSII}RCs to be different (*e.g.* $k_{NPQ}^{Open} \neq k_{NPQ}^{Closed}$). Additionally, it is assumed that the observed NPQ quenching rate seen for partially open ^{PSII}RCs (k'_{NPQ}) is the weighted average between k_{NPQ}^{Open} and k_{NPQ}^{Closed} (where the weighting is determined by the fraction of open ^{PSII}RCs). The results of these simulations are summarised in figure S17.

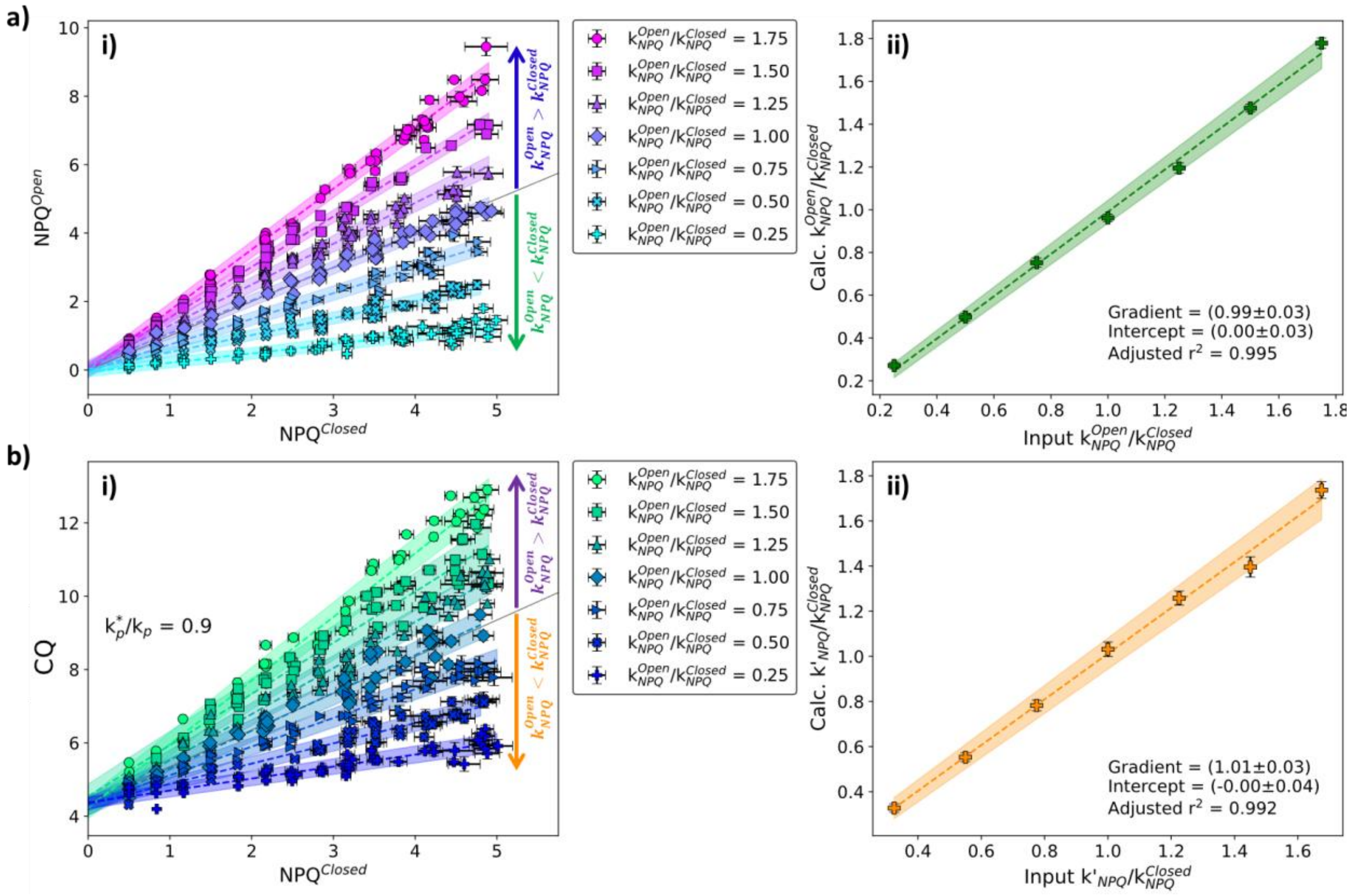

Figure S17: a) Results of the numerical simulation for the i) NPQ^{Open} vs NPQ^{Closed} correlation as well as a comparison between the calculated and input values of ii) $k_{NPQ}^{Open}/k_{NPQ}^{Closed}$; b) Results of the numerical simulation for the i) CQ vs NPQ^{Closed} correlation as well as a comparison between the calculated and input values of ii) $k'_{NPQ}/k_{NPQ}^{Closed}$; in these simulations $k_p^*/k_p = 0.9$. Assumptions: No state transitions or chloroplast movements occur and the induction of NPQ is homogenous.

Here, the simulations reveal that for both correlations the ^{PSII}RC induced switching of the NPQ quenching rate has no significant effect on the intercepts. However there is a clear effect on the gradients, with the gradient of both correlation decreasing from unity as $k_{NPQ}^{Open} < k_{NPQ}^{Closed}$ and increasing from unity as $k_{NPQ}^{Open} > k_{NPQ}^{Closed}$. Importantly, for both correlations the gradient is identical to the ratio between k_{NPQ}^{Open} and k_{NPQ}^{Closed} (or k'_{NPQ} and k_{NPQ}^{Closed} for the CQ vs NPQ^{Closed} correlation, if F' is obtained when the ^{PSII}RCs are only partially open).

S3.9 – Effects of partially closed PSII ^{PSII}RCs on the CQ vs NPQ^{Closed} correlation

Since F' is normally obtained when the ^{PSII}RCs are partially open it is important to understand how this effects the CQ vs NPQ^{Closed} correlation. To understand if it is still possible to extract the ratio between k_{NPQ}^{Open} and k_{NPQ}^{Closed}, a range of numerical simulations were performed for different values of k_p^*/k_p ($\approx qP$). The results of these simulations are summarised in figure S18.

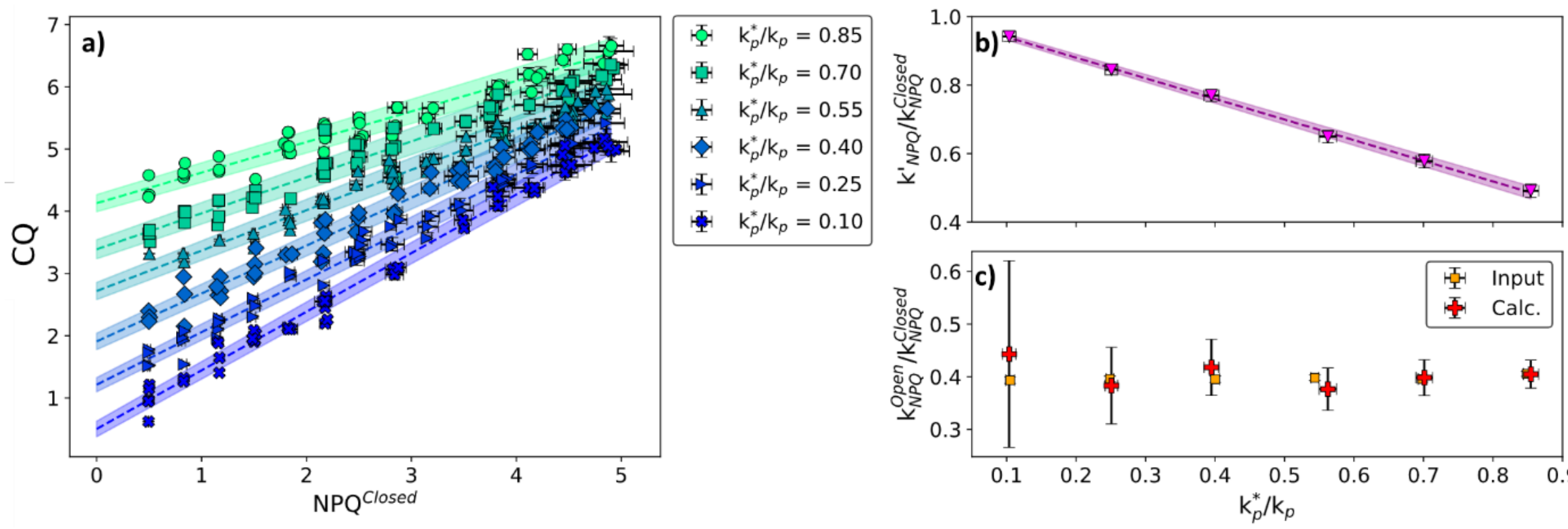

Figure S18: Results of the numerical simulation for the a) CQ vs NPQ^{Closed} correlation for a series of different values of k^*_p/k_p; the calculated values b) of $k'_{NPQ}/k^{Closed}_{NPQ}$ and c) $k^{Open}_{NPQ}/k^{Closed}_{NPQ}$ for each value of k^*_p/k_p. Assumptions: ^{PSII}RC induced NPQ rate switching is present (in these simulations $k^{Open}_{NPQ}/k^{Closed}_{NPQ} = 0.4$), no state transitions or chloroplast movements occur and the induction of NPQ is homogenous.

These simulations reveal that if the F' fluorescence yield parameter is measured with a larger portion of closed ^{PSII}RCs, the correlation intercept decreases while the gradient of the correlation increases. The decrease in the intercept is thought to be consistent with the sequential closure of the ^{PSII}RCs in the calculations, as this decreases the size of PQ^* leading to a lower intercept (outlined in Fig. S8). This decrease in the intercept can be used to obtain the k^*_p/k_p ratio by comparing $PQ^*(= k^*_p/(k_f + k_d))$ to PQ (its dark-adapted counterpart, $PQ = (F_m - F_o)/F_o = k_p/(k_f + k_d)$):

$$\frac{PQ^*}{PQ} = \frac{k^*_p}{(k_f + k_d)}\left(\frac{(k_f + k_d)}{k_p}\right) = \frac{k^*_p}{k_p} \quad (19)$$

From the experimental results (insert, Fig.4.(b)) this can be rewritten as:

$$\frac{PQ^*}{PQ} = \frac{k^*_p}{k_p} \approx qP \quad (20)$$

As more of ^{PSII}RCs were closed during the calculation of F' the gradient of the correlation ($m' = k'_{NPQ}/k^{Closed}_{NPQ}$) increases towards unity (Fig.S17.(b)), this indicates that the value of k'_{NPQ} starts to approach k^{Closed}_{NPQ} as more ^{PSII}RCs remain closed (⇒ lower qP). The linear nature of this change in gradient with decreasing qP values suggests that k'_{NPQ} is a weighted average between k^{Open}_{NPQ} and k^{Closed}_{NPQ} (⇒ $k'_{NPQ} = (qP)k^{Open}_{NPQ} + (1 - qP)k^{Closed}_{NPQ}$).

$$m' = \frac{k'_{NPQ}}{k^{Closed}_{NPQ}} \quad (21)$$

Since k'_{NPQ} is a weighted average based on the contribution from open and closed ^{PSII}RCs it can be re-expressed as $k'_{NPQ} = (qP)k_{NPQ}^{Open} + (1 - qP)k_{NPQ}^{Closed}$, using this expression equation (21) becomes:

$$m' = \frac{(qP)k_{NPQ}^{Open} + (1 - qP)k_{NPQ}^{Closed}}{k_{NPQ}^{Closed}} \quad (22)$$

$$\Rightarrow m' = qP\frac{k_{NPQ}^{Open}}{k_{NPQ}^{Closed}} + (1 - qP) \quad (23)$$

This can be rearranged to obtain an expression for $\frac{k_{NPQ}^{Open}}{k_{NPQ}^{Closed}}$:

$$\frac{k_{NPQ}^{Open}}{k_{NPQ}^{Closed}} = \frac{m' - (1 - qP)}{qP}, \text{where } qP \cong \frac{PQ^*}{PQ} \quad (24)$$

where m' is the gradient of the CQ vs NPQ^{Closed} correlation and qP is a proxy for the portion of open ^{PSII}RCs. Using equation (24), and the fact that qP can be calculated from the intercept of the correlation (using equation 20), values of $k_{NPQ}^{Open}/k_{NPQ}^{Closed}$ can be calculated from each of the simulated correlations. This yields values which are consistent with the input $k_{NPQ}^{Open}/k_{NPQ}^{Closed}$ value within error (Fig. S18.(c)). Additionally, the result of these calculations show that it is best to ensure that F' is measured while as many ^{PSII}RCs are open as possible as this minimises the error associated with the determined $k_{NPQ}^{Open}/k_{NPQ}^{Closed}$ value.

Notes S4: Ultrafast Fluorescence Spectra Deconvolution Procedure

S4.1 – ICA-guided deconvolution (F_m, F'_m, F_o and F'_o spectra)

Independent component analysis (ICA) is a multivariate analysis technique which can separate a dataset into a set of independent components (IC) utilising its higher order statistical moments (variance (2nd moment), skewness (3rd moment), kurtosis (4th moment), *etc*.).[40] Since ICA utilises the 3rd and 4th moments it will approximately separate the spectral slices of the ultrafast fluorescence spectrum into gaussian and non-gaussian components. Applying this analysis to the ultrafast fluorescence spectra is found to yield two distinct normalised spectral ICs (Fig.S19.(a)). Here, IC1 has a distinctly asymmetric and non-gaussian profile whereas IC2 has a much more gaussian line shape. The associated IC coefficients describe the contribution of each of these components to the emission spectrum over time, and are noted to yield a decay curve for each IC (Fig.S19.(b)).

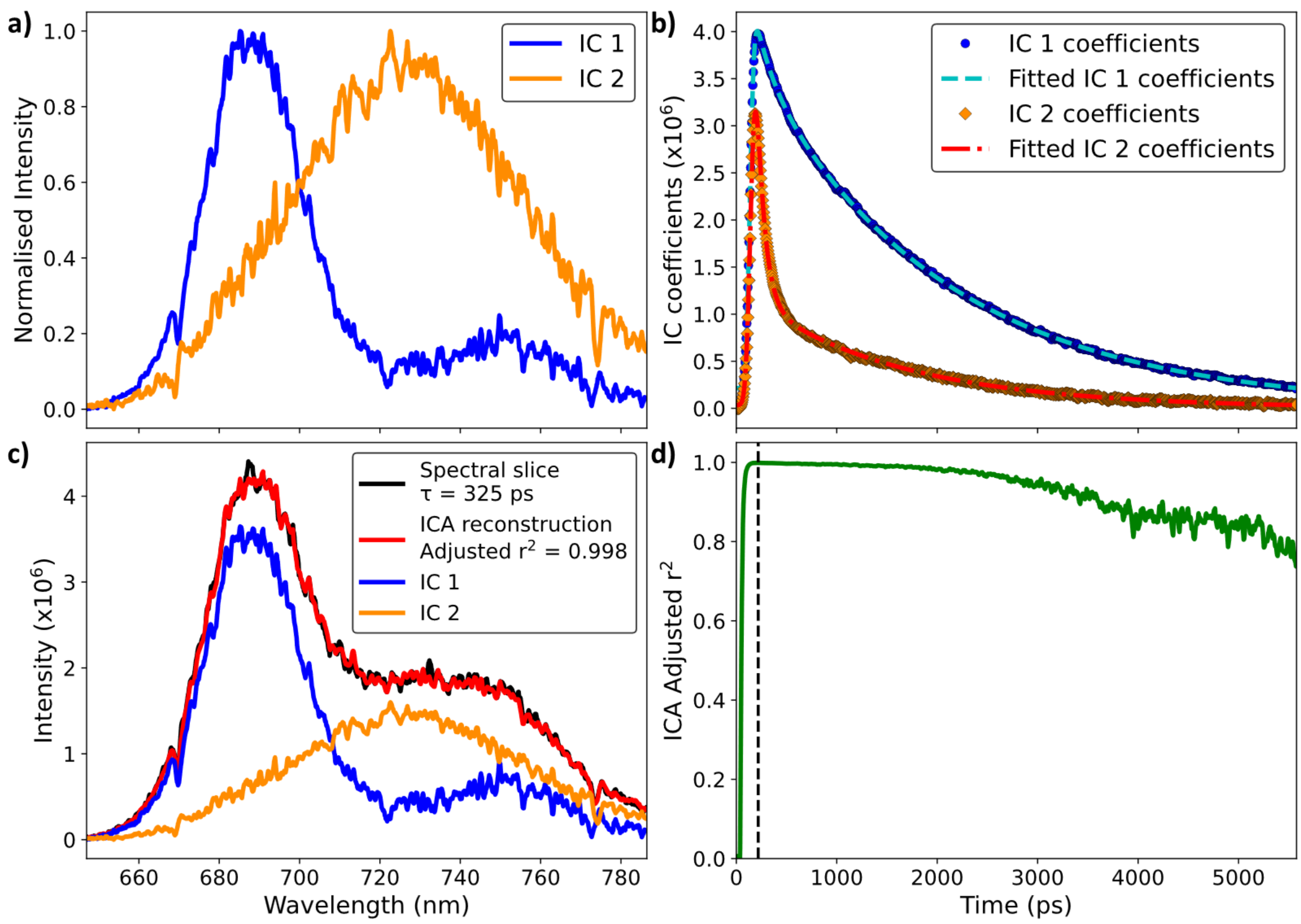

Figure S19: a) The normalised identified independent components (ICs) and b) the fitted associated coefficients, obtained upon applying ICA to a wt *A. thaliana* F_m-measurement. c) A representative ICA reconstruction of the spectral slice at $\tau = 325\ ps$ and d) the adjusted r^2 values of the ICA reconstructions as a function of time (dotted line indicating the arrival time of the excitation pulse (τ_o)).

The ICA reconstructions are able to accurately capture the changes in the spectra over time as a sum of the changes in the gaussian and non-gaussian profiles of the spectrum. At all times after the arrival time of the excitation pulse (τ_o) the ICA reconstruction of the data yields high adjusted r^2 values (>0.8, Fig.S19.(d)). It is noted that these values decrease with increasing time but this is thought to be due to noise. However, the overall purpose of this deconvolution of the spectral data is to extract the spectral and temporal contributions of PSI and PSII. Since the lifetime ranges of PSI (τ_{PSI} < 100 ps) and PSII (100 ps < τ_{PSII} < 2000 ps) are known,[44,55–60] it is possible to use this fact to obtain the deconvoluted PSII and PSI spectra as well as their associated decays from the ICA results (Fig.S19). The first step in this process involves fitting the IC coefficients (Fig.S19.(b)). The commented Python3.9 script used in this fitting process is outlined in figure S20.

```python
def fit_extracted_decay(cur_comp,user_cutoff,user_t):
    # This function fits normalised decays with 1,2 and 3 lifetime components and selects the best fit. All of the fit information is then returned to the user.
    to = list(cur_comp).index(max(cur_comp)) # The peak of the decay curve is found allowing the start of the decay potion of the curve to be isolated.
    fit_dict = {} # A dictionary is initialised to contain the fitting results of the exponential, biexponential and triexponential decay curves.
    try: # Here the data is fitted using an exponential decay curve.
        fit_1 = curve_fit(expdec,user_t[to:]-user_t[to],cur_comp[to:], bounds=((0,0),(np.inf,np.inf))) # The decay portion of the curve is fitted using a exponential decay to obtain intial inputs.
        guess_params_1 = [*fit_1[0],user_t[to],20] # The initial inputs (from the fit above), IRF peak position guess and IRF width guess (used in the fit of the full decay curve).
        IRF_fit_1 = curve_fit(IRF_expdec,user_t,cur_comp,p0=guess_params_1,bounds=((0,0,0,0),(np.inf,np.inf,np.inf,np.inf))) # The full decay curve is fitted with a convolution of the IRF and an exponential function.
        error_IRF_1 = get_stderror(*IRF_fit_1) # The associated errors are calculated.
        ave_lifetime_1 = IRF_fit_1[0][0] # The average lifetime is calculated.
    except RuntimeError: # If the fit fails a large error is returned to signify this error.
        IRF_fit_1 = [[1]]
        error_IRF_1 = [1000000]
        ave_lifetime_1 = -1
    try: # Here the data is fitted using an biexponential decay curve in a same manner as above.
        fit_2 = curve_fit(biexpdec,user_t[to:]-user_t[to],cur_comp[to:], bounds=((0,0,0,0),(np.inf,np.inf,np.inf,np.inf)))
        guess_params_2 = [*fit_2[0],user_t[to],20]
        IRF_fit_2 = curve_fit(IRF_biexpdec,user_t,cur_comp,p0=guess_params_2,bounds=((0,0,0,0,0,0),(np.inf,np.inf,np.inf,np.inf,np.inf,np.inf)))
        error_IRF_2 = get_stderror(*IRF_fit_2)
        ave_lifetime_2 = ((IRF_fit_2[0][0]*IRF_fit_2[0][2])+(IRF_fit_2[0][1]*IRF_fit_2[0][3]))/(IRF_fit_2[0][2]+IRF_fit_2[0][3])
        if len([x for x in IRF_fit_2[0][:2] if x<30]+[x for x in IRF_fit_2[0][:2] if x>5000])!=0: # If the fit returns non-physical lifetimes for chl fluorescence, a large error is returned to signify this error.
            IRF_fit_2 = [[1]]
            error_IRF_2 = [1000000]
            ave_lifetime_2 = -1
    except RuntimeError: # If the fit fails a large error is returned to signify this error.
        IRF_fit_2 = [[1]]
        error_IRF_2 = [1000000]
        ave_lifetime_2 = -1
    try: # Here the data is fitted using an triexponential decay curve in a same manner as above.
        fit_3 = curve_fit(triexpdec,user_t[to:]-user_t[to],cur_comp[to:], bounds=((0,0,0,0,0,0),(np.inf,np.inf,np.inf,np.inf,np.inf,np.inf)))
        guess_params_3 = [*fit_3[0],user_t[to],20]
        IRF_fit_3 = curve_fit(IRF_triexpdec,user_t,cur_comp,p0=guess_params_3,bounds=((0,0,0,0,0,0,0,0),(np.inf,np.inf,np.inf,np.inf,np.inf,np.inf,np.inf,np.inf)))
        error_IRF_3 = get_stderror(*IRF_fit_3)
        ave_lifetime_3 = ((IRF_fit_3[0][0]*IRF_fit_3[0][3])+(IRF_fit_3[0][1]*IRF_fit_3[0][4])+(IRF_fit_3[0][2]*IRF_fit_3[0][5]))/(IRF_fit_3[0][3]+IRF_fit_3[0][4]+IRF_fit_3[0][5])
        if len([x for x in IRF_fit_3[0][:3] if x<30]+[x for x in IRF_fit_3[0][:3] if x>5000])!=0: # If the fit returns non-physical lifetimes for chl fluorescence, a large error is returned to signify this error.
            IRF_fit_3 = [[1]]
            error_IRF_3 = [1000000]
            ave_lifetime_3 = -1
    except RuntimeError: # If the fit fails a large error is returned to signify this error.
        IRF_fit_3 = [[1]]
        error_IRF_3 = [1000000]
        ave_lifetime_3 = -1
    # The fitting dictionary is updated with the fitting results of the exponential, biexponential and triexponential decay curves.
    fit_dict.update({0:[IRF_expdec,IRF_fit_1,error_IRF_1,['t1','A','mu','sigma'],ave_lifetime_1]})
    fit_dict.update({1:[IRF_biexpdec,IRF_fit_2,error_IRF_2,['t1','t2','A','B','mu','sigma'],ave_lifetime_2]})
    fit_dict.update({2:[IRF_triexpdec,IRF_fit_3,error_IRF_3,['t1','t2','t3','A','B','C','mu','sigma'],ave_lifetime_3]})
    # The overall percentage error is used to calculated and the best fit is selected based on this percentage, here the maximum allowed percentage error is set to 10%.
    IRF_selector = [sum(np.asarray(error_IRF_1)/(len(IRF_fit_1[0])*IRF_fit_1[0])),sum(np.asarray(error_IRF_2)/(len(IRF_fit_2[0])*IRF_fit_2[0])),sum(np.asarray(error_IRF_3)/(len(IRF_fit_3[0])*IRF_fit_3[0]))]
    selected_fit = len([x for x in IRF_selector if x<=user_cutoff])-1
    return fit_dict[selected_fit]
```

Figure S20: Commented Python3.9 script for the automated fitting of exponential decay curves.

Here the IC coefficients are fitted using exponential, biexponential and triexponential decay curves. These curves were generated by convoluting the exponential decay functions with the instrument response function (IRF), which for the streak camera is generally approximated as a gaussian.[61] The script fits all three of the decay curves to the supplied data and calculates the associated errors. These errors and the fit parameters are then used to calculate the overall percentage error ($\sum_i 100\Delta p_i/p_i$, where p_i is the fit parameter and Δp_i is its associated error). The script then selects the fit with the most lifetime components which has an overall percentage error of below 10% (this can be altered by the user and depends on the signal-to-noise ratio of the measurement). Once all of the IC coefficient curves have been fit the obtained lifetimes are sorted into PSI and PSII (all lifetimes <100 ps are assigned as PSI and all lifetimes >100ps are assigned as PSII). Following this the PSI and PSII decays are calculated at each wavelength in the ultrafast spectrum (using the decays and their associated ICs). Once these decays have been generated they are fitted to each decay in the ultrafast spectrum allowing the PSI and PSII spectra to be extracted. If the user has indicated that there is background steady-state illumination present during the measurement this is taken into account during this final fitting procedure by adding a constant offset to the fitting of each decay. A summary of the results of this final state in the ICA-guided deconvolution are shown for a wt *A. thaliana* F_m-measurement is shown in figure S21.

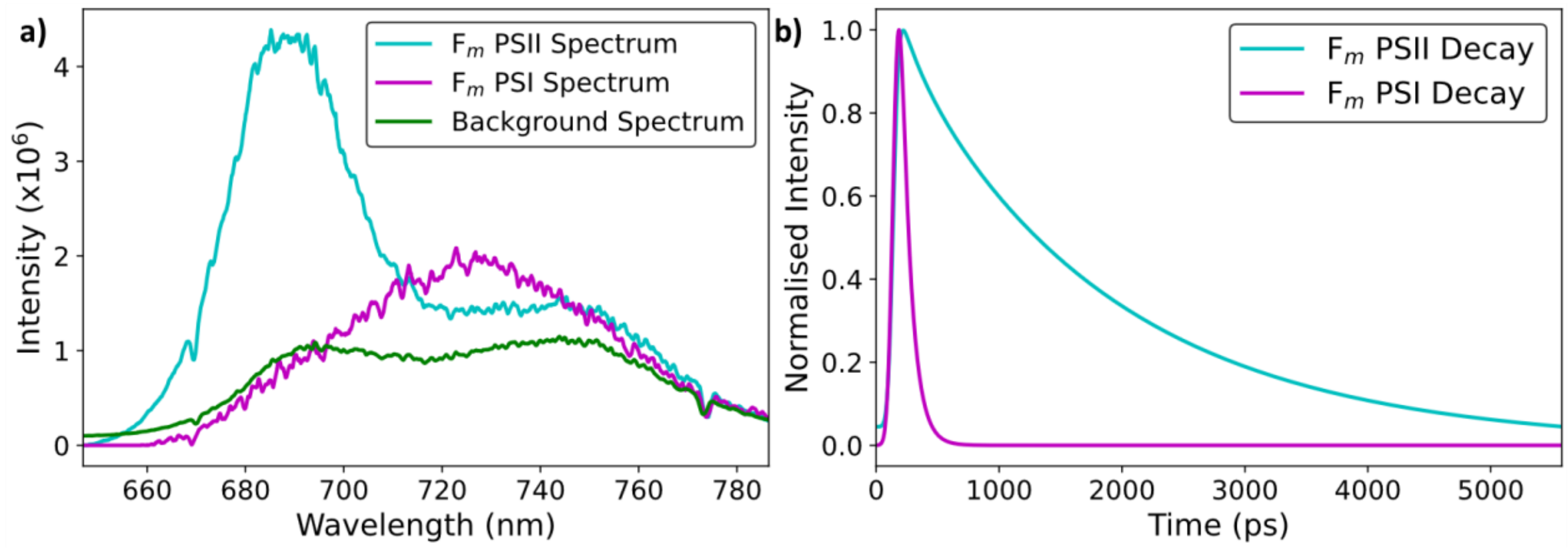

Figure S21: a) The deconvoluted PSII, PSI and background spectra and b) the normalised PSII and PSI decays obtained upon the ICA-guided deconvolution of the wt *A. thaliana* F_m-measurement.

Since the CW laser is used to ensure that all the ^{PSII}RCs were closed during this measurement, the deconvolution has extracted both the PSI and PSII spectra (Fig.S21.(a)), with their associated decays (Fig.S21.(b)), as well as the steady-state background spectrum (arising from the continuous illumination by the CW laser). Both the spectral and temporal aspects of the deconvoluted PSI and PSII spectra are consistent with previous ultrafast spectroscopy measurements (Fig.S21).[31] A Comparison of this deconvolution and the overall measured spectra is shown in figure S22.

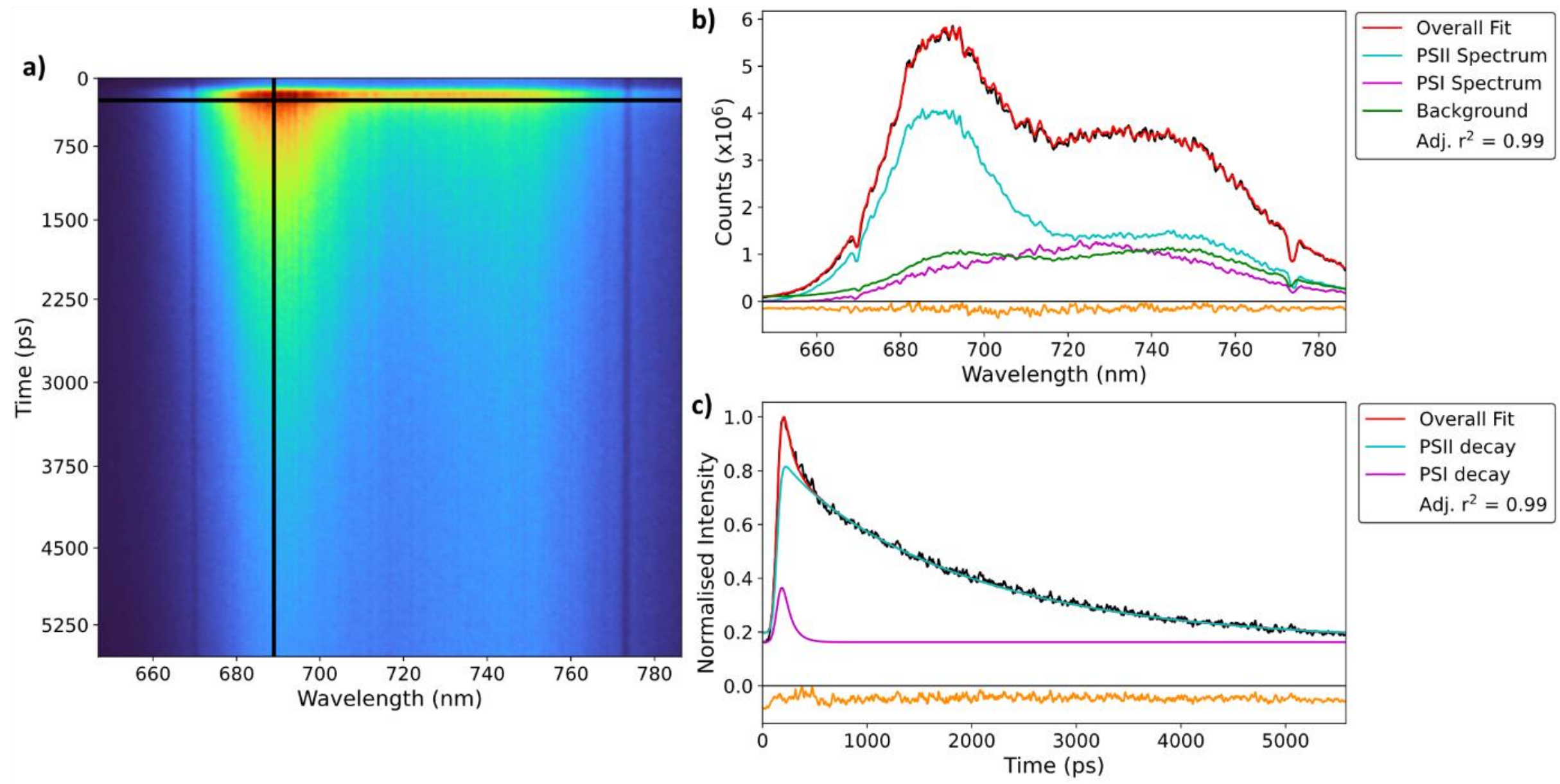

Figure S22: a) The wt *A. thaliana* measured F_m ultrafast fluorescence spectrum, b) a representative fit of the spectral slice at $\tau = 250\ ps$ (offset residual shown in orange) and c) a representative fit of the decay at $\lambda = 690\ nm$ (offset residual shown in orange), obtained *via* the ICA-guided deconvolution procedure.

Representative spectral ($\lambda = 690\ nm$, Fig. S.22.(b)) and temporal ($\tau = 250\ ps$, Fig. S.22.(c)) slices show a direct comparison between the measured data (black, Figs. S22.(b)&(c)) and the overall fit (red, Figs. S22.(b)&(c)) obtained from the ICA-guided deconvolution procedure. These overall fits show excellent agreement with the experimental spectrum (adjusted r^2 = 0.99, for both the spectral and temporal slices) and the fit residuals are also noted to be small and randomly distributed (dark orange, offset Fig. S22.(b)&(c)) indicating that these components fully capture the experimental data.

S4.2 – Steady state fluorescence backgrounds (F_m and F'_m spectra)

The F_m and F'_m spectra are measured in the presence of a steady state light source. For the F_m spectra this steady state light source is the CW laser used to ensure that all the ^{PSII}RCs are closed whereas for the F'_m spectra this steady state light source is the combination of the CW laser and the actinic light used to induce NPQ. These steady state fluorescence backgrounds are summarised in figure S23:

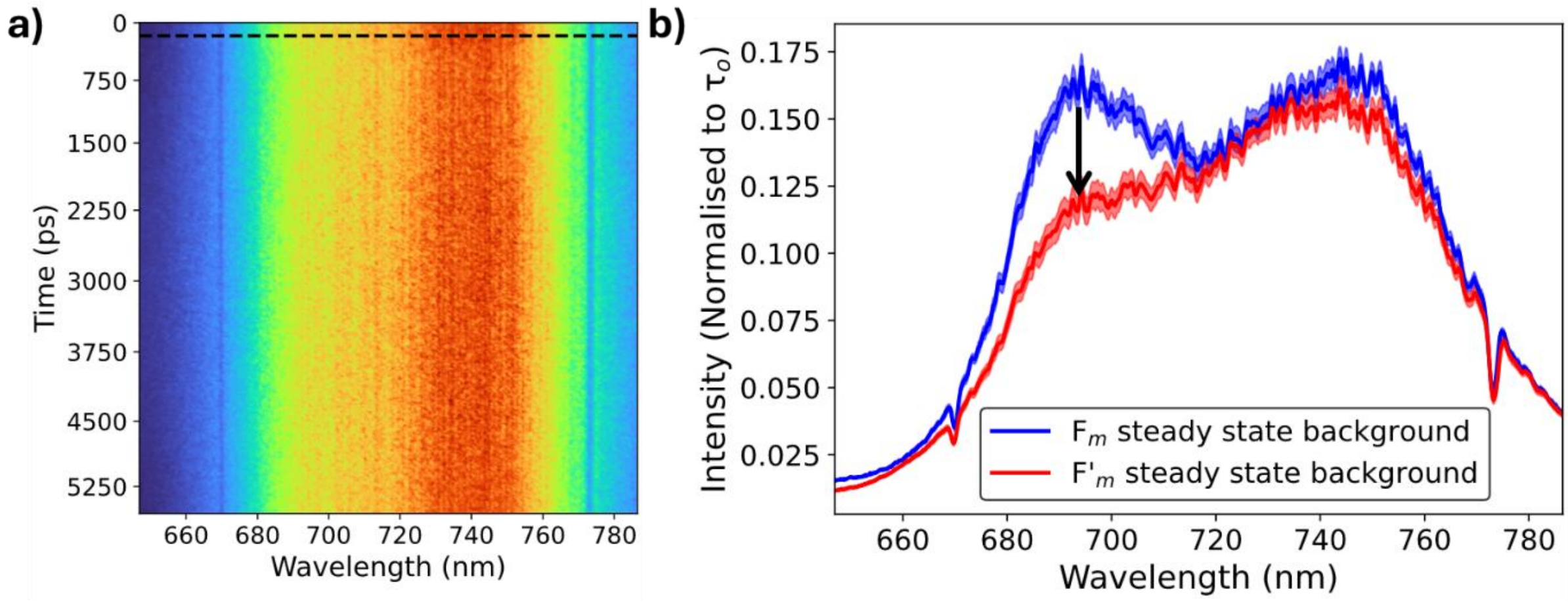

***Figure S23*:** Representative steady state background fluorescence spectra of wt *A. thaliana* under a) F'_m conditions (without ultrafast laser, τ_o indicated by dashed black line) and b) the average (n = 3) F_m (blue) and F'_m (red) steady state background fluorescence spectra of wt *A. thaliana* (the effect of NPQ is indicated by a black arrow). Shaded area shows the associated standard error.

A representative steady state background fluorescence spectrum measured following NPQ induction, without the ultrafast laser pulses, is shown for wt *A. thaliana* in the F'_m state in figure S23.(a). This spectrum reveals that this fluorescence signal is constant over time and always present, even before τ_o (the arrival time of the ultrafast laser pulse when this is not blocked). This reveals that this steady state background is not part of the ultrafast fluorescence signal induced by the ultrafast laser pulse. Furthermore, the steady state background spectra are very consistent across all the replicas (n = 3) for both the F_m and F'_m measurements (Fig.S23.(b)) and appear to be

mixtures of the steady state PSII and PSI spectra suggesting that these are due to the illumination of the leaves with constant light sources. This is also supported by the spectral differences between the steady state backgrounds seen for F_m and F_m' which are consistent with the quenching of the PSII dominated part of the spectrum due to the presence of NPQ (black arrow, Fig.S23.(b)).

S4.3 – F_m-guided ICA deconvolution (NPQ induction low SNR F_m' spectra)

The F_m'-spectra measured during the 20 minute NPQ inductions are harder to analyse due to the lower SNR inherent in these measurements. In order to reliably deconvolute each of these individual spectra an F_m-guided version of the ICA deconvolution procedure was developed. In this F_m-guided variant, ICA is applied to the high SNR F_m-spectrum (measured at the start of the experiment, as outline in the Methods) to obtain the non-gaussian and gaussian ICs (outlined in S4.1, Fig. S19.(a)). These ICs are then used to fit each of the low SNR F_m'-spectra, allowing the decays associated with the non-gaussian and gaussian parts of the spectral line shape to be extracted for each of the low SNR F_m'-spectra. Following this, these decays are fitted and the obtained lifetimes are used to reconstruct both the PSI and PSII spectra as well as their associated decays for each of the F_m'-spectra in the sequence (as outlined in S4.1). This allows the average PSI and PSII lifetimes to be extracted during the NPQ induction period and NPQ^{Closed} to be calculated using the temporal version of the Stern-Volmer equation ($NPQ^{Closed} = \left(\langle\tau_{F_m}^{PSII}\rangle - \langle\tau_{F_m'}^{PSII}\rangle\right)/\langle\tau_{F_m'}^{PSII}\rangle = k_{NPQ}^{Closed}/\left(k_f + k_d\right)$, Fig. 1).

Notes S5: Discrepancy in Φ_{PSII} due to NPQ rate-switching

The quantum yield of PSII (Φ_{PSII}) is an important photosynthetic parameter which is widely used throughout photosynthetic research. Theoretically this parameter ($\Phi_{PSII}^{Theoretical}$) is defined as:

$$\Phi_{PSII}^{Theoretical} = \frac{k_p^*}{(k_f + k_d + k_p^* + k_{NPQ})} \quad (25)$$

In PAM fluorometry this is often calculated from the fluorescence yield using the following equation (derived using the Butler model):

$$\Phi_{PSII} = \frac{F_m' - F'}{F_m'} \quad (26)$$

where F' is the instantaneous fluorescence just prior to the application of a saturating pulse (equation (5a)) and F_m' is the maximum fluorescence obtained upon the application of the saturating pulse (equation (5a)). If the quenching rate of NPQ is assumed to be unaffected by the

state of the ^{PSII}RC, it can be shown that equations (25) and (26) are equivalent to each other. However, the experimental results here show that this assumption does not hold. Using this result it can be shown that the equation (26) actually yields the following expression:

$$\Phi_{PSII}^{RC-switching} = \frac{F_m' - F'}{F_m'} = \frac{k_p^* + k_{NPQ}' - k_{NPQ}^{Closed}}{(k_f + k_d + k_p^* + k_{NPQ}')} \quad (27)$$

where k_{NPQ}^{Closed} is the quenching rate of NPQ associated with F_m', k_{NPQ}' is the quenching rate of NPQ associated with F' and $m' = k_{NPQ}'/k_{NPQ}^{Closed}$ (equation (21)). Using $k_{NPQ}' = m' k_{NPQ}^{Closed}$ equation (27) simplified to:

$$\Phi_{PSII}^{RC-switching} = \frac{F_m' - F'}{F_m'} = \frac{k_p^* + (m' - 1)k_{NPQ}^{Closed}}{(k_f + k_d + k_p^* + m' k_{NPQ}^{Closed})} \quad (28)$$

Interestingly, this suggests that equation (26) overestimates Φ_{PSII} if $m' > 1$ and underestimates Φ_{PSII} if $m' < 1$.

The size of this discrepancy is explored for wt *A. thaliana* by comparing the experimentally measured values of $(F_m' - F')/F_m'$ to the calculated values of $\Phi_{PSII}^{RC-switching}$ and $\Phi_{PSII}^{Theoretical}$. In order to calculate the corresponding values of $\Phi_{PSII}^{RC-switching}$ and $\Phi_{PSII}^{Theoretical}$ values of $(k_f + k_d)$, k_p^*, k_{NPQ}^{Closed} and m' were determined using the average F_m PSII lifetime (Fig. 2.(a)), the measured NPQ^{Closed} values and the results of the fluorescence yield correlations (Fig. 4). Specifically, $(k_f + k_d) = 1/\langle\tau_{F_m}^{PSII}\rangle$, $k_{NPQ}^{Closed} = NPQ^{Closed}/\langle\tau_{F_m}^{PSII}\rangle$, $k_p^* = PQ^*/\langle\tau_{F_m}^{PSII}\rangle$ (where PQ^* is the intercept of the correlation in Fig. 4.(b)) and m' is calculated *via* equation (23) (using the average gradient of the fluorescence yield correlations (Fig. 4) to obtain a value for $k_{NPQ}^{Open}/k_{NPQ}^{Closed}$). The results of these comparisons are summarised in figure S24.

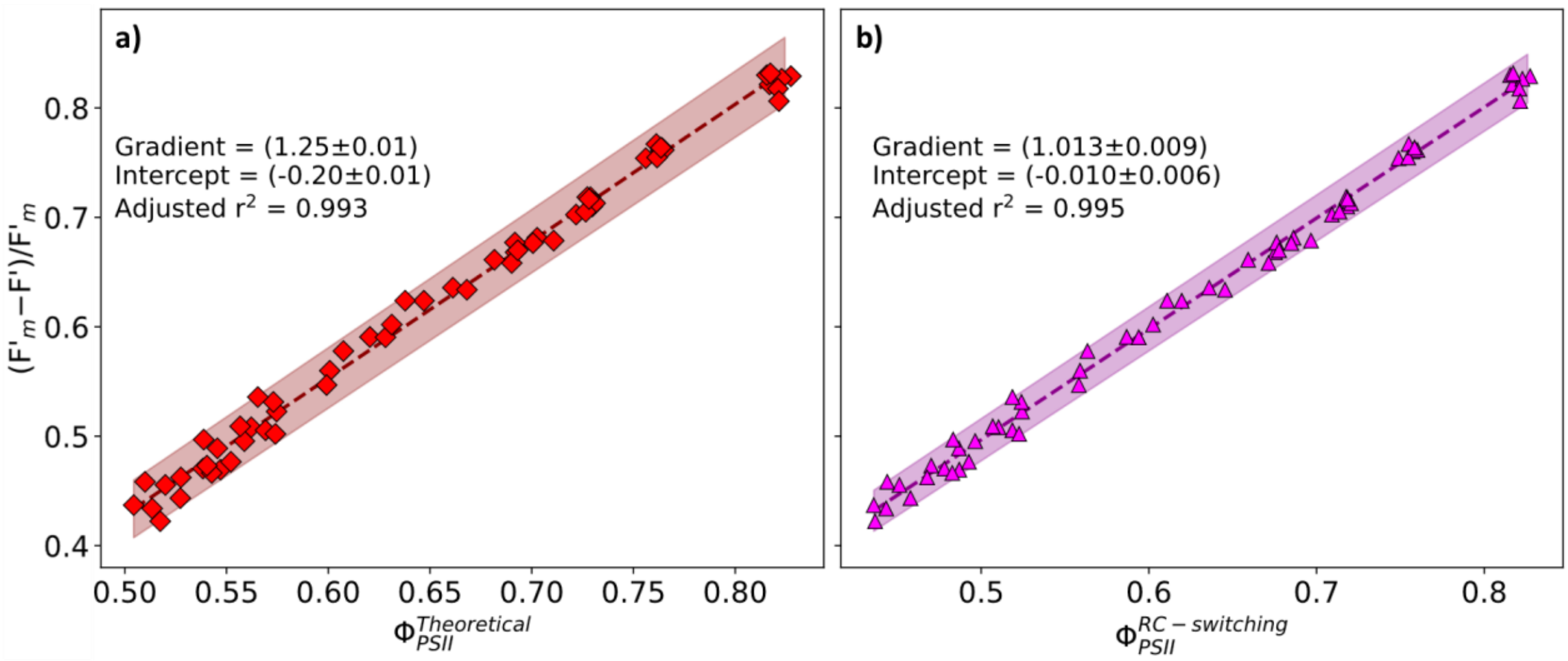

Figure S24: Fitted comparison between a) $(F_m' - F')/F_m'$ and $\Phi_{PSII}^{Theoretical}$, and b) $(F_m' - F')/F_m'$ and $\Phi_{PSII}^{RC-switching}$ for wt *A. thaliana*.

These comparisons reveal that $(F_m' - F')/F_m' < \Phi_{PSII}^{Theoretical}$ (Fig. S24.(a)), yielding strong linear correlation with a gradient of (1.25±0.01) and an intercept of (-0.20±0.01). This is consistent with the result from the fluorescence lifetime measurements (Figs. 2&3) and yield correlations (Fig. 4) which show $k_{NPQ}^{Open} = 0.65k_{NPQ}^{Closed}$ ($\Rightarrow m' < 1$). The comparison between $(F_m' - F')/F_m'$ and $\Phi_{PSII}^{RC-switching}$ (Fig. S24.(b)) reveals that these parameters are strongly linearly correlated yielding a gradient of (1.013±0.009) and an intercept of (-0.010±0.006). This shows that the experimentally obtained $(F_m' - F')/F_m'$ values can only be explained by the inclusion of RC-induced NPQ rate switching. Overall, this discrepancy reveals that equation (26) always underestimates Φ_{PSII} and this underestimation gets progressively worse as NPQ is induced:

$$\Phi_{PSII}^{Theoretical} = n(NPQ)\left\{\frac{F_m' - F'}{F_m'}\right\} \quad (29)$$

where $n(NPQ)$ is an NPQ dependent scaling factor which accounts for the underestimation of Φ_{PSII} in equation (26).

***Table S2*:** Comparison between $\Phi_{PSII}^{Theoretical}$ and $(F_m' - F')/F_m'$ for different values of NPQ, for wt *A. thaliana*.

NPQ	$\Phi_{PSII}^{Theoretical}$	$\frac{F'_m - F'}{F'_m}$	$n(NPQ)$
0.42±0.02	0.76±0.01	0.76±0.01	1.00±0.01
1.00±0.03	0.70±0.01	0.67±0.02	1.04±0.01
1.60±0.07	0.64±0.01	0.61±0.01	1.05±0.02
2.39±0.06	0.57±0.01	0.52±0.01	1.10±0.02
2.76±0.06	0.55±0.01	0.49±0.01	1.13±0.02
3.17±0.05	0.52±0.01	0.45±0.01	1.17±0.02

Notes S6: Additional NPQ (ΔNPQ) in closed ^{PSII}RC due to Chl_{D2} triplet formation

With a rather simple approach the rate of Chl_{D2} triplet formation in closed ^{PSII}RCs can be estimated as follows. We assume that the rate of initial charge separation for closed ^{PSII}RCs is the same as for open ^{PSII}RCs but for open ^{PSII}RCs charge separation is followed by the formation of reduced Q_A whereas in closed RCs triplets can be formed with a quantum yield of 10-20%.[52] The trapping rate for open ^{PSII}RCs can be calculated from the obtained fluorescence lifetimes for dark-adapted

open and closed ^{PSII}RCs, being 344 and 1756 ps (see Results) according to 1/0.344 – 1/1.756 = 2.91 – 0.57 = 2.34 ns^{-1}. The rate of triplet formation, being 10-20% of this value, is then 2.34 * (0.1-0.2) = 0.234-0.468 ns^{-1}. The resulting size of this additional NPQ (ΔNPQ) is subsequently obtained by dividing these values by the inverse lifetime of dark-adapted closed RCs (1.756 ns) which gives ΔNPQ = 0.41-0.82.
