# Supplementary material for: The state of plant photosystem II reaction centres affects the rate of non-photochemical quenching
Source: Nat Plants. 2026 Aug 14;12(8):1637–46. doi: 10.1038/s41477-026-02357-x (PMC13481247; doi:10.1038/s41477-026-02357-x)
Supplement: Supplementary file 3 [file 41477_2026_2357_MOESM3_ESM.pdf]

nature portfolio

Peer Review File

The State of Plant Photosystem II Reaction Centres affects the rate of Non-Photochemical Quenching

Corresponding Author: Professor Herbert van Amerongen

Version 0:

Reviewer comments:

Reviewer #1

(Comments for the Author)
This paper addresses a crucial aspect of the regulation of photosynthetic light utilization. The dissipation of excess light energy as heat (NPQ) is known to serve as important photoprotective mechanism under high light stress, but NPQ is also supposed to limit photosynthetic efficiency under fluctuating light, in particular after high light to low light transitions. The detailed mechanistic understanding of NPQ is thus of high interest for the possible improvement of crop yield by optimizing the NPQ dynamics.
NPQ is typically determined for closed reaction centers (RC) of PSII, and hence in absence of photochemistry, which, however, is not the typical situation in the field. Indeed, earlier work proposed that NPQ is more efficient for closed RCs compared to open PSII RCs. In the present work, the authors addressed this important point and determined the NPQ rates for open and closed PS RCs. Applying both chlorophyll fluorescence lifetime and fluorescence yield measurements, it is clearly shown that the NPQ for open RCs is about 35% lower than for closed RCs. The authors conclude from these data that the NPQ quenching rate is switched in response to the state of the RC, and consequently, that the potential improvement of photosynthetic efficiency by manipulation of the NPQ dynamics is 35% less than originally estimated.
The experimental approach is well-chosen and the paper is written in a very clear and concise way. The conclusions made by the authors are fully supported by the data. However, in my view, it is essential to include mutants that are known to affect specific components contributing to NPQ. In particular, stt7 mutants should be included to clarify unambiguously, to what extent state transitions might contribute to the observed differences between the NPQ rates in open and closed RCs. Moreover, PsbS over-expressing mutants (e.g. L17, Li et al 2002) and the xanthophyll cycle mutant npq1 should be included as well, to determine whether or not PsbS- and/or Zea-dependent NPQ is affected. The experimental effort for such measurements seems to be reasonable, but would provide valuable information about the impact of PsbS and Zea on NPQ in open PSII RCs.
Other points:
In Figure 4, the maximum NPQ values for closed RCs of about 3.5 are quite high and typically found for Arabidopsis only in PsbS over-expressing lines (e.g. Li et al, 2004). It can thus not be excluded that photoinhibitory quenching (qI) contributes to NPQ at the highest light intensities. I wonder, whether this high NPQ is fully reversible upon relaxation of the pH gradient (this could be easily tested at the end of the light response curves). In this context, the authors should also discuss the consequences, if pH-independent NPQ processes, such as qZ or qI, are active under the experimental conditions.
The measurements for the light response curves are rather long (70 min in total). The authors should specify the physiological conditions during those experiments. Were intact leaves attached to the plant used or detached leaves or even leaf discs? Did the setup allow control of the temperature and gas exchange, specifically CO_2 availability?

(Remarks on code availability)

Reviewer #2

(Comments for the Author)
The provided manuscript, entitled “Rates of Non-Photochemical Quenching in plants are partially determined by the state of Photosystem II Reaction Centres”, addresses the question on how the quenching rates of non-photochemical quenching (NPQ) are influenced by the photochemical quenching (PQ) of the open reaction center. Since in nature, most of the time both photochemical and non-photochemical quenching are present simultaneously, it is of interest to explore quenching rates in the fully open and intermediate RC conditions, besides the well documented closed condition. To gain insights, experiments were performed on a self-made ultrafast fluorescence lifetime setup and on a commercial PAM fluorometer to collect time-resolved spectra and steady state fluorescence intensities respectively. Experiments were performed on dark and light adapted leaves of wt A. Thaliana.

In the results section, the fluorescence emission spectra of PSI and PSII of the fully closed and fully open RCs, obtained from applying ICA to the ultrafast fluorescence, are in good agreement to others. Also, the lifetimes of PSI and PSII, the decrease in PSII lifetime upon illumination, the absence of PSI NPQ adaptation and the overall NPQ fits well into literature. One opportunity that I think is missed out here, is a comparison of the intensities of PSI and PSII relative to each other, since all spectra are normalised to 1. I have a paper in mind where this was achieved for dark adapted plants by using excitation and detection conditions specific for PSI (F. Franck et al. / Biochimica et Biophysica Acta 1556 (2002) 239–246), instead of a data analysis approach. It would be interesting to see how much the PSI and PSII fluorescence spectra account to the total F'O and F'm steady state spectra. I imagine something similar to the spectrum in figure S14 c), but not for a temporal slice but for the full integration over the measurement time with F'O and F'm conditions.

A small comment in addition: I expected to see some actual time resolved spectra the "Ultrafast Fluorescence" sections of the main paper, besides the steady state spectra of PSI and PSII. I understand that these measurements are not new to the community and can be found for example in earlier publications of your group, such as Farooq et al. 2018, but for me it would increase the trust in the time constants found.

Later on, the manuscript contains two modified versions of the commonly used Stern-Volmer equation, for calculation the NPQ value of open and partially open/closed RCs, which were derived in the supporting information, and could be of potential value for others. One thing I'm worried about here is the use of the equation $NPQ^{Open} = k\_NPQ^{Open} / (kf + kd)$ in line 700. What is the reference for it and why is it not $NPQ^{Open} = k\_NPQ^{Open} / (kf + kd + kp)$? Based on the equation $NPQ^{Closed} = \_NPQ^{Closed} / (kf + kd)$ and comparing it with the equations for Fm and FO it seems wrong not to consider the rate of photochemical quenching kp. As a result, the derived Stern Volmer equation for open RC would be reduced to $NPQ^{Open} =$ (FO-F'O)/F'O, having great impact on the result of the paper.

For the general idea, it makes sense to me, that the "yield" of NPQ decreases, going from closed to open RC, since the open RCs provide an additional energy pathway besides the NPQ dissipation channels, but for me it is not said that also the rate of NPQ (k_NPQ) itself changes. The idea of RC-induced switching of the NPQ rate seems like a good explanation for the results and the underlying mechanisms how this is achieved in nature holds great potential for further research, but I still want to emphasize my above-mentioned concerns on the influence of kp on the NPQ^Open rate.

In summary the manuscript is worked out very well and it gives a great view on the quenching rates of open reaction centers. The consequences in the difference of the open and closed NPQ rate constants are indeed important to consider, for example in the estimated increase in crop yield due to NPQ optimization. Since in nature the reaction centers are always distributed between open and closed, the reduction of the possible yield increase due to NPQ optimization, from 30% to 20% estimated by the authors, may be a bit drastic but the overall message is right.

So, my recommendation is a revision. If the rate constant of the photochemical quenching kp is intentionally left out in the equation of NPQ^Open, I don't see any problem with publishing this manuscript. The comparison of the absolute intensities of PSI and PSII in open/closed RC under NPQ conditions would have been nice, but since it is not in the main focus of the paper, I understand that it is not explored further. Maybe it should be considered as a topic for a future manuscript.

(Remarks on code availability)

Reviewer #3

(Comments for the Author)
In this manuscript, the authors characterize a component of the set of photoprotective processes, NPQ, which serve a safety valve under high light in oxygenic photosynthesis. These processes have received a lot of interest over the past several years for the possibility that they can be fine-tuned for biomass gains.

Here, the authors build on their previous work (Ref 30, Nat Plants 2018), where they initially reported an NPQ component specific to the presence of closed PSII RCs, by quantifying this component and discussing its implications for biomass gains based on NPQ. Evaluation of this component is important to understanding NPQ, and so this is a worthwhile study. However, given the complexity of the system, isolating a single component is challenging. While the authors have carefully performed a detailed analysis, there are still aspects of the isolation that need additional clarification prior to publication.

Specifically:
1. In the current analysis, the model seems to assume no crosstalk between the NPQ rate and the photochemistry rate, i.e., the 'lake model.' However, as NPQ increases, the rate of NPQ can interfere with the apparent rate of photochemistry even without an additional NPQ component ascribed to the RC, i.e., the 'puddle model.' Prior to publication, the authors need to demonstrate clearly that it is not this type of cross talk that is giving rise to the observed effect.
2. The authors find a constant PSI lifetime, and ascribe the changes in NPQ to be exclusively due to PSII NPQ. How would PSI-NPQ impact these results?
3. The authors decompose their spectra into a PSI, PSII, and background component (e.g., S17b). However, the background component has a very high amplitude – approximately the same as PSI and about half of PSII. Is the background component consistent across all measurements? Could this be a mis-assigned mixture of PSI and PSII?
4. The extracted parameters, NPQ closed vs NPQ open, are highly processed. It is not clear to me what differences these

produce in the actual recorded data (i.e., what percent change in the intensity or lifetime of the fluorescence is due to this effect). This would aid in the evaluation of the likelihood that the extracted parameters would be impacted by some of the factors above.
5. As an editorial comment, the manuscript is extremely technical, making it challenging to read, particularly the actual analysis procedure. Including all the equations used is helpful, but it could be presented more clearly with additional explanation.

(Remarks on code availability)

Reviewer #4

(Comments for the Author)
Ramakers et al., reported an interesting observation that the open PSII has an instantaneous switch, leading to a quenching rate 35 % lower than that of closed PSII RC, suggesting that plants are more economic than we thought they were. They have essentially solved a long-standing puzzle in the field regarding the quenching rate of open RCs, which has been an experimental challenge for many years, as reduced PQ pool and NPQ are always coupled. And their results are convincing, at the same time, the discussion is provoking.

The approach taken is valid and reasonable; although the data are somewhat noisy, the use of statistics was appropriately applied, which alleviated most of my doubts. The text is well-written, very clear in general, and easy to follow.

A few comments before publishing:
1. It might not be the case that the PSII RC was completely reopen 1 second or 10 seconds after a certain time period of HL treatment. The reduction pathway of the PQ pool in darkness, e.g., electrons from FQR, might still be closing the RCs even without light; would that alter the conclusion to some extent?.
2. Besides the explanation of a faster NPQ rate of open RC provided by the authors, alternatively, could it be possible that the faster rate is simply because of a rapid de-protonation of PsbS or varied interactions between Chls and the quenching molecule/s that relax the quenching on a time-scale of seconds or even milliseconds?

Minor point:
Actinic light should be specified for both figures 1 and 4.

(Remarks on code availability)

Decision Letter:

24th October 2025

Dear Professor van Amerongen,

Your study, "Rates of Non-Photochemical Quenching in plants are partially determined by the state of Photosystem II Reaction Centres" has now been seen by four referees. You will see from their comments below that while they find your work of interest, some important points are raised. We remain keen to publish your study in Nature Plants, but would like to consider your response to these concerns in the form of a revised manuscript before we make a final decision on publication.

We therefore invite you to revise your manuscript taking into account all reviewer and editor comments. Please highlight all changes in the manuscript text file.

We are committed to providing a fair and constructive peer-review process. Do not hesitate to contact us if there are specific requests from the reviewers that you believe are technically impossible or unlikely to yield a meaningful outcome.

When revising your manuscript please:

* Include a "Response to referees" document detailing, point-by-point, how you addressed each referee comment. If no action was taken to address a point, you must provide a compelling argument. This response will be sent back to the referees along with the revised manuscript.

* If you have not done so already please begin to revise your manuscript so that it conforms to our Article format instructions at http://www.nature.com/nplants/info/final-submission. Refer also to any guidelines provided in this letter.

* Include a revised version of any required reporting checklist. It will be available to referees (and, potentially, statisticians) to aid in their evaluation if the manuscript goes back for peer review. A revised checklist is essential for re-review of the paper.

* Pay close attention to our href="https://www.nature.com/nature-portfolio/editorial-policies/image-integrity">Digital Image Integrity Guidelines. and to the following points. Please ensure:

-- that unprocessed scans are clearly labelled and match the gels and western blots presented in figures.
-- that control panels for gels and western blots are appropriately described as loading on sample processing controls
-- that all images in the paper are checked for duplication of panels and for splicing of gel lanes.
-- that you retain unprocessed data and metadata files after publication, ideally archiving data in perpetuity. These may be requested during the peer review and production process or after publication if any issues arise.

EXTENDED DATA FIGURES

When re-submitting your manuscript, please ensure that any supplementary figures and tables that are crucial to the manuscript's conclusions are converted into Extended Data figures and tables to increase visibility of these data. Extended Data figures and tables are online-only (present in the online PDF and full-text HTML versions of the paper), peer-reviewed display items that provide essential background to the article but are not included in the main article due to space constraints. A maximum of ten Extended Data display items (figures and tables) is permitted.

Nature Plants strongly supports public availability of data and are therefore keen that the data used in your paper is placed in an appropriate public data repository. Alternatively, if this is not possible, you may present the data as Extended Data or Supplementary Information. If data can only be shared on request, please explain why in your Data Availability Statement, and also in the correspondence with your editor. Please note that for some data types, deposition in a public repository is mandatory.

Please use the link below to submit your revised manuscript and related files:

Link Redacted

Note: This URL links to your confidential home page and associated information about manuscripts you may have submitted, or that you are reviewing for us. If you wish to forward this email to co-authors, please delete the link to your homepage.

We hope to receive your revised manuscript within four to eight weeks. If you cannot send it within this time, please let us know. We will be happy to consider your revision so long as nothing similar has been accepted for publication at Nature Plants or published elsewhere.

We look forward to seeing the revised manuscript and thank you for the opportunity to review your work.

****

Nature Plants is committed to improving transparency in authorship. As part of our efforts in this direction, we are now requesting that all authors identified as 'corresponding author' on published papers create and link their Open Researcher and Contributor Identifier (ORCID) with their account on the Manuscript Tracking System (MTS), prior to acceptance. This applies to primary research papers only. ORCID helps the scientific community achieve unambiguous attribution of all scholarly contributions. You can create and link your ORCID from the home page of the MTS by clicking on 'Modify my Springer Nature account'. For more information please visit please visit www.springernature.com/orcid.

Please do not hesitate to contact me if you have any questions or would like to discuss these revisions further.

****

Reviewers' Comments:

Reviewer #1 (Comments for the Author):

This paper addresses a crucial aspect of the regulation of photosynthetic light utilization. The dissipation of excess light energy as heat (NPQ) is known to serve as important photoprotective mechanism under high light stress, but NPQ is also supposed to limit photosynthetic efficiency under fluctuating light, in particular after high light to low light transitions. The detailed mechanistic understanding of NPQ is thus of high interest for the possible improvement of crop yield by optimizing the NPQ dynamics.
NPQ is typically determined for closed reaction centers (RC) of PSII, and hence in absence of photochemistry, which, however, is not the typical situation in the field. Indeed, earlier work proposed that NPQ is more efficient for closed RCs compared to open PSII RCs. In the present work, the authors addressed this important point and determined the NPQ rates for open and closed PS RCs. Applying both chlorophyll fluorescence lifetime and fluorescence yield measurements, it is clearly shown that the NPQ for open RCs is about 35% lower than for closed RCs. The authors conclude from these data that the NPQ quenching rate is switched in response to the state of the RC, and consequently, that the potential improvement of photosynthetic efficiency by manipulation of the NPQ dynamics is 35% less than originally estimated.
The experimental approach is well-chosen and the paper is written in a very clear and concise way. The conclusions made by the authors are fully supported by the data. However, in my view, it is essential to include mutants that are known to affect specific components contributing to NPQ. In particular, stt7 mutants should be included to clarify unambiguously, to what extent state transitions might contribute to the observed differences between the NPQ rates in open and closed RCs. Moreover, PsbS over-expressing mutants (e.g. L17, Li et al 2002) and the xanthophyll cycle mutant npq1 should be included

as well, to determine whether or not PsbS- and/or Zea-dependent NPQ is affected. The experimental effort for such measurements seems to be reasonable, but would provide valuable information about the impact of PsbS and Zea on NPQ in open PSII RCs.
Other points:
In Figure 4, the maximum NPQ values for closed RCs of about 3.5 are quite high and typically found for Arabidopsis only in PsbS over-expressing lines (e.g. Li et al, 2004). It can thus not be excluded that photoinhibitory quenching (qI) contributes to NPQ at the highest light intensities. I wonder, whether this high NPQ is fully reversible upon relaxation of the pH gradient (this could be easily tested at the end of the light response curves). In this context, the authors should also discuss the consequences, if pH-independent NPQ processes, such as qZ or qI, are active under the experimental conditions.
The measurements for the light response curves are rather long (70 min in total). The authors should specify the physiological conditions during those experiments. Were intact leaves attached to the plant used or detached leaves or even leaf discs? Did the setup allow control of the temperature and gas exchange, specifically CO2 availability?

Reviewer #2 (Comments for the Author):

The provided manuscript, entitled “Rates of Non-Photochemical Quenching in plants are partially determined by the state of Photosystem II Reaction Centres”, addresses the question on how the quenching rates of non-photochemical quenching (NPQ) are influenced by the photochemical quenching (PQ) of the open reaction center. Since in nature, most of the time both photochemical and non-photochemical quenching are present simultaneously, it is of interest to explore quenching rates in the fully open and intermediate RC conditions, besides the well documented closed condition. To gain insights, experiments were performed on a self-made ultrafast fluorescence lifetime setup and on a commercial PAM fluorometer to collect time-resolved spectra and steady state fluorescence intensities respectively. Experiments were performed on dark and light adapted leaves of wt A. Thaliana.

In the results section, the fluorescence emission spectra of PSI and PSII of the fully closed and fully open RCs, obtained from applying ICA to the ultrafast fluorescence, are in good agreement to others. Also, the lifetimes of PSI and PSII, the decrease in PSII lifetime upon illumination, the absence of PSI NPQ adaptation and the overall NPQ fits well into literature. One opportunity that I think is missed out here, is a comparison of the intensities of PSI and PSII relative to each other, since all spectra are normalised to 1. I have a paper in mind where this was achieved for dark adapted plants by using excitation and detection conditions specific for PSI (F. Franck et al. / Biochimica et Biophysica Acta 1556 (2002) 239–246), instead of a data analysis approach. It would be interesting to see how much the PSI and PSII fluorescence spectra account to the total F’O and F’m steady state spectra. I imagine something similar to the spectrum in figure S14 c), but not for a temporal slice but for the full integration over the measurement time with F’O and F’m conditions.

A small comment in addition: I expected to see some actual time resolved spectra the “Ultrafast Fluorescence” sections of the main paper, besides the steady state spectra of PSI and PSII. I understand that these measurements are not new to the community and can be found for example in earlier publications of your group, such as Farooq et al. 2018, but for me it would increase the trust in the time constants found.

Later on, the manuscript contains two modified versions of the commonly used Stern-Volmer equation, for calculation the NPQ value of open and partially open/closed RCs, which were derived in the supporting information, and could be of potential value for others. One thing I’m worried about here is the use of the equation $NPQ^{Open} = k\_NPQ^{Open} / (kf + kd)$ in line 700. What is the reference for it and why is it not $NPQ^{Open} = k\_NPQ^{Open} / (kf + kd +$ kp)? Based on the equation $NPQ^{Closed} = \_NPQ^{Closed} / (kf + kd)$ and comparing it with the equations for Fm and FO it seems wrong not to consider the rate of photochemical quenching kp. As a result, the derived Stern Volmer equation for open RC would be reduced to $NPQ^{Open} =$ (FO-F’O)/F’O, having great impact on the result of the paper.

For the general idea, it makes sense to me, that the “yield” of NPQ decreases, going from closed to open RC, since the open RCs provide an additional energy pathway besides the NPQ dissipation channels, but for me it is not said that also the rate of NPQ (k_NPQ) itself changes. The idea of RC-induced switching of the NPQ rate seems like a good explanation for the results and the underlying mechanisms how this is achieved in nature holds great potential for further research, but I still want to emphasize my above-mentioned concerns on the influence of kp on the NPQ^Open rate.

In summary the manuscript is worked out very well and it gives a great view on the quenching rates of open reaction centers. The consequences in the difference of the open and closed NPQ rate constants are indeed important to consider, for example in the estimated increase in crop yield due to NPQ optimization. Since in nature the reaction centers are always distributed between open and closed, the reduction of the possible yield increase due to NPQ optimization, from 30% to 20% estimated by the authors, may be a bit drastic but the overall message is right.

So, my recommendation is a revision. If the rate constant of the photochemical quenching kp is intentionally left out in the equation of NPQ^Open, I don’t see any problem with publishing this manuscript. The comparison of the absolute intensities of PSI and PSII in open/closed RC under NPQ conditions would have been nice, but since it is not in the main focus of the paper, I understand that it is not explored further. Maybe it should be considered as a topic for a future manuscript.

Reviewer #3 (Comments for the Author):

In this manuscript, the authors characterize a component of the set of photoprotective processes, NPQ, which serve a safety valve under high light in oxygenic photosynthesis. These processes have received a lot of interest over the past several years for the possibility that they can be fine-tuned for biomass gains.

Here, the authors build on their previous work (Ref 30, Nat Plants 2018), where they initially reported an NPQ component specific to the presence of closed PSII RCs, by quantifying this component and discussing its implications for biomass gains based on NPQ. Evaluation of this component is important to understanding NPQ, and so this is a worthwhile study. However, given the complexity of the system, isolating a single component is challenging. While the authors have carefully performed a detailed analysis, there are still aspects of the isolation that need additional clarification prior to publication.

Specifically:
1. In the current analysis, the model seems to assume no crosstalk between the NPQ rate and the photochemistry rate, i.e., the 'lake model.' However, as NPQ increases, the rate of NPQ can interfere with the apparent rate of photochemistry even without an additional NPQ component ascribed to the RC, i.e., the 'puddle model.' Prior to publication, the authors need to demonstrate clearly that it is not this type of cross talk that is giving rise to the observed effect.
2. The authors find a constant PSI lifetime, and ascribe the changes in NPQ to be exclusively due to PSII NPQ. How would PSI-NPQ impact these results?
3. The authors decompose their spectra into a PSI, PSII, and background component (e.g., S17b). However, the background component has a very high amplitude – approximately the same as PSI and about half of PSII. Is the background component consistent across all measurements? Could this be a mis-assigned mixture of PSI and PSII?
4. The extracted parameters, NPQ closed vs NPQ open, are highly processed. It is not clear to me what differences these produce in the actual recorded data (i.e., what percent change in the intensity or lifetime of the fluorescence is due to this effect). This would aid in the evaluation of the likelihood that the extracted parameters would be impacted by some of the factors above.
5. As an editorial comment, the manuscript is extremely technical, making it challenging to read, particularly the actual analysis procedure. Including all the equations used is helpful, but it could be presented more clearly with additional explanation.

Reviewer #4 (Comments for the Author):

Ramakers et al., reported an interesting observation that the open PSII has an instantaneous switch, leading to a quenching rate 35 % lower than that of closed PSII RC, suggesting that plants are more economic than we thought they were. They have essentially solved a long-standing puzzle in the field regarding the quenching rate of open RCs, which has been an experimental challenge for many years, as reduced PQ pool and NPQ are always coupled. And their results are convincing, at the same time, the discussion is provoking.

The approach taken is valid and reasonable; although the data are somewhat noisy, the use of statistics was appropriately applied, which alleviated most of my doubts. The text is well-written, very clear in general, and easy to follow.

A few comments before publishing:
1. It might not be the case that the PSII RC was completely reopen 1 second or 10 seconds after a certain time period of HL treatment. The reduction pathway of the PQ pool in darkness, e.g., electrons from FQR, might still be closing the RCs even without light; would that alter the conclusion to some extent?.
2. Besides the explanation of a faster NPQ rate of open RC provided by the authors, alternatively, could it be possible that the faster rate is simply because of a rapid de-protonation of PsbS or varied interactions between Chls and the quenching molecule/s that relax the quenching on a time-scale of seconds or even milliseconds?

Minor point:
Actinic light should be specified for both figures 1 and 4.

********************END********************

Version 1:

Reviewer comments:

Reviewer #1

(Comments for the Author)
The authors satisfactorily addressed all critical points raised by this reviewer.

However, related to the rather high NPQ determined for wt plants, it was surprising to see in the new Fig. 5 that the PsbS over-

expressing line L17 showed a similar or even somewhat lower maximum NPQ value for closed RC than the wt. This is in contrast to the well-known, up to 50-100 % higher NPQ capacity of L17 (e.g. Li et al. 2002). The authors should provide an explanation for this unexpected result to avoid any confusion of the reader.

(Remarks on code availability)

Reviewer #2

(Comments for the Author)
The authors have now adressed very carefully all my concerns. This is nice work and I now recommend publication as is.

(Remarks on code availability)

Reviewer #3

(Comments for the Author)
The authors have addressed parts of my comments, however major concerns remain. First and foremost, they have not made a compelling case for the need to include an RC-specific component (#4) as they have not shown that it is required to explain the data.

Specifically, they do not adequately address the possibility of crosstalk (#1). In their current analysis, they have to make the assumption that the apparent rates are independent, which is implied in their Stern-Volmer equations. However, in a complex kinetic system, even when the microscopic RC trapping rate and the quencher trapping rate are fixed, the total de-excitation rate when both RC and quencher are active is not necessarily the sum of the de-excitation rates of when only one of RC or quencher is active. Without considering this nonlinear effect, they should not conclude that they are quantifying an quenching component in the RC.

(Remarks on code availability)

Reviewer #4

(Comments for the Author)
I have looked over the authors' response and revisions. The authors have well addressed the comments. I would like to recommend its acceptance.

(Remarks on code availability)

Decision Letter:

3rd February 2026

Dear Professor van Amerongen,

Your study, "Rates of Non-Photochemical Quenching in plants are partially determined by the state of Photosystem II Reaction Centres" has now been seen again by its four Reviewers. You will see from their comments below that they are in general very positive about your study. However, we are concerned by the comments of Reviewers 1 and 3 and would like to see and consider your response to their worries before making a final decision on publication. This can either be a specific response to reviewers letter or if additional data is added, a revised manuscript.
The easiest way to submit these will be through our manuscript tracking system at:

Link Redacted

Note: This URL links to your confidential home page and associated information about manuscripts you may have submitted, or that you are reviewing for us. If you wish to forward this email to co-authors, please delete the link to your homepage.

We hope to receive your response within the next four weeks. If you cannot send it within this time, please let us know.

We look forward to seeing the revised manuscript and thank you for the opportunity to review your work.

Sincerely,

****

Nature Plants is committed to improving transparency in authorship. As part of our efforts in this direction, we are now requesting that all authors identified as 'corresponding author' on published papers create and link their Open Researcher and Contributor Identifier (ORCID) with their account on the Manuscript Tracking System (MTS), prior to acceptance. This applies to primary research papers only. ORCID helps the scientific community achieve unambiguous attribution of all scholarly contributions. You can create and link your ORCID from the home page of the MTS by clicking on 'Modify my Springer Nature account'. For more information please visit please visit www.springernature.com/orcid.

Please do not hesitate to contact me if you have any questions or would like to discuss these revisions further.

****

Reviewers' Comments:

Reviewer #1 (Comments for the Author):

The authors satisfactorily addressed all critical points raised by this reviewer.

However, related to the rather high NPQ determined for wt plants, it was surprising to see in the new Fig. 5 that the PsbS over-expressing line L17 showed a similar or even somewhat lower maximum NPQ value for closed RC than the wt. This is in contrast to the well-known, up to 50-100 % higher NPQ capacity of L17 (e.g. Li et al. 2002). The authors should provide an explanation for this unexpected result to avoid any confusion of the reader.

Reviewer #2 (Comments for the Author):

The authors have now adressed very carefully all my concerns. This is nice work and I now recommend publication as is.

Reviewer #3 (Comments for the Author):

The authors have addressed parts of my comments, however major concerns remain. First and foremost, they have not made a compelling case for the need to include an RC-specific component (#4) as they have not shown that it is required to explain the data.

Specifically, they do not adequately address the possibility of crosstalk (#1). In their current analysis, they have to make the assumption that the apparent rates are independent, which is implied in their Stern-Volmer equations. However, in a complex kinetic system, even when the microscopic RC trapping rate and the quencher trapping rate are fixed, the total de-excitation rate when both RC and quencher are active is not necessarily the sum of the de-excitation rates of when only one of RC or quencher is active. Without considering this nonlinear effect, they should not conclude that they are quantifying an quenching component in the RC.

Reviewer #4 (Comments for the Author):

I have looked over the authors' response and revisions. The authors have well addressed the comments. I would like to recommend its acceptance.

********************END********************

Version 2:

Reviewer comments:

Reviewer #1

(Comments for the Author)
The authors carefully addressed my final concerns

(Remarks on code availability)

Reviewer #3

(Comments for the Author)

The authors still fail to directly address the question that fundamentally challenges their approach: Is the de-excitation rate when both the quencher and RC are active the sum of the de-excitation rates when only quencher is active and when only RC is active? If not, the measured NPQclose and NPQopen will be intrinsically different even without the additional quenching component in the RC. This non-additive effect can usually be expected for complex systems where the kinetics are not exclusively limited by the trapping process. Without proving that this kinetic effect is not contributing to the difference between NPQclose and NPQopen, the claim that an additional quenching component in the RC is not supported.

Another way to view this issue is to look at the Stern-Volmer equations the authors provided. The conclusion of an additional NPQ component requires that the kp and kNPQ in F0’ (both quencher and RC active) are the same as the kp in F0 (only RC active) and the kNPQ in Fm’ (only quencher active). However, these rates are the apparent rates which depend on complex energy transfer and energy trapping processes, and should not be expected to be the same when different numbers of quenchers/RC are present in the system unless proven otherwise. This is what the authors should do in order to justify the additional NPQ component.

In addition, the mutant results are actually consistent with this effect. When there are a lot of NPQ quenchers present in the system (L17), adding more quenchers (i.e., the RC) has a less pronounced effect. On the other hand, when there are fewer quenchers in the system (npq4 and npq1), one could expect the addition of other quenchers (RC) will lead to a larger decrease in lifetime, resulting in a larger difference between NPQclose and NPQopen.

(Remarks on code availability)

Decision Letter:

Our ref: NPLANTS-250720396B

9th June 2026

Dear Dr. van Amerongen,

First let me apologise for the time it has taken me to reach a decision on your study, "Rates of Non-Photochemical Quenching in plants are partially determined by the state of Photosystem II Reaction Centres" (NPLANTS-250720396B). I have attached the Reviewers comments below (which you have already seen) and in the light of these and your informal response to them we have decided that we can in principle to publish the work in Nature Plants, pending minor revisions to comply with our editorial and formatting guidelines.

At the same time I suggest that it would be useful to briefly state your reasons for an explanation in line with Holzwarth and coworkers work from 2002 but that further data will be needed to firmly establish this. Essentially, what you have told me in your response to Reviewer 3. I would suggest using some phrase such as "...although we cannot exclude the possibility that a non-additive kinetic effect is contributing to the difference between NPQclose and NPQopen", but you should address the situation however you think best.

We are now performing detailed checks on your paper and will send you a checklist detailing our editorial and formatting requirements in 1-2 weeks. As the current version of your manuscript is in a PDF format, please email us a copy of the file in an editable format (Microsoft Word or LaTex) to help us with those checks. Please do not upload the final materials or make any revisions until you receive this additional information from us.

Thank you again for your interest in Nature Plants. Please do not hesitate to contact me if you have any questions.

Sincerely,

Reviewer #1 (Comments for the Author):

The authors carefully addressed my final concerns

Reviewer #3 (Comments for the Author):

The authors still fail to directly address the question that fundamentally challenges their approach: Is the de-excitation rate when both the quencher and RC are active the sum of the de-excitation rates when only quencher is active and when only RC is active? If not, the measured NPQclose and NPQopen will be intrinsically different even without the additional quenching component in the RC. This non-additive effect can usually be expected for complex systems where the kinetics are not exclusively limited by the trapping process. Without proving that this kinetic effect is not contributing to the difference between NPQclose and NPQopen, the claim that an additional quenching component in the RC is not supported.

Another way to view this issue is to look at the Stern-Volmer equations the authors provided. The conclusion of an additional

NPQ component requires that the kp and kNPQ in F0’ (both quencher and RC active) are the same as the kp in F0 (only RC active) and the kNPQ in Fm’ (only quencher active). However, these rates are the apparent rates which depend on complex energy transfer and energy trapping processes, and should not be expected to be the same when different numbers of quenchers/RC are present in the system unless proven otherwise. This is what the authors should do in order to justify the additional NPQ component.

In addition, the mutant results are actually consistent with this effect. When there are a lot of NPQ quenchers present in the system (L17), adding more quenchers (i.e., the RC) has a less pronounced effect. On the other hand, when there are fewer quenchers in the system (npq4 and npq1), one could expect the addition of other quenchers (RC) will lead to a larger decrease in lifetime, resulting in a larger difference between NPQclose and NPQopen.

Version 3:

Decision Letter:

3rd July 2026

Dear Professor van Amerongen,

We are pleased to inform you that your Article entitled "The State of Plant Photosystem II Reaction Centres affects the rate of Non-Photochemical Quenching", has now been accepted for publication in Nature Plants.

Over the next few weeks, your paper will be copyedited to ensure that it conforms to Nature Plants style. We look particularly carefully at the titles of all papers to ensure that they are relatively brief and understandable.

Once your paper is typeset, you will receive an email with a link to choose the appropriate publishing options for your paper and our Author Services team will be in touch regarding any additional information that may be required.

After the grant of rights is completed, you will receive a link to your electronic proof via email with a request to make any corrections within 48 hours. If, when you receive your proof, you cannot meet this deadline, please inform us at rjsproduction@springernature.com immediately.

You will not receive your proofs until the publishing agreement has been received through our system.

You may wish to make your media relations office aware of your accepted publication, in case they consider it appropriate to organize some internal or external publicity. Once your paper has been scheduled you will receive an email confirming the publication details. This is normally 3-4 working days in advance of publication. If you need additional notice of the date and time of publication, please let the production team know when you receive the proof of your article to ensure there is sufficient time to coordinate. Further information on our embargo policies can be found here: https://www.nature.com/authors/policies/embargo.html

Due to the importance of these deadlines, we ask you please us know now whether you will be difficult to contact over the next month. If this is the case, we ask you provide us with the contact information (email, phone and fax) of someone who will be able to check the proofs on your behalf, and who will be available to address any last-minute problems.

Acceptance of your manuscript is conditional on all authors' agreement with our publication policies (see http://www.nature.com/authors/policies/index.html). In particular your manuscript must not be published elsewhere.

If you have any questions about our publishing options, costs, Open Access requirements, or our legal forms, please contact ASJournals@springernature.com

Authors may need to take specific actions to achieve compliance with funder and institutional open access mandates. If your research is supported by a funder that requires immediate open access (e.g. according to Plan S principles or the NIH public access policy) then you should select the gold OA route, and we will direct you to the compliant route where possible. Because authors warrant under our subscription licensing terms that they haven’t committed to licensing any version of their article under a licence inconsistent with the terms of our agreement – including the applicable embargo period – publication under the subscription model isn’t suitable for authors whose funders require no embargo.

An online order form for reprints of your paper is available at https://www.nature.com/reprints/author-reprints.html. All co-authors, authors' institutions and authors' funding agencies can order reprints using the form appropriate to their geographical region.

We welcome the submission of potential cover material (including a short caption of around 40 words) related to your

manuscript; suggestions should be sent to Nature Plants as electronic files (the image should be 300 dpi at 210 x 297 mm in either TIFF or JPEG format). Please note that such pictures should be selected more for their aesthetic appeal than for their scientific content, and that colour images work better than black and white or grayscale images. Please include a written description of your image and how it was created along with your image files. Please do not try to design a cover with the Nature Plants logo etc., and please do not submit composites of images related to your work. I am sure you will understand that we cannot make any promise as to whether any of your suggestions might be selected for the cover of the journal.

You can now use a single sign-on for all your accounts, view the status of all your manuscript submissions and reviews, access usage statistics for your published articles and download a record of your refereeing activity for the Nature journals.

To assist our authors in disseminating their research to the broader community, our SharedIt initiative provides you with a unique shareable link that will allow anyone (with or without a subscription) to read the published article. Recipients of the link with a subscription will also be able to download and print the PDF.

As soon as your article is published, you will receive an automated email with your shareable link.

With kind regards,

****

P.S. Click on the following link if you would like to recommend Nature Plants to your librarian
http://www.nature.com/subscriptions/recommend.html#forms

** Visit the Springer Nature Editorial and Publishing website at www.springernature.com/editorial-and-publishing-jobs for more information about our career opportunities. If you have any questions please click here.**

Open Access This Peer Review File is licensed under a Creative Commons Attribution 4.0 International License, which permits use, sharing, adaptation, distribution and reproduction in any medium or format, as long as you give appropriate credit to the original author(s) and the source, provide a link to the Creative Commons license, and indicate if changes were made. In cases where reviewers are anonymous, credit should be given to 'Anonymous Referee' and the source.
The images or other third party material in this Peer Review File are included in the article's Creative Commons license, unless indicated otherwise in a credit line to the material. If material is not included in the article's Creative Commons license and your intended use is not permitted by statutory regulation or exceeds the permitted use, you will need to obtain permission directly from the copyright holder.
To view a copy of this license, visit https://creativecommons.org/licenses/by/4.0/

Dear editor,

Thank you very much for giving us the opportunity to submit a revised version of the manuscript NPLANTS-250720396 "Rates of Non-Photochemical Quenching in plants are partially determined by the state of Photosystem II Reaction Centres" by Ramakers, Lennart; Nui, Yuxi; Harbinson, Jeremy; Bader, Arjen; van Amerongen, Herbert.

We appreciate the effort made by the reviewers and the constructive criticism we received from you and the reviewers. We have used this advice to, we hope, improve the quality of the manuscript. We have addressed all the comments (see below) and have edited the manuscript accordingly. The final revised version and a revised version with all the revisions clearly highlighted/tracked have been uploaded.

We hope that this revised version is now acceptable for publication in Nature Plants.

Best regards on behalf of all the authors,

Lennart Ramakers and Herbert van Amerongen

Answers to the referee comments.

Reviewer #1 (Comments for the Author):

This paper addresses a crucial aspect of the regulation of photosynthetic light utilization. The dissipation of excess light energy as heat (NPQ) is known to serve as important photoprotective mechanism under high light stress, but NPQ is also supposed to limit photosynthetic efficiency under fluctuating light, in particular after high light to low light transitions. The detailed mechanistic understanding of NPQ is thus of high interest for the possible improvement of crop yield by optimizing the NPQ dynamics.

NPQ is typically determined for closed reaction centers (RC) of PSII, and hence in absence of photochemistry, which, however, is not the typical situation in the field. Indeed, earlier work proposed that NPQ is more efficient for closed RCs compared to open PSII RCs. In the present work, the authors addressed this important point and determined the NPQ rates for open and closed PS RCs. Applying both chlorophyll fluorescence lifetime and fluorescence yield measurements, it is clearly shown that the NPQ for open RCs is about 35% lower than for closed RCs. The authors conclude from these data that the NPQ quenching rate is switched in response to the state of the RC, and consequently, that the potential improvement of photosynthetic efficiency by manipulation of the NPQ dynamics is 35% less than originally estimated.

The experimental approach is well-chosen and the paper is written in a very clear and concise way. The conclusions made by the authors are fully supported by the data. However, in my view, it is essential to include mutants that are known to affect specific components contributing to NPQ. In particular, stt7 mutants should be included to clarify unambiguously, to what extent state transitions might contribute to the observed differences between the NPQ rates in open and closed RCs.

Answer: We agree that it is important to consider the effect of state transitions. We already explored these using numerical simulations in the SI of the manuscript in *S2.5* – Effects of illumination driven changes in fluorescence yield. In this section the effects of mechanisms like state transitions on the fluorescence yield correlations are explored and it is concluded that this type of change has no significant effect on the gradient of the correlation. This is important as the $k_{NPQ}^{Open}/k_{NPQ}^{Closed}$ is obtained directly from the gradient. But, for completeness the fluorescence yield correlation between CQ and NPQ^{Closed} for *stn7 A. thaliana* (which lacks state transitions) is also presented here as additional figure 1 and in the manuscript as Fig. S10 in S3.5 (we had already obtained this data but not included them because the MS was already quite long).

The following section has been added to the end of S3.5 in the SI of the manuscript:

'Stn7 A. thaliana lacks state transitions it corresponds to the 'No change' scenario in Fig.S9.(b). The CQ vs NPQ^{Closed} correlation of this mutant is shown in figure S10.

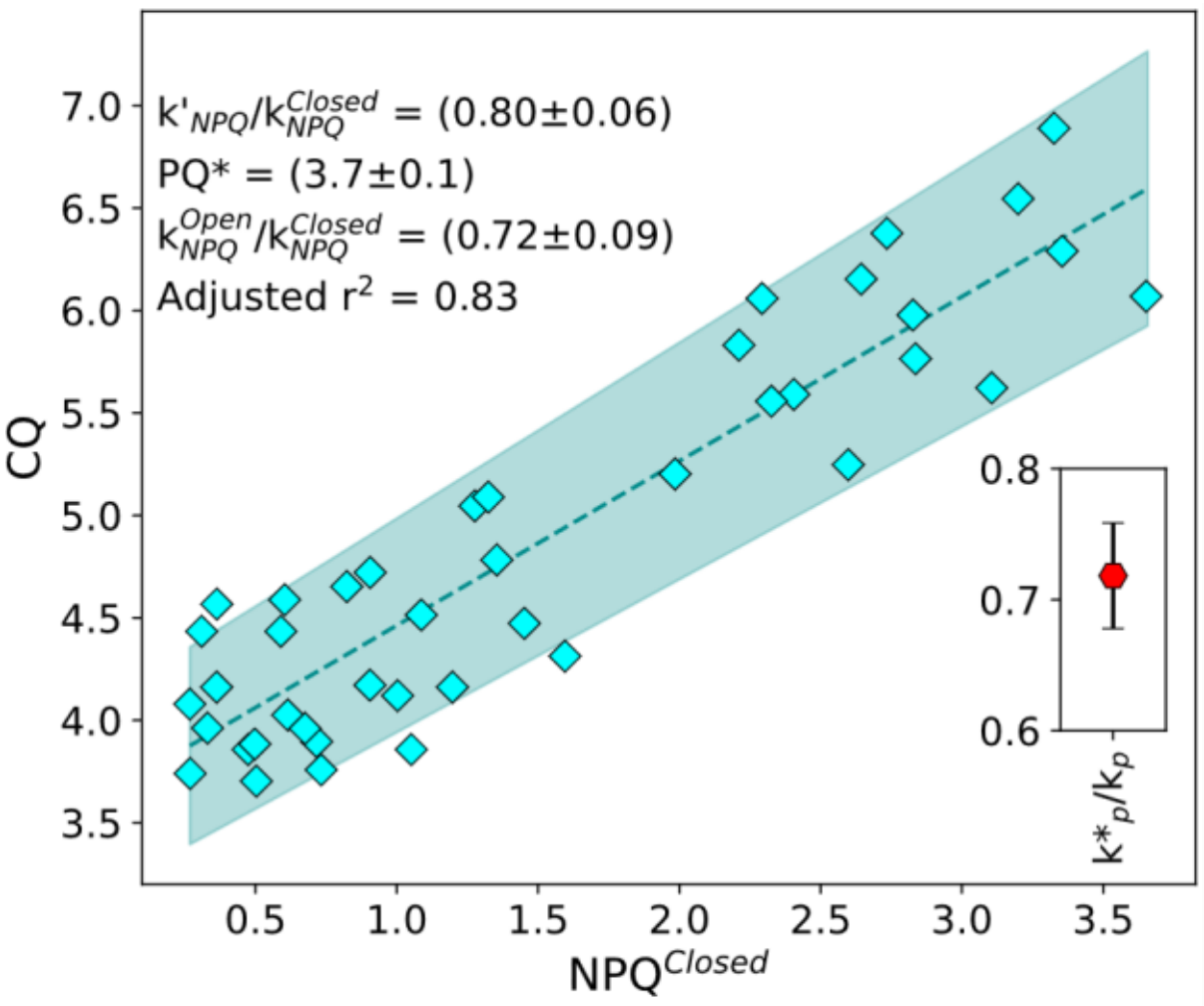

Figure S10: Fitted correlation plot of CQ vs NPQ^{Closed}, with an insert showing the average values of k^*_p/k_p (where $CQ = (F_m - F')/F' = PQ^* + mNPQ^{Closed}$, is the sum of the photochemical and non-photochemical quenching, $NPQ^{Closed} = k^{Closed}_{NPQ}/(k_f + k_d)$ is the value of NPQ when the Q_A pool is fully reduced (fully closed ^{PSII}RCs), k^*_p is the apparent rate of photochemical quenching when the Q_A pool is partially reduced (^{PSII}RCs are partially open), k_p is the rate of photochemical quenching when the Q_A pool is fully oxidised (all ^{PSII}RCs are open) and $PQ^* = k^*_p/(k_f + k_d)$ is the photochemical quenching when the Q_A pool is partially reduced (^{PSII}RCs are partially open)), for *stn7 A. thaliana* (calculated with yields corrected for the contribution of PSI).

Correcting for the fact that not all the ^{PSII}RCs were fully reopened (as outlined in the SI of the original manuscript, S3.9) this correlation reveals that in the absence of state transitions $k^{Open}_{NPQ}/k^{Closed}_{NPQ} = (0.72 \pm 0.09)$. This is noted to be the same (within error) as the wt A. thaliana value (obtained in the presence of state transitions), showing that state transitions do not contribute to the observed discrepancy, in complete agreement with our numerical simulations.'

Moreover, PsbS over-expressing mutants (e.g. L17, Li et al 2002) and the xanthophyll cycle mutant npq1 should be included as well, to determine whether or not PsbS- and/or Zea-dependent NPQ is affected. The experimental effort for such measurements seems to be reasonable, but would provide valuable information about the impact of PsbS and Zea on NPQ in open PSII RCs.

Answer: We agree these mutants yield valuable information but we had left them out because of the length of the manuscript. For a complete exploration we have now also considered the fluorescence yield correlations for *npq4* (PsbS-lacking), *npq1* (Zx-lacking) and *L17* (PsbS-overexpressing) *A. thaliana*.

In *npq1* (Zx-lacking) and *npq4* (PsbS-lacking) plants the mutations lead to a strong decrease in the total amount of NPQ induced during illumination. This means that these plants are more likely to accrue qI during the relatively long light response curve measurements. As such the correlations for these mutants are obtained during the post-illumination recovery period (see subsequent answer exploring the effect of qI on the correlations). These correlations are summarised in Figure 5: (added to the results section)

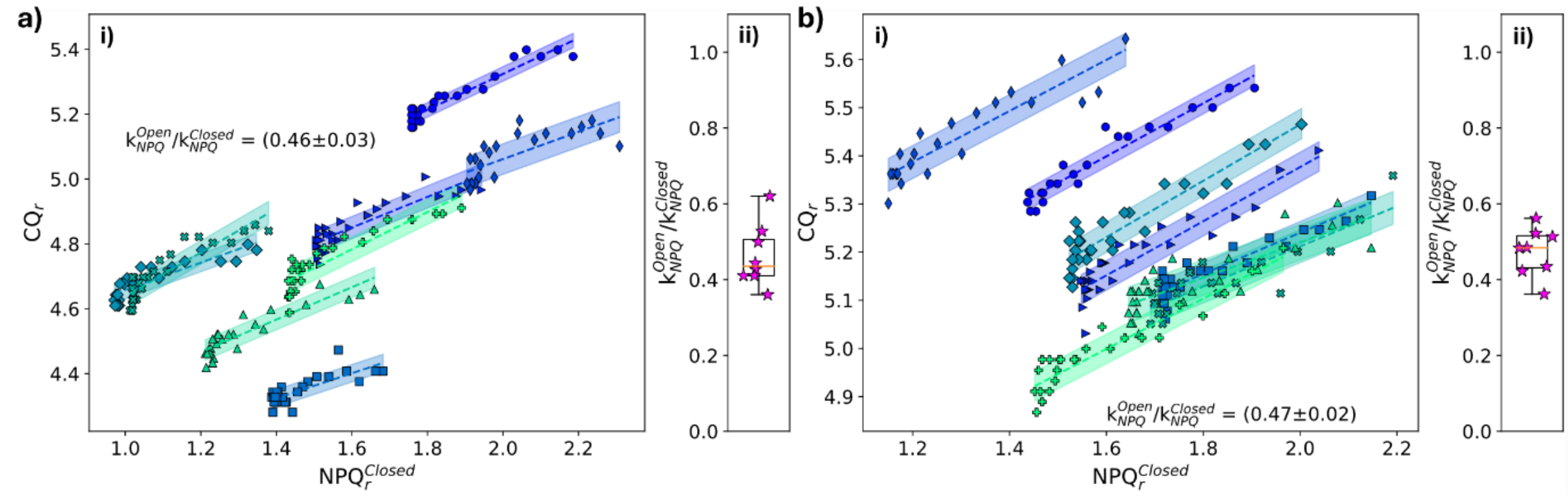

Figure 5: Fitted correlation plot of CQ_r vs NPQ^{Closed} for a) *npq4* and b) *npq1 A. thaliana*. All of the values were calculated at the end of the light response curve during the post-illumination recovery period. All plants were dark adapted overnight prior to measurement and illuminated with red actinic light.

These correlations are consistent with the presence of qI-like processes occurring during the relatively long light response curve measurements, with each individual leaf accruing a different amount of qI during the measurement (see subsequent answer on the effect of qI on the correlations). However, as explained in the subsequent answer during the post-illumination period (duration ~5 mins directly following the final illumination step in the light response curve) the total amount of qI in each leaf remains approximately constant. As such it is possible to obtain $k_{NPQ}^{Open}/k_{NPQ}^{Closed}$ values from these correlations. These values are (0.46 ± 0.03) and (0.47 ± 0.02) for *npq4* and *npq1*, respectively. For *L17*, these correlations are also obtained during the post-illumination recovery period and are summarised in Figure 5.(c): (added to the results section)

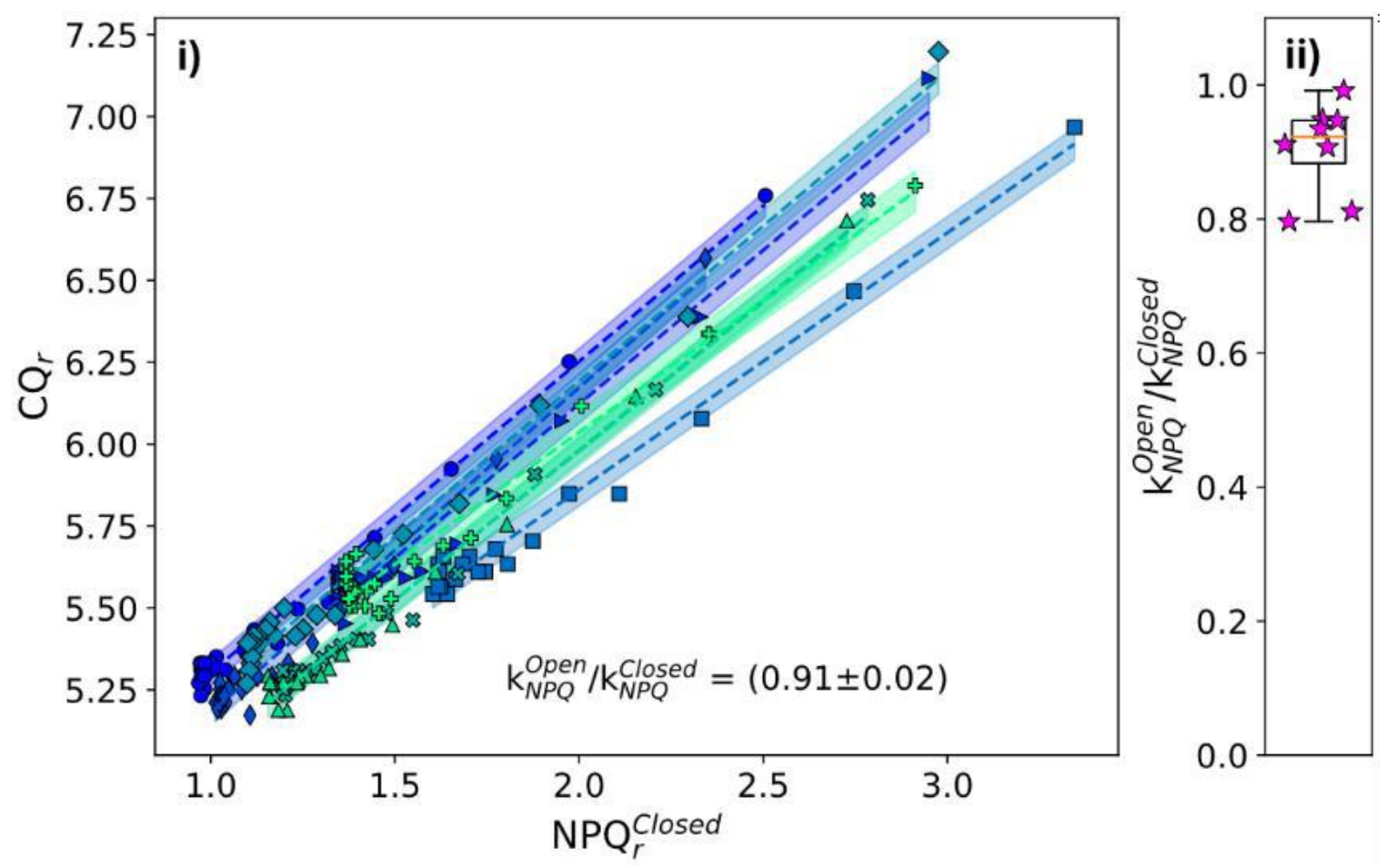

Figure S8: Fitted correlation plot of CQ_r vs NPQ^{Closed} for *L17 A. thaliana*, all of the values were calculated at the end of the light response curve during the post-illumination recovery period. All plants were dark adapted overnight prior to measurement and illuminated with red actinic light.

In *L17* the overexpression of PsbS leads to an increase in the amount of NPQ induced upon illumination. The CQ_r vs NPQ^{Closed} correlations (where the values of CQ and NPQ^{Closed} are obtained during the post-illumination recovery period following the light response curve) reveals $k_{NPQ}^{Open}/k_{NPQ}^{Closed} = (0.91 \pm 0.02)$. Comparing this with the values obtained for *npq4* and *npq1* plants shows that the $k_{NPQ}^{Open}/k_{NPQ}^{Closed}$ ratio increases with increasing amounts of NPQ (qE).

A version of the following text has been added to the end of the discussion:

Overall, the $k^{Open}_{NPQ}/k^{Closed}_{NPQ}$ values for these mutants are consistent with the explanation we outline in our manuscript of additional quenching via the D2 branch β-carotene in closed ^{PSII}RCs, presumed to be unaffected by PsbS expression and Zx levels, and would imply that the ratio should be higher for L17 (with extra PsbS) and this is indeed observed (see Fig 3). This explanation would suggest the correlation gradients are given by $NPQ^{Open}/NPQ^{Closed} = NPQ(Zx+PsbS)/[NPQ(Zx+PsbS)+NPQ(RC)]$. This suggests that as the size of $NPQ(Zx+PsbS)$ increases, the gradient approaches unity. If $NPQ(Zx+PsbS)$ decreases the effect of $NPQ(RC)$ is more pronounced and the gradient decreases away from unity. This is exactly what we observed in these results as $NPQ^{Open}_{npq4} \approx NPQ^{Open}_{npq1} < NPQ^{Open}_{stn7} \approx NPQ^{Open}_{wt} < NPQ^{Open}_{L17}$ and correspondingly $(k^{Open}_{NPQ}/k^{Closed}_{NPQ})_{npq4} \approx (k^{Open}_{NPQ}/k^{Closed}_{NPQ})_{npq1} < (k^{Open}_{NPQ}/k^{Closed}_{NPQ})_{stn7} \approx (k^{Open}_{NPQ}/k^{Closed}_{NPQ})_{wt} < (k^{Open}_{NPQ}/k^{Closed}_{NPQ})_{L17}$.

Other points:

In Figure 4, the maximum NPQ values for closed RCs of about 3.5 are quite high and typically found for Arabidopsis only in PsbS over-expressing lines (e.g. Li et al, 2004). It can thus not be excluded that photoinhibitory quenching (qI) contributes to NPQ at the highest light intensities.

Answer: We agree that these values are higher than typically reported for wt *A. thaliana* but the key difference is that we correct for the constant PSI contribution to the fluorescence yield, and this correction is typically not performed in NPQ measurements. Correcting for the contribution of PSI increases the value of NPQ as this constant correction has a larger effect on F'_m than on F_m (and on F'_o than on F_o). The uncorrected correlations are also reported in the manuscript in the SI, Fig. S6. In this figure the values of NPQ are lower and more in line with those seen for wt *A. thaliana* in the literature. (No further changes made to manuscript).

I wonder, whether this high NPQ is fully reversible upon relaxation of the pH gradient (this could be easily tested at the end of the light response curves). In this context, the authors should also discuss the consequences, if pH-independent NPQ processes, such as qZ or qI, are active under the experimental conditions. The measurements for the light response curves are rather long (70 min in total).

Answer: We agree with the reviewer that these processes are important to consider. Overall, such pH-independent NPQ processes belong to the slowly reversibly NPQ seen in the post-illumination recovery period, meaning that initially the contribution of these processes are approximately constant during the post-illumination NPQ relaxation.

To address these processes, we have performed additional numerical simulations and compared these to the CQ vs NPQ^{Closed} correlations obtained during the post-illumination recovery period. This has now been added to the manuscript in SI, S3.7.

Briefly we show that whilst these processes can affect the correlations, if the correlations are obtained in the initial part of the post-illumination NPQ relaxation then only the intercept of the CQ vs NPQ^{Closed} correlations are altered by these processes while the gradient remains unaffected. Since we derive $k^{Open}_{NPQ}/k^{Closed}_{NPQ}$ from the gradient of the correlation, then for the correlations obtained during the post-illumination recovery qI-like processes don't alter our result. We then also demonstrate, by exploring these recovery CQ vs NPQ^{Closed} correlations (for wt *A. thaliana*) (Fig. S10 in S3.7), that in our measurements the possible small amount of qI thought to be present does not significantly alter the results we present in our manuscript (added to the SI S3.7).

The authors should specify the physiological conditions during those experiments. Were intact leaves attached to the plant used or detached leaves or even leaf discs?

Answer: For all of the fluorescence yield measurements the leaves were secured in the mini-PAM II leaf clip and remained attached to the plant throughout the measurement. For the

fluorescence lifetime measurements the leaves were detached from the plant just before the measurement and transferred to the sample cell. This cell contained a wet paper to provide the leaves with moisture during the measurement. These details have now been added to the manuscript in the methods section, lines 386 and 423 .

Did the setup allow control of the temperature and gas exchange, specifically CO2 availability?

Answer: Neither of the setups presented in the manuscript allowed the temperature or CO_2 availability to be controlled. In the case of temperature both of the experimental setups are located in temperature controlled labs meaning that the ambient temperature is stable throughout the measurement. In the case of CO_2 availability this is currently not possible in the ultrafast spectrometer as the sample cell is required to rotate and so to ensure that the measurements are as equivalent as possible the PAM measurements present in the manuscript were also performed without control over CO_2 availability. However, exploring the correlations obtained during the post-illumination recovery period following illumination, from wt *A. thaliana* measurements performed at 420 ppm [CO_2] yields the following correlations:

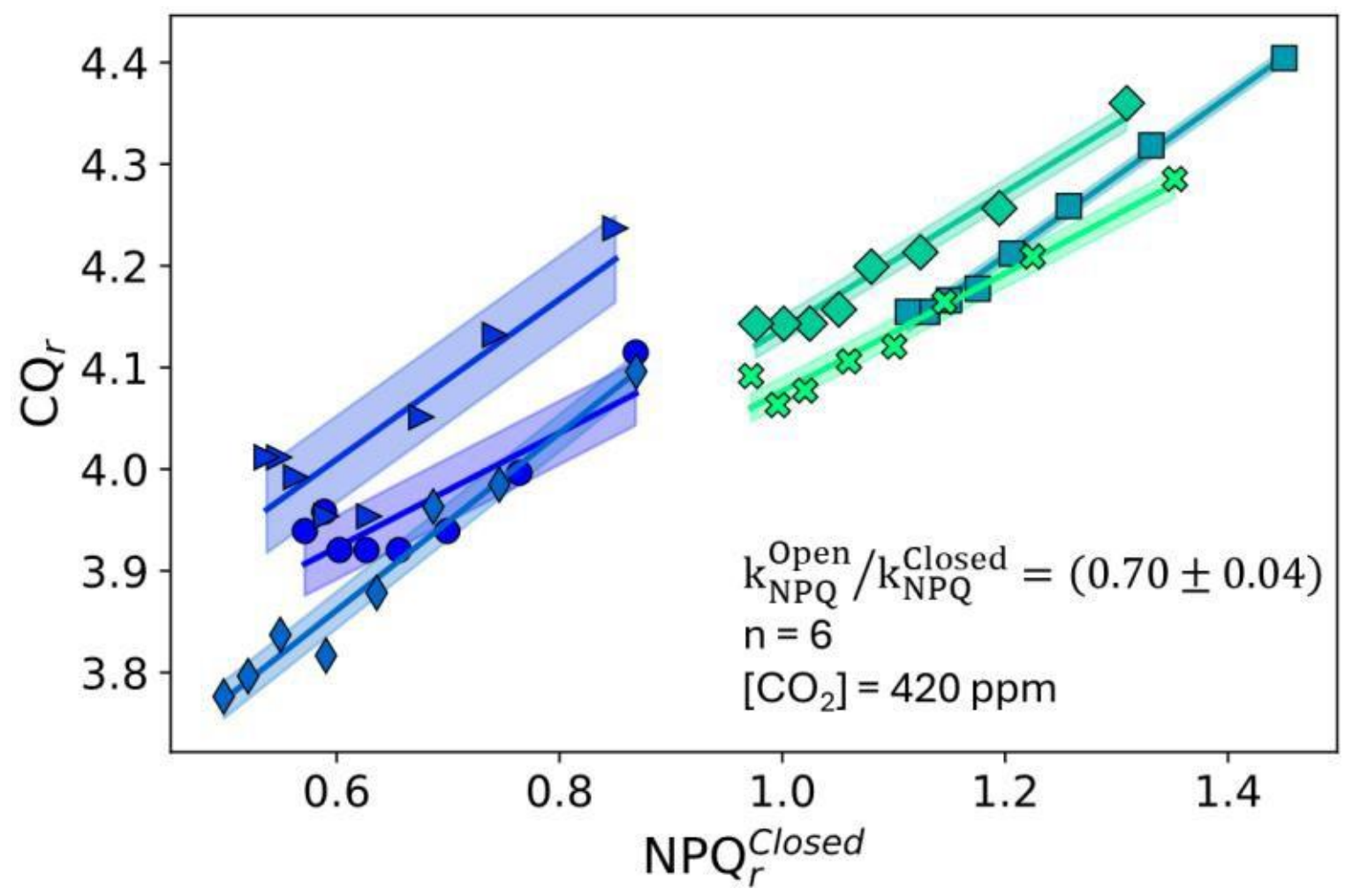

Additional figure 6: (This letter only) Fitted correlation plot of CQ_r vs NPQ_r^{Closed} for wt *A. thaliana* under 420ppm CO_2. All of the values were calculated during the post-illumination recovery period following illumination. All plants were dark adapted overnight prior to measurement and illuminated with red actinic light.

These correlations reveal $k_{NPQ}^{Open}/k_{NPQ}^{Closed} = (0.70 \pm 0.04)$, when the [$CO_2$] is controlled and set to 420 ppm (current atmospheric levels). This value is within error of the value obtained from the PAM measurements present in the manuscript illustrating that slight fluctuations in [CO_2] (possibly present in our measurements) are unlikely to significantly alter our result. So as not to dilute the main massage of our manuscript this dataset is only presented here.

We thank the reviewer for their constructive and positive evaluation.

Reviewer #2 (Comments for the Author):

The provided manuscript, entitled “Rates of Non-Photochemical Quenching in plants are partially determined by the state of Photosystem II Reaction Centres”, addresses the question on how the quenching rates of non-photochemical quenching (NPQ) are influenced by the photochemical quenching (PQ) of the open reaction center. Since in nature, most of the time both photochemical and non-photochemical quenching are present simultaneously, it is of interest to explore quenching rates in the fully open and intermediate RC conditions, besides the well documented closed condition. To gain insights, experiments were performed on a self-made ultrafast fluorescence lifetime setup and on a

commercial PAM fluorometer to collect time-resolved spectra and steady state fluorescence intensities respectively. Experiments were performed on dark and light adapted leaves of wt A. Thaliana.

In the results section, the fluorescence emission spectra of PSI and PSII of the fully closed and fully open RCs, obtained from applying ICA to the ultrafast fluorescence, are in good agreement to others. Also, the lifetimes of PSI and PSII, the decrease in PSII lifetime upon illumination, the absence of PSI NPQ adaptation and the overall NPQ fits well into literature. One opportunity that I think is missed out here, is a comparison of the intensities of PSI and PSII relative to each other, since all spectra are normalised to 1. I have a paper in mind where this was achieved for dark adapted plants by using excitation and detection conditions specific for PSI (F. Franck et al. / Biochimica et Biophysica Acta 1556 (2002) 239–246), instead of a data analysis approach. It would be interesting to see how much the PSI and PSII fluorescence spectra account to the total F'O and F'm steady state spectra. I imagine something similar to the spectrum in figure S14 c), but not for a temporal slice but for the full integration over the measurement time with F'O and F'm conditions.

Answer: We agree with the reviewer that it would be interesting but we also agree with the reviewers statement below "*The comparison of the absolute intensities of PSI and PSII in open/closed RC under NPQ conditions would have been nice, but since it is not in the main focus of the paper, I understand that it is not explored further. Maybe it should be considered as a topic for a future manuscript.*" Apart from the main focus argument we are also worried about the length of manuscript and a potential "dilution effect". (No further changes made to manuscript).

A small comment in addition: I expected to see some actual time resolved spectra the "Ultrafast Fluorescence" sections of the main paper, besides the steady state spectra of PSI and PSII. I understand that these measurements are not new to the community and can be found for example in earlier publications of your group, such as Farooq et al. 2018, but for me it would increase the trust in the time constants found.

Answer: We are of course happy to include these spectra here and in the SI of the manuscript.

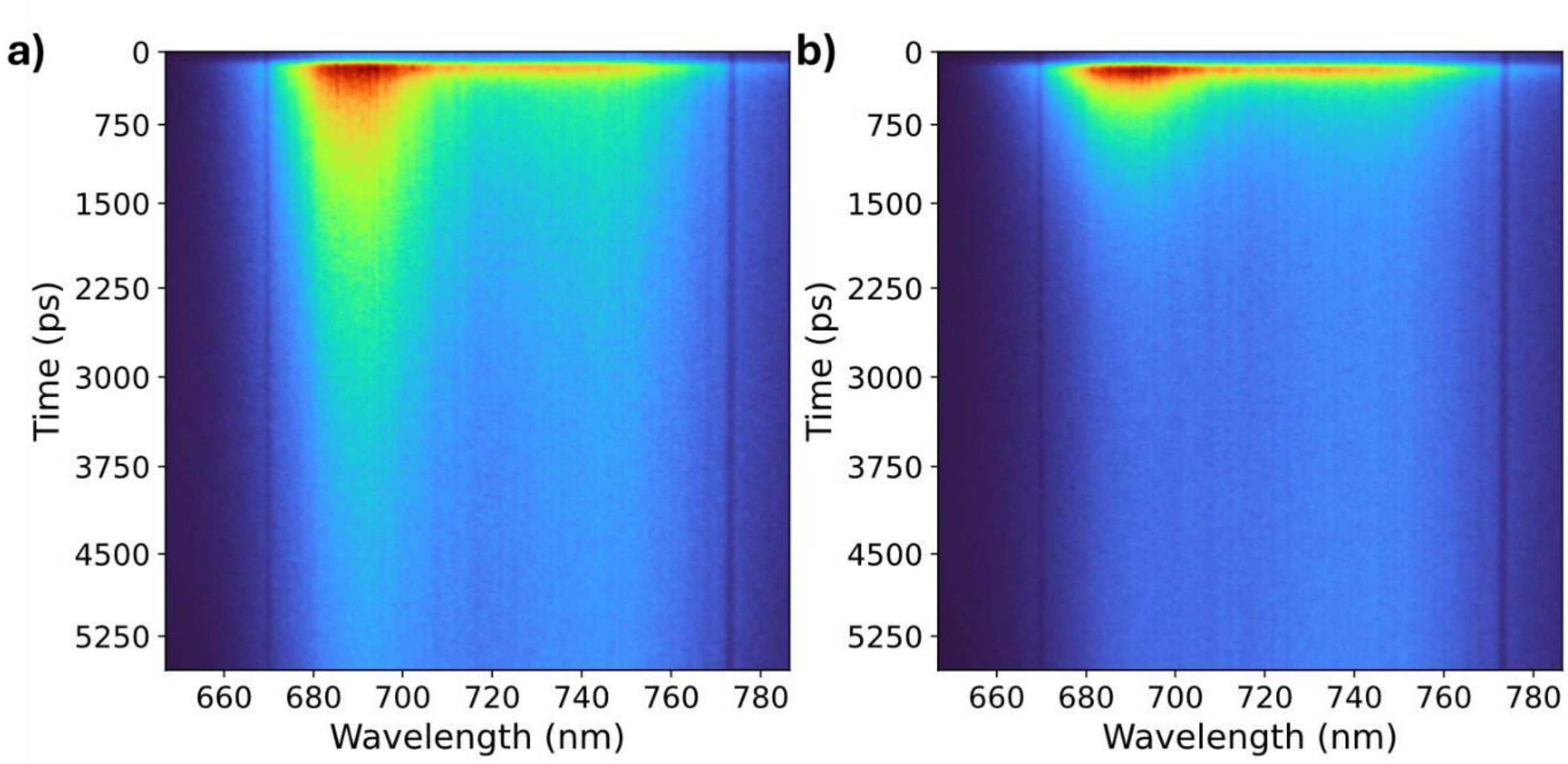

Figure S5: Representative ultrafast fluorescence spectra of wt *A. thaliana* under a) F_m and b) F'_m conditions.

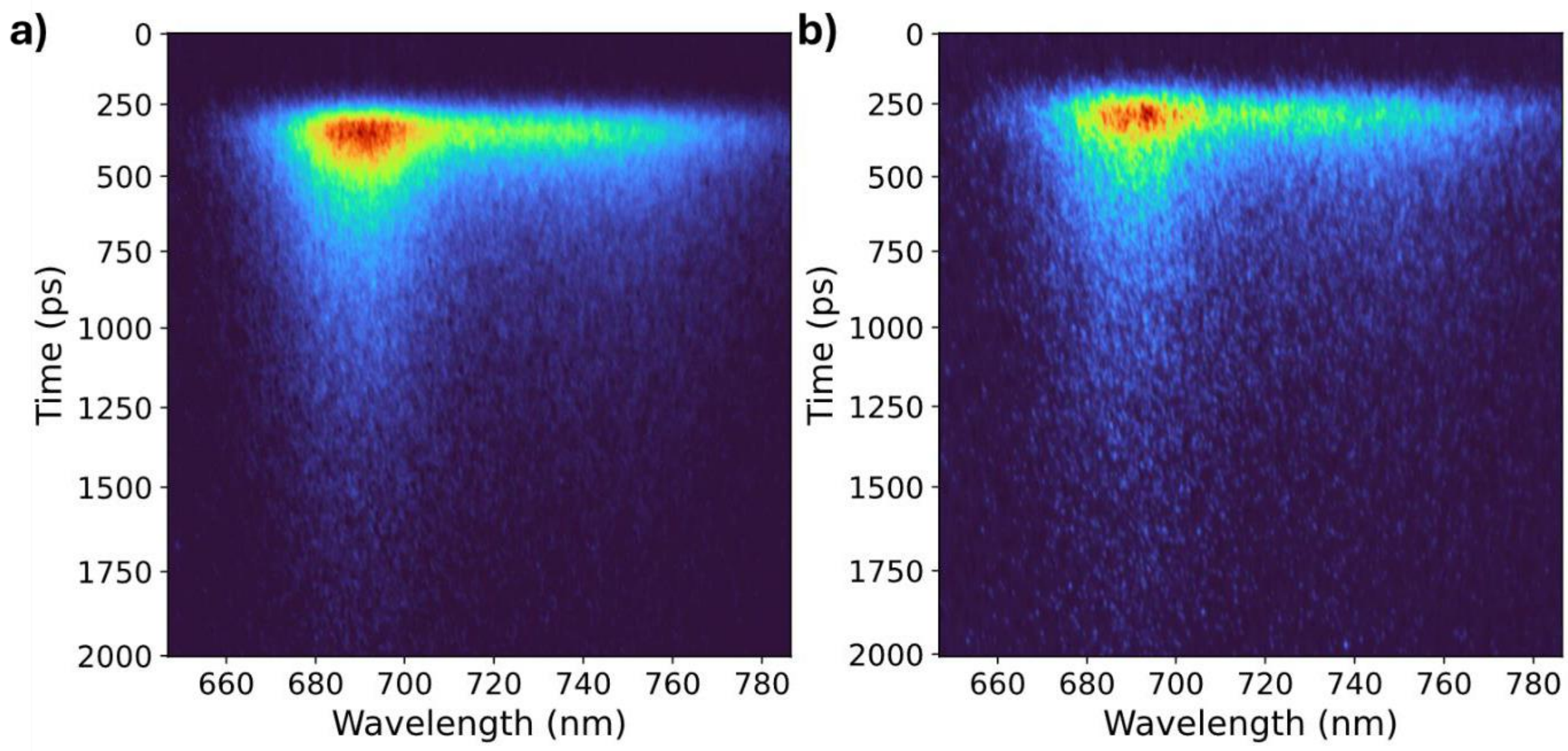

Figure S6: Representative ultrafast fluorescence spectra of wt *A. thaliana* under a) F_o and b) F'_o conditions.

Later on, the manuscript contains two modified versions of the commonly used Stern-Volmer equation, for calculation the NPQ value of open and partially open/closed RCs, which were derived in the supporting information, and could be of potential value for others. One thing I'm worried about here is the use of the equation $NPQ^\wedge Open = k\_NPQ^\wedge Open / (kf + kd)$ in line 700. What is the reference for it and why is it not $NPQ^\wedge Open = k\_NPQ^\wedge Open / (kf + kd + kp)$? Based on the equation $NPQ^\wedge Closed = k\_NPQ^\wedge Closed / (kf + kd)$ and comparing it with the equations for Fm and FO it seems wrong not to consider the rate of photochemical quenching kp. As a result, the derived Stern Volmer equation for open RC would be reduced to $NPQ^\wedge Open$ = (FO-F'O)/F'O, having great impact on the result of the paper.

Answer: There is no reference for the equation $NPQ^\wedge Open = k\_NPQ^\wedge Open / (kf + kd)$ as this equation is derived from the fluorescence yield equations in this manuscript. As such we included this derivation in the SI of this manuscript (see section S2.2).

Here, we specifically chose not to include the rate of photochemistry in $NPQ^\wedge Open$ as we want to compare it directly with the original Stern-Volmer equation ($NPQ^\wedge Closed = k\_NPQ^\wedge Closed / (kf + kd)$) to understand if the presence of open ^{PSII}RCs changes k_{NPQ}. For closed RCs we determine NPQ = $(F_m - F_m')/F_m'$ and this reflects NPQ = $k_{NPQ}/(k_f + k_d)$. In fact, we are most interested in k_{NPQ} and to compare that for open and closed RCs. Now we can obtain a similar parameter for open RCs and measure $(F_0 - F_0')/ F_0'$ but this also contains indeed the rate of photosynthesis, and we don't want that. We want to compare k_{NPQ}. Therefore, we multiply $(F_0 - F_0')/F_0'$ with F_m/F_o and this product is equal to $k_{NPQ}/(k_f + k_d)$ but now k_{NPQ} is the rate for open RCs. For the rest this is identical to NPQ(closed) = $k_{NPQ}/(k_f + k_d)$ with k_{NPQ} being the rate for closed RCs and therefore we call this NPQ(open).

We could also have directly compared the rate of NPQ for open and closed RCs, as was done before in (Farooq et al, 2018) and which leads to identical ratios but NPQ is a more commonly used parameter and can also be obtained directly (and independently) from the steady state PAM measurement, that is why we switched.

We think that relating these rates is a good way to relate our manuscript to the earlier work of Farooq *et al*. we have now added this to the SI, Notes S2. This note contains the following information:

Comparing the rates of NPQ for open and closed RCs (as done in Farooq et al, 2018) we obtain the following result:

$$k_{NPQ}^{Closed} = \frac{1}{\langle \tau_{F_m'}^{PSII} \rangle} - \frac{1}{\langle \tau_{F_m}^{PSII} \rangle} = (1.2 \pm 0.1)\ ns^{-1}$$

(Obtained using the experimental values from the Ultrafast Fluorescence with fully closed ^{PSII}RCs (F_m and F_m') in the results section of the current manuscript)

$$k_{NPQ}^{Open} = \frac{1}{\langle \tau_{F_o'}^{PSII} \rangle} - \frac{1}{\langle \tau_{F_o}^{PSII} \rangle} = (0.7 \pm 0.1)\ ns^{-1}$$

(Obtained using the experimental values from the Ultrafast Fluorescence with fully open ^{PSII}RCs (F_o and F_o') in the results section)

Using these values, we find that $k_{NPQ}^{Open}/k_{NPQ}^{Closed} = (0.6 \pm 0.1)$, fully consistent (within error) with the results of our fluorescence yield correlations.

For the general idea, it makes sense to me, that the "yield" of NPQ decreases, going from closed to open RC, since the open RCs provide an additional energy pathway besides the NPQ dissipation channels, but for me it is not said that also the rate of NPQ (k_NPQ) itself changes. The idea of RC-induced switching of the NPQ rate seems like a good explanation for the results and the underlying mechanisms how this is achieved in nature holds great potential for further research, but I still want to emphasize my above-mentioned concerns on the influence of kp on the NPQ^Open rate.

Answer: the whole point of the current manuscript is to demonstrate that not only the yield of NPQ decreases upon opening the RCs but also the rate of NPQ. (No further changes made to manuscript).

In summary the manuscript is worked out very well and it gives a great view on the quenching rates of open reaction centers. The consequences in the difference of the open and closed NPQ rate constants are indeed important to consider, for example in the estimated increase in crop yield due to NPQ optimization. Since in nature the reaction centers are always distributed between open and closed, the reduction of the possible yield increase due to NPQ optimization, from 30% to 20% estimated by the authors, may be a bit drastic but the overall message is right.

Answer: we are glad that the reviewer agrees with the overall message although we do not believe that the estimated reduction from 30% to 20% is too drastic based on our findings. (No further changes made to manuscript).

So, my recommendation is a revision. If the rate constant of the photochemical quenching kp is intentionally left out in the equation of NPQ^Open, I don't see any problem with publishing this manuscript. The comparison of the absolute intensities of PSI and PSII in open/closed RC under NPQ conditions would have been nice, but since it is not in the main focus of the paper, I understand that it is not explored further. Maybe it should be considered as a topic for a future manuscript.

Answer: we thank the reviewer for their constructive and positive evaluation.

Reviewer #3 (Comments for the Author):

In this manuscript, the authors characterize a component of the set of photoprotective processes, NPQ, which serve a safety valve under high light in oxygenic photosynthesis. These processes have received a lot of interest over the past several years for the possibility that they can be fine-tuned for biomass gains.

Here, the authors build on their previous work (Ref 30, Nat Plants 2018), where they initially reported an NPQ component specific to the presence of closed PSII RCs, by quantifying this component and

discussing its implications for biomass gains based on NPQ. Evaluation of this component is important to understanding NPQ, and so this is a worthwhile study. However, given the complexity of the system, isolating a single component is challenging. While the authors have carefully performed a detailed analysis, there are still aspects of the isolation that need additional clarification prior to publication.

Specifically:

1. In the current analysis, the model seems to assume no crosstalk between the NPQ rate and the photochemistry rate, i.e., the 'lake model.' However, as NPQ increases, the rate of NPQ can interfere with the apparent rate of photochemistry even without an additional NPQ component ascribed to the RC, i.e., the 'puddle model.' Prior to publication, the authors need to demonstrate clearly that it is not this type of cross talk that is giving rise to the observed effect.

Answer: We are of course aware of the lake model and the puddle model although we do not exactly understand what the reviewer means with cross-talk. Nevertheless, it is important to emphasize here that we did actually determine the ratio of NPQ for open and closed RCs over a large range of light intensities and NPQ values and the ratio is constant within error. Furthermore, we note that as our NPQ values are either measured for either fully open or closed ^{PSII}RCs and all CQ values are obtained when as many ^{PSII}RCs are open as possible (see Fig.S2.(b)) we note that we don't assume either the lake or puddle model (as this is not required for our derivations and under these conditions qP and qL should agree). (No further changes made to the manuscript).

2. The authors find a constant PSI lifetime, and ascribe the changes in NPQ to be exclusively due to PSII NPQ. How would PSI-NPQ impact these results?

Answer: we are not fully sure how the reviewer would define PSI-NPQ. We see two possibilities:

1- **After charge separation in PSI, P700 becomes P700+ and this is known to be a very good quencher of excitations and therefore fluorescence and one might indeed call that NPQ. However, this does not change the fluorescence lifetime/yield of PSI (see also recent review in The Plant Cell on NPQ; https://doi.org/10.1093/plcell/koaf240).**

2- **There is some extra inducible NPQ that leads to extra fluorescence quenching. However, as was also discussed in (https://doi.org/10.1093/plcell/koaf240) this does not happen and both lifetime and yield remain the same (change is <1%) during NPQ conditions. We can add to this that the results shown in fig.1 in our manuscript show that over 20 minutes of illumination at high light intensity the lifetime of PSI does not significantly change (the error margin on this is ~5% based on our ultrafast measurements) which indicates that we do not see any significant quenching of PSI-NPQ in contrast to the large quenching of PSII with the concomitant change in lifetime and the observed NPQ can be entirely ascribed to PSII (No further changes made to manuscript).**

3. The authors decompose their spectra into a PSI, PSII, and background component (e.g., S17b). However, the background component has a very high amplitude – approximately the same as PSI and about half of PSII. Is the background component consistent across all measurements? Could this be a mis-assigned mixture of PSI and PSII?

Answer: The background is the steady state fluorescence induced by the illumination of the leaves with the CW laser needed to fully close the ^{PSII}RCs and the actinic light source needed to induce NPQ (Since it is chlorophyll fluorescence of course spectrally it will look like a mixture of the PSI and PSII spectra).

As such there is no steady state background in either the F_o or F'_o, as these are measured in the dark and with fully open ^{PSII}RCs.

Both the F_m and F'_m measurements have steady state backgrounds, in these two conditions the spectral form of this steady state background is not the same due to the fact that in the F'_m condition the amplitude of steady state fluorescence yield of PSII is lower due to NPQ.

However, we know that this is a steady state fluorescence background as it is always present (even before time zero, when the ultrafast laser pulse arrives at the sample) and its overall contribution to the fluorescence is constant over time (throughout the ~5.5 ns measurement window used in these measurements).

Finally, it is repeatably the same over the triplicate F_m and F'_m measurements. Together, these facts assure us that this is a steady state background due to the continuous illumination of the CW laser and/or the actinic light source. This has now been added to the SI, as S4.2:

S4.2 – Steady state fluorescence backgrounds (F_m and F'_mspectra)

The F_m and F'_m spectra are measured in the presence of a steady state light source. For the F_m spectra this steady state light source is the CW laser used to ensure that all the ^{PSII}RCs are closed whereas for the F'_m spectra this steady state light source is the combination of the CW laser and the actinic light used to induce NPQ. These steady state fluorescence backgrounds are summarised in figure S20:

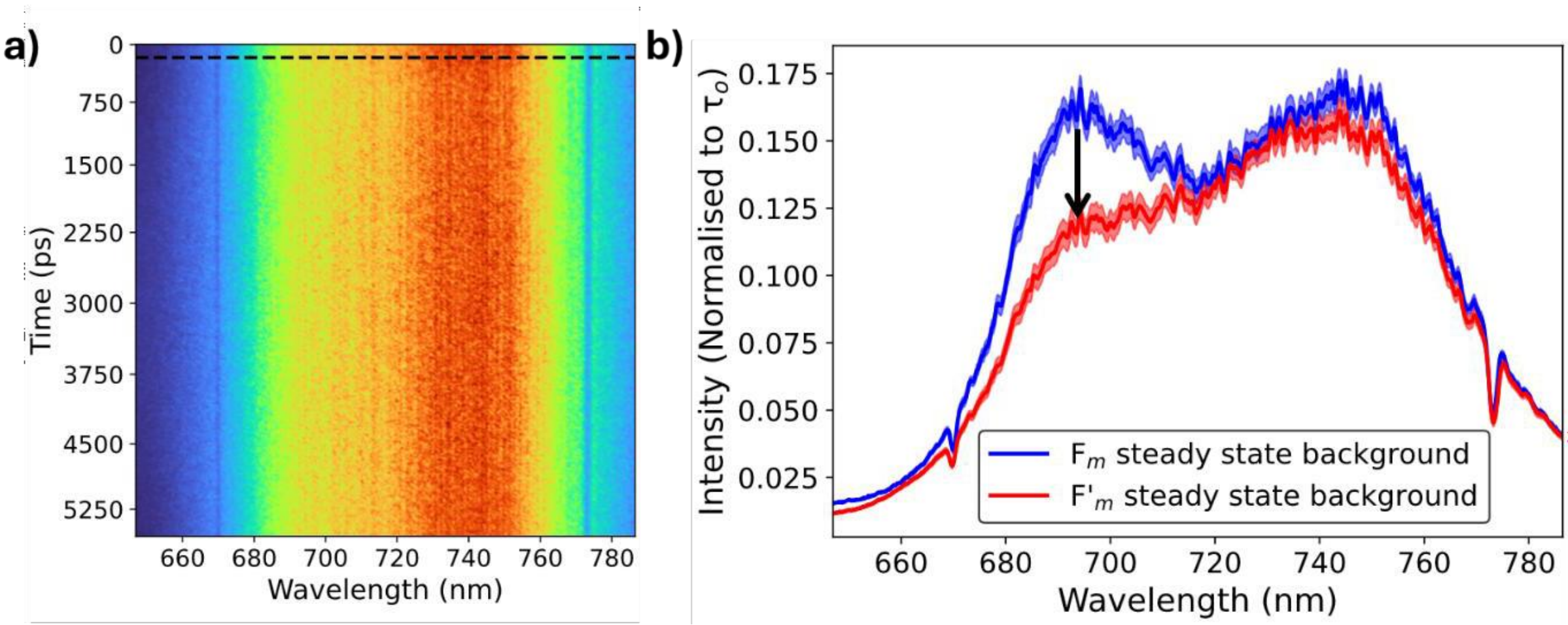

***Figure S20*: Representative steady state background fluorescence spectra of wt *A. thaliana* under a) F'_m conditions (without ultrafast laser, τ_o indicated by dashed black line) and b) the average (n = 3) F_m (blue) and F'_m (red) steady state background fluorescence spectra of wt *A. thaliana* (the effect of NPQ is indicated by a black arrow). Shaded area shows the associated standard error.**

A representative steady state background fluorescence spectrum measured following NPQ induction, without the ultrafast laser pulses, is shown for wt *A. thaliana* in the F'_m state is shown in figure S20.(a). This spectrum reveals that this fluorescence signal is constant over time and always present, even before τ_o (the arrival time of the ultrafast laser pulse when this is not blocked). This reveals that this steady state background is not part of the ultrafast fluorescence signal induced by the ultrafast laser pulse. Furthermore, the steady state background spectra are very consistent across all the replicas (n = 3) for both the F_m and F'_m

measurements (Fig.S20.(b)) and appear to be mixtures of the steady state PSII and PSI spectra suggesting that these are due to the illumination of the leaves with constant light sources. This is also supported by the spectral differences seen between the steady state backgrounds seen for F_m and F_m' which are consistent with the quenching of the PSII dominated part of the spectrum due to the presence of NPQ (black arrow, Fig.S20.(b)).

4. The extracted parameters, NPQ closed vs NPQ open, are highly processed. It is not clear to me what differences these produce in the actual recorded data (i.e., what percent change in the intensity or lifetime of the fluorescence is due to this effect). This would aid in the evaluation of the likelihood that the extracted parameters would be impacted by some of the factors above.

Answer: The deconvolution of ultrafast spectra into their underlying PSII and PSI spectra is widely done for these types of measurements. Though it is normally done using global analysis, as reviewer 2 in fact points out '*… the fluorescence emission spectra of PSI and PSII of the fully closed and fully open RCs, obtained from applying ICA to the ultrafast fluorescence, are in good agreement to others.***'. We agree with reviewer 2 on this point and think this supports how sound our analysis is as it finds answers which converge with other mathematical deconvolution approaches. Furthermore, the PAM data also agrees with the ultrafast measurements. The difference in the fluorescence due to NPQ can directly be seen in the fluorescence yield trace. Specifically, we see this when we compare the measured values with those calculated in the case where k_{NPQ} is unaffected by the state of the RCs. For this we derive the following novel expression to calculate F'_o:**

$$NPQ^{Open} = mNPQ^{Closed}, \text{where } m \in \mathbb{R}_+ \ (Fig. 4. (a))$$

$$\Rightarrow \frac{F_m}{F_o}\left(\frac{F_o - F_o'}{F_o'}\right) = mNPQ^{Closed}$$

Which can be rearranged to yield $F'_{o,\ calc}$:

$$\Rightarrow F'_{o,\ calc} = \frac{F_m F_o}{(mF_o NPQ^{Closed} + F_m)}, \text{where } m \in \mathbb{R}_+$$

In the case where k_{NPQ} is unaffected by the state of the RCs, m = 1 and this equation simplifies to:

$$F'_{o,\ calc} = \frac{F_m F_o}{(F_o NPQ^{Closed} + F_m)} \ (\text{using } m = 1)$$

Allowing us to calculate F'_o using just the value of Fm and Fo as well as the NPQ (obtained from the Stern-Volmer equation).Here we have now included an additional figure to show the difference between the average measured F'_o values (n=8) and the values of F'_o that would be expected if there was no 'instantaneous' RC-induced NPQ switching (calculated using the equation above, additional figure 7):

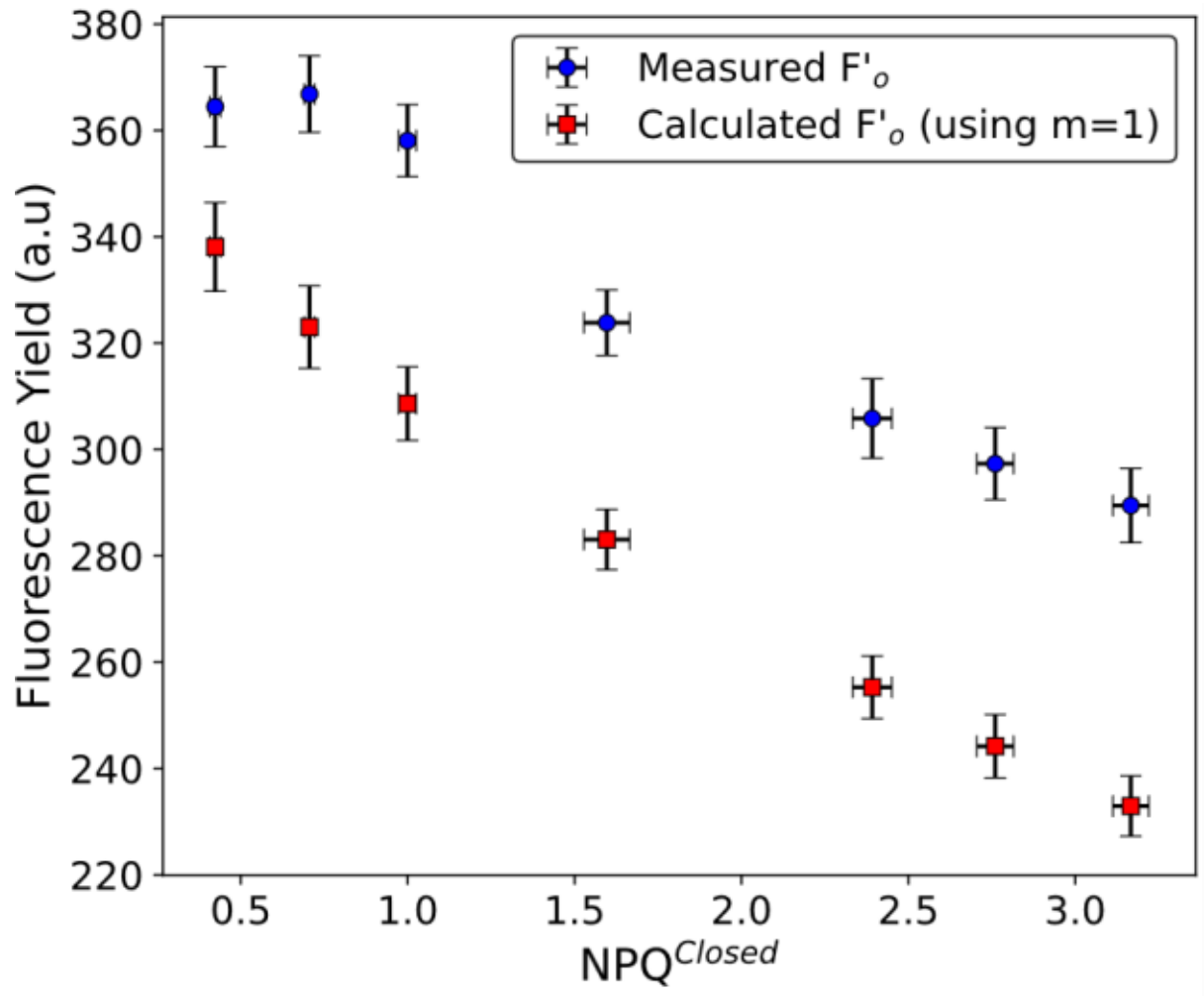

Additional figure 7: Comparison between the average measured F_o' values (n = 8, blue circles) and average calculated F_o' (n = 8, calculated using $m = 1$ ($\Rightarrow k_{NPQ}^{Open}/k_{NPQ}^{Closed} = 1$); red squares) values for wt *A. thaliana*. All measured F'_o values are corrected for the contribution of PSI to the fluorescence yield. All plants were dark adapted overnight prior to measurement and illuminated with red actinic light.

This comparison shows sizeable absolute differences in the fluorescence yield, with the measured values being larger than the calculated values (based on k_{NPQ}^{Closed}). This difference is completely consistent with the results shown in the manuscript.

Furthermore, if we use the PSII lifetimes we find that the ratio between k_{NPQ}^{Open} and k_{NPQ}^{Closed} is completely consistent (within error) with the results we found using the fluorescence yield correlation (see answers to reviewer 2 above), for completeness this has now also been added to the SI of the manuscript.

5. As an editorial comment, the manuscript is extremely technical, making it challenging to read, particularly the actual analysis procedure. Including all the equations used is helpful, but it could be presented more clearly with additional explanation.

Answer: We understand that the current manuscript is quite technical, which can make it somewhat challenging to read. The derivation of the equations are already given in as much detail as possible in the SI. This is also true of the analysis procedure for both the PAM and ultrafast fluorescence measurements as we appreciate the complexity of these aspects. We would not really know how to make the text more accessible without significantly increasing the word count. (No further changes made to manuscript).

We thank the reviewer for their constructive and positive evaluation.

Reviewer #4 (Comments for the Author):

Ramakers et al., reported an interesting observation that the open PSII has an instantaneous switch, leading to a quenching rate 35 % lower than that of closed PSII RC, suggesting that plants are more economic than we thought they were. They have essentially solved a long-standing puzzle in the field regarding the quenching rate of open RCs, which has been an experimental challenge for many years, as reduced PQ pool and NPQ are always coupled. And their results are convincing, at the same time, the discussion is provoking.

The approach taken is valid and reasonable; although the data are somewhat noisy, the use of statistics was appropriately applied, which alleviated most of my doubts. The text is well-written, very clear in general, and easy to follow.

A few comments before publishing:

1. It might not be the case that the PSII RC was completely reopen 1 second or 10 seconds after a certain time period of HL treatment. The reduction pathway of the PQ pool in darkness, e.g., electrons from FQR, might still be closing the RCs even without light; would that alter the conclusion to some extent?.

Answer: We agree that this is possible and that the reduction pathways of the PQ pool in darkness could be important, however we do not think that this has a particularly strong impact on our results here. The reason for this is the close agreement in the $k_{NPQ}^{Open}/k_{NPQ}^{Closed}$ values obtained from the CQ and NPQ^{Closed} correlations calculated from the data obtained during the light response curve and during the post-illumination period following the light response curve ((0.64 ± 0.02) and (0.70 ± 0.05), respectively).

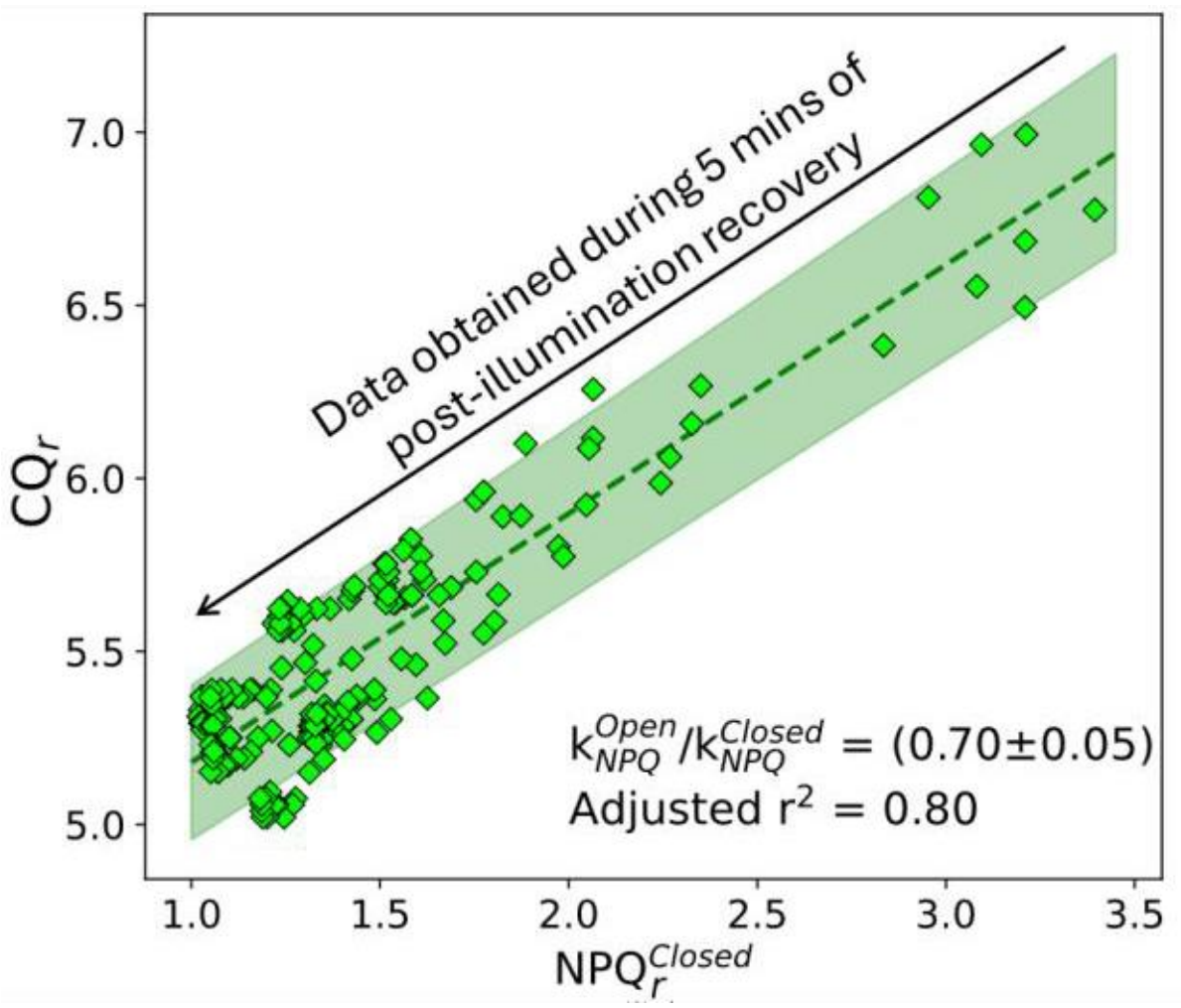

Additional figure 8: Fitted correlation plot of CQ_r vs NPQ^{Closed} (values obtained during the 5 minutes of post-illumination recovery following the light response curve) for wt *A. thaliana*. All plants were dark adapted overnight prior to measurement and illuminated with red actinic light.

These CQ and NPQ^{Closed} correlations are obtained during the ~5 minutes of post-illumination following the light response curve. Throughout this time the redox state of the PQ pool is presumably undergoing measurable changes *via* reduction pathways. However, the correlation is still linear and still yields the same $k_{NPQ}^{Open}/k_{NPQ}^{Closed}$ value (within error). This indicates that these changes in the redox state of the PQ pool have no significant effect(s) on our result. (No further changes made to manuscript).

2. Besides the explanation of a faster NPQ rate of open RC provided by the authors, alternatively, could it be possible that the faster rate is simply because of a rapid de-protonation of PsbS or varied interactions between Chls and the quenching molecule/s that relax the quenching on a time-scale of seconds or even milliseconds?

Answer: We agree that there are other possible explanations. The most important point for us is that we verified with three independent methods that there is a clear difference between the (rate of) NPQ in the case of open and closed RCs and the rate of switching between the two rates are nearly instantaneous. The findings in the earlier manuscript of Holzwarth and coworkers provide a very nice explanation for these finding and it also seems to fit quantitatively. It also explains the finding that the ratio of the rates increases when PsbS and

Zx are absent and decreases when the amount of PsbS increases. This was now added to the manuscript (see answers to reviewer 1) These results also exclude that the faster rate is simply due to a rapid de-protonation of PsbS as this rapid change in NPQ quenching rate is also seen in *npq4 A. thaliana* (Additional fig. 9), where there is no PsbS and the ratio of NPQ values is becoming even bigger.

Regarding the suggested option that the effect may be due to varied interactions between Chls and the quenching molecule is not likely in our opinion. The NPQ that occurs in the antenna complexes is indeed due to the interaction between Chls and quenchers (supposedly carotenoids) but we fail to see how this interaction would respond to the opening/closing of RCs on a time scale of seconds or even milliseconds. Therefore, we prefer not to provide this as a plausible alternative explanation.

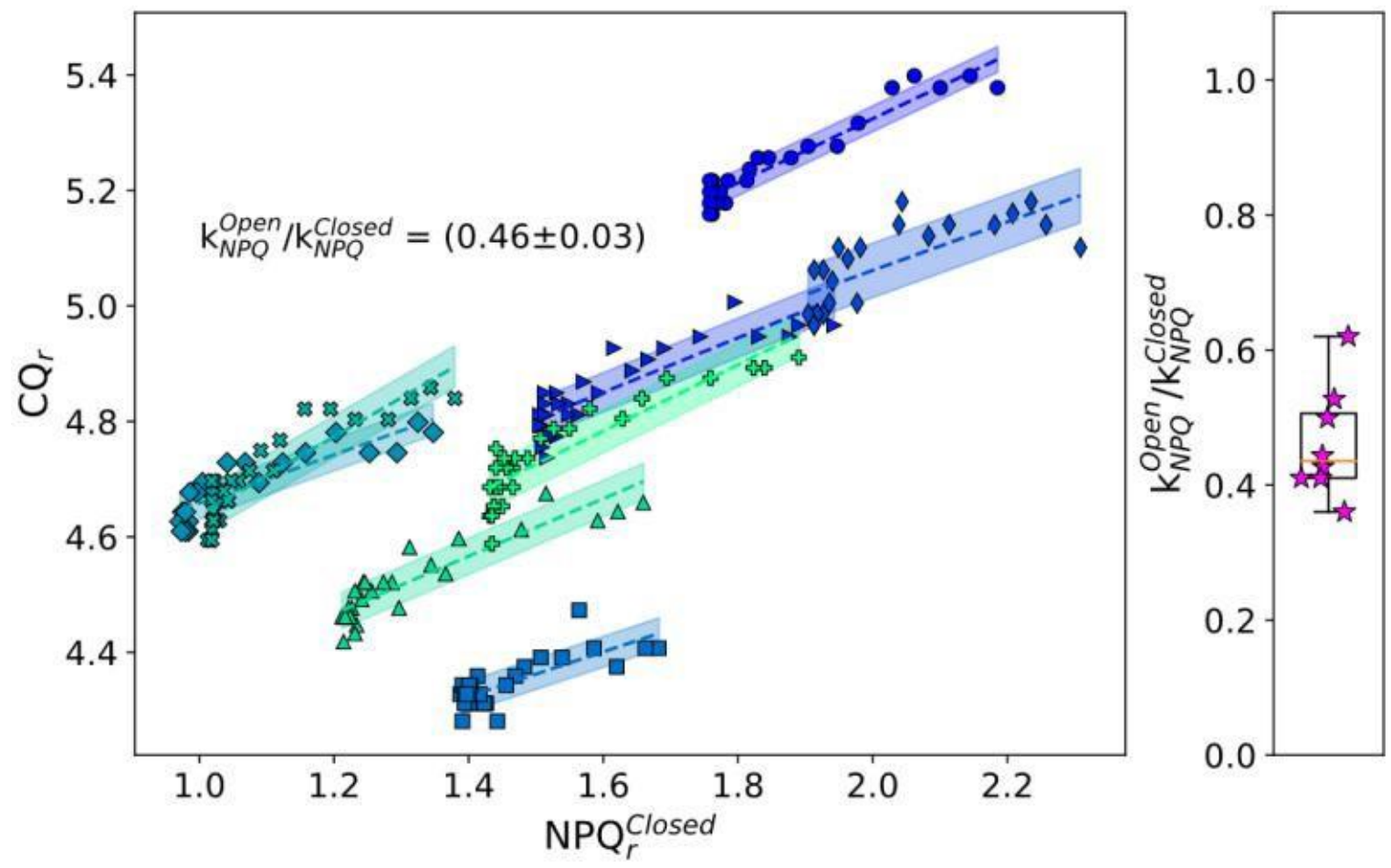

Additional figure 9: Fitted correlation plot of CQ_r vs NPQ^{Closed} (values obtained during the post-illumination recovery period following the light response curve) for *npq4 A. thaliana*. All plants were dark adapted overnight prior to measurement and illuminated with red actinic light.

(No further changes made to manuscript).

Minor point:

Actinic light should be specified for both figures 1 and 4.

Answer: We agree and for figure 1 this is now specified in the figure caption, in figure 4 as these were measured throughout a light response curve the reader is directed to the SI where the light intensities are specified.

We thank the reviewer for their constructive and positive evaluation.

Dear editor,

Thank you very much for giving us the opportunity to submit a revised version of the manuscript NPLANTS-250720396A "Rates of Non-Photochemical Quenching in plants are partially determined by the state of Photosystem II Reaction Centres" by Ramakers, Lennart; Nui, Yuxi; Harbinson, Jeremy; Bader, Arjen; van Amerongen, Herbert.

We appreciate the ongoing effort made by the reviewers and the constructive criticism we received from you and them. We have addressed all the additional comments below and have edited the manuscript to some extent. The final revised version and a revised version with the revisions clearly highlighted/tracked have been uploaded.

We hope that this revised version is now acceptable for publication in Nature Plants. As we indicate in the reply to reviewer 3 we can still upload one part to the supplementary materials but we would like to have your advice on that.

Best regards on behalf of all the authors,

Lennart Ramakers and Herbert van Amerongen

Answers to the referee comments.

Reviewer #1 (Comments for the Author):

However, related to the rather high NPQ determined for wt plants, it was surprising to see in the new Fig. 5 that the PsbS over-expressing line L17 showed a similar or even somewhat lower maximum NPQ value for closed RC than the wt. This is in contrast to the well-known, up to 50-100 % higher NPQ capacity of L17 (e.g. Li et al. 2002). The authors should provide an explanation for this unexpected result to avoid any confusion of the reader.

Answer – We agree with the reviewer that in literature the amount of NPQ induced in the L17 (PsbS overexpressing) plants when compared to wt in the steady state is up to 50-100% higher as shown in the literature (*e.g.* Li *et al.*). If we compare our plants in the steady state, we note that this is indeed the case here, with the L17 plants showing approximately 70% more NPQ than wt consistent with the wider scientific literature (see Figure AA1).

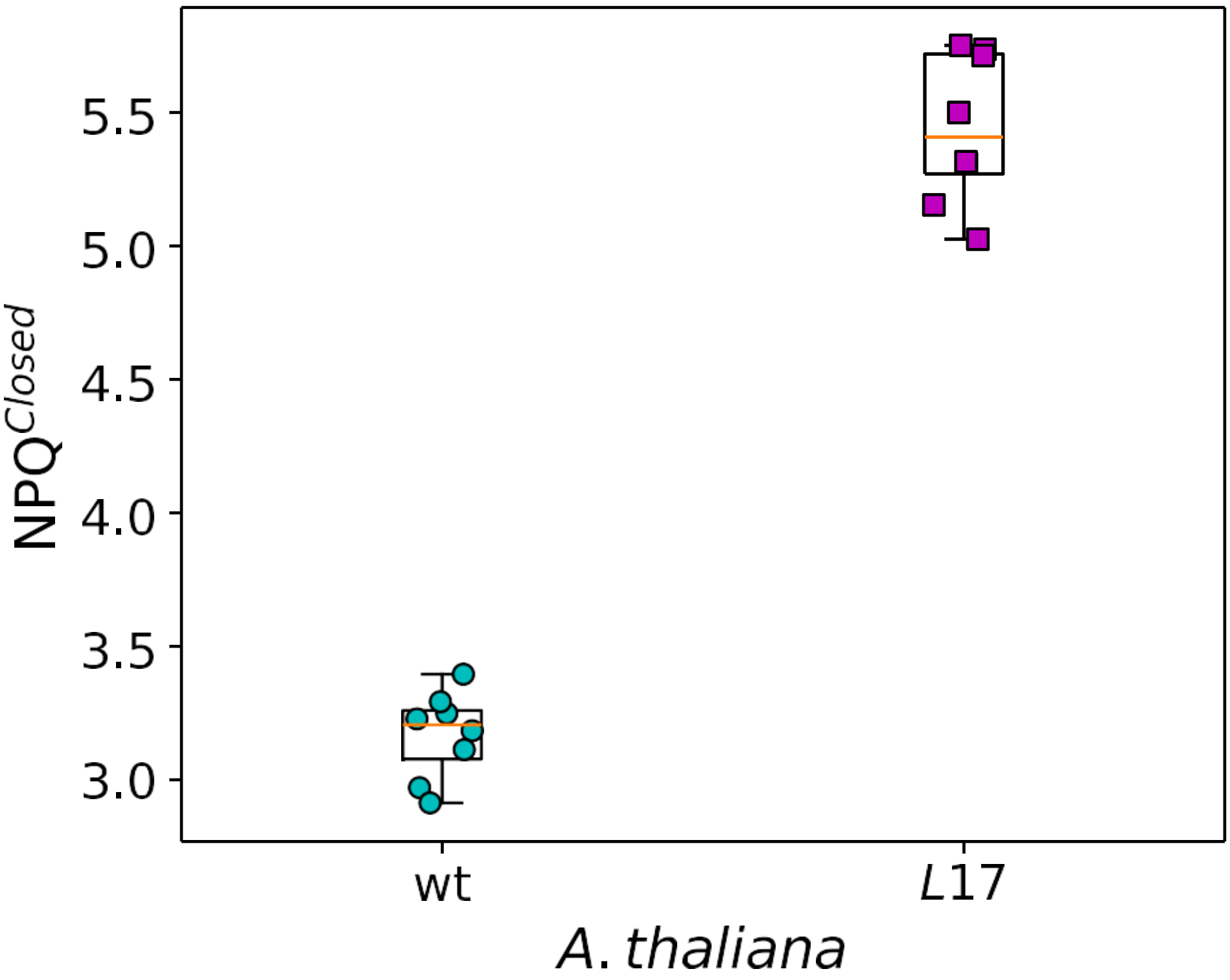

Figure AA1: *Overall Induced NPQ^{Closed} values at the end of the light response curve measurement for wt and L17 A. thaliana. All of the values were calculated at the end of the final 10 minute illumination step in the light response curve. All plants were dark adapted overnight prior to measurement and illuminated with red actinic light. Note that the NPQ values are larger than usually reported in literature because they are corrected for the PSI contribution to the fluorescence.*

However, the NPQ^{Closed} values presented for *L17* in Figure 5 in the current manuscript were obtained during the post-illumination relaxation period following illumination (see also the figure caption) whereas those presented for wt in Figure 4 are obtained during the light response curve measurement (see also the figure caption). This means they are not directly comparable as the NPQ in the L17 plants have already partially relaxed (first pulse in the post-illumination relaxation period was taken after 30s of darkness due to the resolution limit imposed by the well documented 'disruptive' nature of the saturating pulse) and are undergoing further relaxation. This comparison does indeed lead to the somewhat unexpected result pointed out by the reviewer. We have now explicitly stated in the text of the manuscript on page 9: "(Note that the NPQ values of these mutants obtained during the post-illumination recovery period cannot be directly compared with those presented for wt (Fig. 4) as the wt NPQ values were determined at the end of each 10 minute illumination step)".

Reviewer #3 (Comments for the Author):

Comment on the first version:

Here, the authors build on their previous work (Ref 30, Nat Plants 2018), where they initially reported an NPQ component specific to the presence of closed PSII RCs, by quantifying this component and discussing its implications for biomass gains based on NPQ. Evaluation of this component is important to

understanding NPQ, and so this is a worthwhile study. However, given the complexity of the system, isolating a single component is challenging. While the authors have carefully performed a detailed analysis, there are still aspects of the isolation that need additional clarification prior to publication. 1. In the current analysis, the model seems to assume no crosstalk between the NPQ rate and the photochemistry rate, i.e., the 'lake model.' However, as NPQ increases, the rate of NPQ can interfere with the apparent rate of photochemistry even without an additional NPQ component ascribed to the RC, i.e., the 'puddle model.' Prior to publication, the authors need to demonstrate clearly that it is not this type of cross talk that is giving rise to the observed effect.

Comment on the revised version:

The authors have addressed parts of my comments, however major concerns remain. First and foremost, they have not made a compelling case for the need to include an RC-specific component (#4) as they have not shown that it is required to explain the data. Specifically, they do not adequately address the possibility of crosstalk (#1). In their current analysis, they have to make the assumption that the apparent rates are independent, which is implied in their Stern-Volmer equations. However, in a complex kinetic system, even when the microscopic RC trapping rate and the quencher trapping rate are fixed, the total de-excitation rate when both RC and quencher are active is not necessarily the sum of the de-excitation rates of when only one of RC or quencher is active. Without considering this nonlinear effect, they should not conclude that they are quantifying an quenching component in the RC.

Answer:

Apparently last time we did not understand what the reviewer meant exactly, and we are not sure we understand it better this time but we will try again.

Before answering the point of cross-talk we would like to mention that we are not trying to quantify an RC NPQ component, but we are comparing the rate of NPQ in the presence of open and closed RCs and these rates appear to be different. Moreover, for closed RCs the fluorescence spectrum is different in the presence of NPQ and the difference disappears instantaneously upon opening of the RCs (and vice versa), illustrating that there is a fundamental difference in the quenching process(es) in both cases. We then provide a possible explanation (extra NPQ in RCs when they are closed) but we are open for other explanations and suggestions. We propose this (outlined in Martínez-Junza, V. et al. A photoprotection mechanism involving the D2 branch in photosystem II cores with closed reaction centers. Photochemical and Photobiological Sciences 7, 1337–1343 (2008)) as a potential explanation for two reasons, firstly this has been experimentally observed and published and secondly the difference in NPQ aligns neatly with the difference we observe.

Additionally, this potential explanation also nicely explains the results seen in the other NPQ genotypes, which demonstrate a negative correlation between the relative size of the difference in NPQ and the overall amount of induced NPQ. Specifically, as we mention in the manuscript at the end of the discussion:

'The additional quenching via the D2 branch β-carotene in closed PSIIRCs is presumed to be unaffected by PsbS-expression and zeaxanthin levels and suggests the correlation gradients are given by $NPQ^{Open}/NPQ^{Closed} = (NPQ_{Zx} + NPQ_{PsbS})/(NPQ_{Zx} + NPQ_{PsbS} + NPQ_{RC})$. This implies that the ratio should be higher for L17 (which has a large value of $(NPQ_{Zx} + NPQ_{PsbS})$ than wt, increasing

$k_{NPQ}^{Open}/k_{NPQ}^{Closed}$) and lower in both npq1 and npq4 (which have a smaller value of $(NPQ_{Zx} + NPQ_{PsbS})$ than wt, decreasing $k_{NPQ}^{Open}/k_{NPQ}^{Closed}$), fully consistent with the experimental results (Fig.5).'

Here, we note that these experimental results would in our opinion make much less sense in the case of a non-linear interaction model between the RCs and the quenching sites. Specifically, this cross-talk model would presumably mean that more cross-talk would equate to larger differences between the k_{NPQ}^{Open} and k_{NPQ}^{Closed} values we obtain from our analysis. Such models (e.g. the Pauling model for cooperative ligand binding) often include a 'concentration' term for the ligand. Presumably in this case the NPQ quenching site is envisioned to take this role. In that case it would be logical to expect that if there are more of these sites present on average then the cross-talk effect would be larger and so the observed differences between the values k_{NPQ}^{Open} and k_{NPQ}^{Closed} we obtain should be larger. This is inconsistent with the experimental results for L17 A. thaliana. These plants exhibit more NPQ than wt, but we observe a smaller difference between the values k_{NPQ}^{Open} and k_{NPQ}^{Closed} for L17. This problem is further compounded by the fact that we observe larger differences between the values k_{NPQ}^{Open} and k_{NPQ}^{Closed} for both npq4 and npq1. Npq4 and npq1 both have less inducible NPQ (than wt plants) which equates to fewer quenchers being formed and so in the cross-talk model one would expect a smaller effect (again contradicting the experimental results).

The reviewer states that we assume that "there is no crosstalk between the NPQ rate and the photochemistry rate, i.e., the 'lake model'. As the reviewer also states, when NPQ increases, the rate of NPQ can interfere with the apparent rate of photochemistry even without an additional NPQ component ascribed to the RC, i.e., the 'puddle model'. We agree that the lake and puddle models might lead to differences in the amount of NPQ estimated for closed RCs, because of different ways of "extrapolating to 100% closed RCs". Therefore, we have now used both methods to plot NPQ vs. 1-qP (puddle model; using qP as a measure of closed traps, which is correct for the puddle model) and vs. 1-qL (lake model; using qL as a measure of closed traps, which is correct for the lake model). The results are given in Figure AA3:

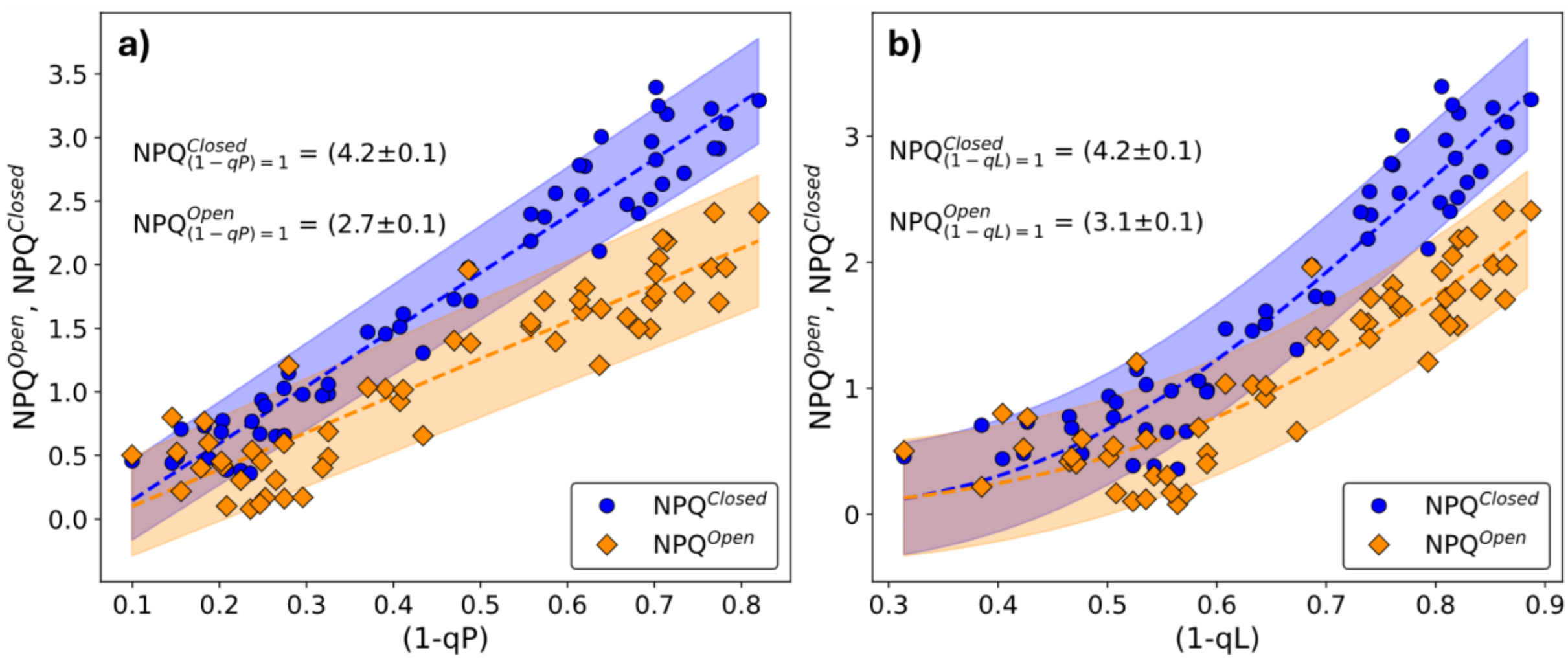

***Figure AA3**: Correlation plots of a) NPQ^{Open} and NPQ^{Closed} vs (1-qP) , including the extrapolated values of NPQ^{Open} and NPQ^{Closed} at (1-qP) = 1, and b) NPQ^{Open} and NPQ^{Closed} vs (1-qL), including the extrapolated values of NPQ^{Open} and NPQ^{Closed} at (1-qL) = 1, for wt A. thaliana.*

Both methods lead to the same conclusion, namely that the rates of NPQ are different for open and closed RCs. Hopefully, this is what the reviewer wanted to see, if not, we would highly appreciate a more detailed specification in the form of a specific model or experimental evidence of this 'cross-talk'. If required, we can include figure AA3 in the SI of the manuscript.
